# Operative vs Nonoperative Treatment for Atraumatic Rotator Cuff Tears: A Trial Protocol for the Arthroscopic Rotator Cuff Pragmatic Randomized Clinical Trial

**DOI:** 10.1001/jamanetworkopen.2019.9050

**Published:** 2019-08-09

**Authors:** Nitin B. Jain, Gregory D. Ayers, Helen Koudelková, Kristin R. Archer, Rebecca Dickinson, Brian Richardson, Marian Derryberry, John E. Kuhn

**Affiliations:** 1 Department of Physical Medicine and Rehabilitation, Vanderbilt University Medical Center, Nashville, Tennessee; 2 Department of Orthopaedics and Rehabilitation, Vanderbilt University Medical Center, Nashville, Tennessee; 3 Department of Biostatistics, Vanderbilt University School of Medicine, Nashville, Tennessee; 4 Stakeholder advisory board, Boston, Massachusetts

## Abstract

**Importance:**

Rotator cuff disorders remain the most common cause of shoulder pain and are among the most common reasons for patients to seek care in primary and specialty settings. Although operative and nonoperative treatments are offered to patients with atraumatic rotator cuff tears, there is a lack of evidence to support operative vs nonoperative treatment. This paucity of evidence has been highlighted by several professional agencies and experts.

**Objective:**

To perform a pragmatic randomized clinical trial, the Arthroscopic Rotator Cuff trial, comparing pain and functional outcomes in patients undergoing operative vs nonoperative treatment for atraumatic rotator cuff tears, and assessing heterogeneity of treatment effects by age and tear size.

**Design, Setting, and Participants:**

Trial protocol of the Arthroscopic Rotator Cuff trial. This pragmatic randomized clinical trial of an estimated 700 patients is adequately powered to accomplish its aims with 488 patients. Primary analysis will be conducted on an intent-to-treat population in the context of a mixed model. The multicenter trial started recruitment in 2018 with a 1-year follow-up duration. Patients aged 50 years or older to younger than 85 years with magnetic resonance imaging–confirmed atraumatic rotator cuff tears that are suitable for either operative or nonoperative treatment will be enrolled. Block randomization will be performed and stratified by site, age, and tear size.

**Intervention:**

Nonoperative treatment consists of an approximately 3-month standardized physical therapy program, whereas operative treatment consists of rotator cuff surgery followed by approximately 4 months of postoperative rehabilitation.

**Main Outcomes and Measures:**

The primary outcome is patient-reported Shoulder Pain and Disability Index score, and the secondary outcome is American Shoulder and Elbow Surgeons Standardized Shoulder Form score measured at 1 year of follow-up.

**Discussion:**

The Arthroscopic Rotator Cuff trial is ongoing, and 12 sites with more than 40 physicians are currently recruiting patients. Although there is variation by site, as of May 2, 2019, 13% of all patients screened (787 of 6293) were eligible for the trial, and 9% of eligible patients (74 of 787) were recruited. Results of this study may help patients, clinicians, and policy makers assess the comparative effectiveness of operative vs nonoperative treatment for atraumatic rotator cuff tears.

**Trial Registration:**

ClinicalTrials.gov identifier: NCT03295994

## Key Points

**Question:**

What is the comparative effectiveness of operative vs nonoperative treatment for rotator cuff tears?

**Findings:**

The clinical trial protocol of a pragmatic randomized clinical trial of an estimated 700 patients (with 488 needed for power calculations) is presented. The primary outcome is patient-reported Shoulder Pain and Disability Index score.

**Meaning:**

The clinical trial is ongoing and will provide data to guide treatment choices for patients with rotator cuff tears.

## Teaser

This is a trial protocol for the Arthroscopic Rotator Cuff trial, an ongoing randomized clinical trial to examine the effectiveness of operative vs nonoperative treatment for atraumatic rotator cuff tears, with patient-reported Shoulder Pain and Disability Index score as the primary outcome.

## Introduction

Shoulder pain accounted for 12.6 million ambulatory care visits to physician offices in 2015 in the United States.^1^ Rotator cuff tears are one of the leading causes of shoulder pain and disability and accounted for 272 148 surgical procedures in 2006.^2,3^ Both nonoperative treatment and surgery are offered to patients with rotator cuff tears, with good outcomes for most patients.^4,5,6,7,8,9,10,11^

The evidence base to support surgical vs nonsurgical treatment for atraumatic rotator cuff tears is small and contradictory.^12,13,14,15,16^ Moosmayer et al^14^ showed a statistically significant improvement in the operative vs the nonoperative group as measured by the shoulder Constant score^17^ and the visual analog pain scale. Moosmayer et al^15^ also recently published results from 2- and 5-year follow-up of this cohort, which showed that differences between the operative and nonoperative groups in an intent-to-treat analysis were not significant. Kukkonen et al^12^ randomized 173 patients with supraspinatus tears into 3 treatment groups: (1) physiotherapy, (2) physiotherapy with acromioplasty, and (3) rotator cuff repair, acromioplasty, and physiotherapy. They reported no statistically significant differences in Constant scores at 12 months of follow-up across the 3 groups. Kukkonen et al^13^ also recently reported their 2-year follow-up results, again showing no difference in clinical outcome among the 3 groups. Lambers Heerspink et al^16^ randomized 56 patients and reported no significant difference between the surgery group and conservative care group at 12 months of follow-up.

The paucity of evidence for operative vs nonoperative treatments for rotator cuff tears is highlighted in the 2012 American Academy of Orthopedic Surgeons Clinical Practice Guidelines,^18^ Cochrane reviews,^19,20^ a report by the Agency for Healthcare Research and Quality,^21^ and expert reviews.^22,23,24,25,26,27,28^ Thus, a well-conducted randomized clinical trial with an adequate sample size is urgently needed.

We are performing a pragmatic randomized clinical trial, the Arthroscopic Rotator Cuff (ARC) trial,^29^ to compare outcomes of operative vs nonoperative treatments for atraumatic rotator cuff tears. The aims of this trial are to compare pain and function in patients undergoing operative vs nonoperative treatment of atraumatic rotator cuff tears at 12 months of follow-up (aim 1) and to assess the associations of rotator cuff tear size and age with comparative outcomes in operative vs nonoperative treatments for atraumatic rotator cuff tears (aim 2). Operative treatment includes rotator cuff surgery followed by postoperative rehabilitation. Nonoperative treatment includes physical therapy only.

## Methods

### Study Design

The ARC trial is a pragmatic randomized clinical trial of an estimated 700 patients (although we are powered to accomplish aim 1 with fewer than 200 patients and aim 2 with 488 patients, as described in the Sample Size Considerations subsection). The trial will compare outcomes of operative vs nonoperative treatments. The full trial protocol is shown in Supplement 1. Blinding to treatment will not be performed given that only 1 group will undergo surgery, and it would be difficult to blind patients and physicians to a surgical intervention. We do not have a placebo or sham surgery group because such a design would make the trial not feasible. Complication risks and postoperative pain experienced after sham arthroscopy are additional concerns.

### Institutional Review Board Approval

Institutional review board (IRB) approval was obtained via the single IRB mechanism SMART IRB, with Vanderbilt serving as the IRB of record.^30^ Participating institutions in the ARC trial ceded reliance to the Vanderbilt IRB for review and approval of the study as well as any subsequent amendments. Written informed consent is obtained from every participant. This report follows the Standard Protocol Items: Recommendations for Interventional Trials (SPIRIT) reporting guideline.

### Eligibility Criteria

 The inclusion criteria for the ARC trial are as follows: age 50 years or older to younger than 85 years; shoulder pain and/or loss of active motion, strength, or function; magnetic resonance imaging (MRI)–confirmed partial- or full-thickness supraspinatus and/or infraspinatus tear of 4 cm or less in longitudinal dimension; medical fitness for surgery (categories I-III per American Society of Anesthesiologists physical status classification)^31^; and ability and willingness to provide informed consent. The pre-MRI exclusion criteria are as follows: a primary diagnosis of something other than a rotator cuff tear, acute rotator cuff tear caused by a severe trauma (as defined later), previous rotator cuff surgery on the affected side, history (in the last 2 years) of shoulder fracture involving the humeral head on affected side, shoulder used as a weight-bearing joint, contraindication to MRI (eg, claustrophobia, pacemaker, pregnancy, or shoulder implant), severe problems with maintaining follow-up expected (eg, history of substance abuse, homelessness or incarceration, dementia, brain injury, or psychotic disorders), and non-English speaking (because questionnaires were validated in English only). The post-MRI exclusion criteria are as follows: glenohumeral osteoarthritis seen on radiographs or MRI, grade 4 fatty infiltration of rotator cuff (any tendons), candidate for reverse shoulder arthroplasty or total shoulder arthroplasty at baseline, and isolated subscapularis and/or teres minor tear on the affected side.

Our interest is in treatment for chronic degenerative cuff tears. Therefore, patients aged 50 years or older will be eligible. Surgery is usually not performed in patients older than 85 years. Acute traumatic tears will be excluded because they are treated surgically as per expert opinion.^25,28^ Acute tears are defined as shoulder symptoms directly related to severe trauma. Because rotator cuff–specific literature on what constitutes trauma is unavailable, we draw from the osteoporosis literature and use the criteria proposed by Mackey et al.^32^ Low-velocity trauma is defined as falls from standing height or less, minimal trauma other than a fall (eg, turning over in bed), and moderate trauma other than a fall (eg, collisions with objects or another person during normal activities). Severe trauma is defined as falls from greater than standing height (eg, falls while standing on a ladder, chair, porch, table, steps, or other raised surface), motor vehicle crashes, being struck by a vehicle or other fast-moving projectile (eg, bullet or baseball), and assault (ie, injuries intentionally inflicted by another person). Patients reporting severe trauma will be excluded, whereas those with low-velocity trauma will still be included because the low-velocity trauma likely exacerbated a preexisting rotator cuff tear. Both partial-thickness and full-thickness tears will be included.

### Recruitment and Participating Sites

The multicenter trial started recruitment in 2018 with a 1-year follow-up duration. Study participants are recruited in sports medicine and shoulder clinics of designated recruiting physicians at each site. Recruiting physicians determine the eligibility of patients to participate in the trial. The diagnosis of an MRI-confirmed rotator cuff tear is required for a patient to be enrolled and randomized. The participating sites are a mix of academic and private practice settings and a combination of urban vs rural or community settings to maximize generalizability. Currently, there are 12 participating sites with more than 40 physicians recruiting for the trial. Although there is variation by site, as of May 2, 2019, 13% of all patients screened (787 of 6293) were eligible for the trial, and 9% of eligible patients (74 of 787) were recruited. Locations of participating sites are accessible via our website.^33,34^

### Randomization

Participants are randomized to receive operative or nonoperative treatment. Randomization is stratified by site, age, and tear size and is blocked within strata using a random sequence of differing block sizes (eg, blocks of 2, 4, and 6 could be used). Randomization strings for each site are unique and are randomly generated using an algorithm and administered in real time using a Research Electronic Data Capture randomization module.^35^

### Baseline Procedures

At baseline, participants complete study questionnaires about pain and movement, shoulder symptoms, daily and recreational activities, general and emotional health, and what treatments have been used to help the participant’s shoulder. Participants also undergo a brief physical examination to measure strength and range of motion. Each participant’s MRI examination is independently read using a standardized form in a blinded fashion by a musculoskeletal radiologist.

### Treatment Protocols

Nonoperative and operative interventions for ARC are consistent with routine clinical standards of care for rotator cuff tears.

#### Nonoperative Intervention

Participants randomized to the nonoperative group of the study follow a prescribed physical therapy and home exercise program. A standardized, nonoperative rehabilitation protocol (eAppendix 1 in Supplement 2) has been developed for this trial with extensive input from our team of clinicians, researchers, and expert consultants. The duration of the nonoperative physical therapy program is approximately 3 months, with participants attending physical therapy 1 to 2 times a week, for a total of approximately 12 to 24 visits. Participants are encouraged to perform exercises at home with a dosing of therapy (physical therapy and/or home exercises) of approximately 4 times per week. The treatment stages of the nonoperative rehabilitation protocol are goal or performance based. Participants in the nonoperative group who make rapid progress may be advanced to the next stage of the rehabilitation treatment protocol if they meet the criteria to do so and may be discharged from physical therapy earlier than 3 months as appropriate. Participants perform a home exercise program during their rehabilitation program and may continue physical therapy beyond 3 months as prescribed or as deemed appropriate by their treating clinicians.

#### Operative Intervention and Postoperative Rehabilitation

Participants randomized to the operative group of the study undergo rotator cuff surgery and then follow a standardized postoperative rehabilitation program. Surgery includes a rotator cuff repair and/or debridement based on an evidence-based surgical protocol (eAppendix 2 in Supplement 2) developed for this trial. Surgery is done almost exclusively on an arthroscopic basis unless the operating surgeon determines on the basis of intraoperative or perioperative factors that an open procedure is required. Information is collected on the specific surgical techniques used, such as number of anchors and type of repair (eg, single row, double row, transosseous, or transtendinous). Any concomitant procedures that are performed, such as subacromial decompression and acromioclavicular joint resection, are also noted. The operating surgeon determines whether a patient may need concomitant or alternate shoulder surgical procedures. The recruiting surgeon completes a standardized postsurgery report form noting details of the surgery and intraoperative observations of tear size, location, and shape; tendon quality and retraction; and cuff repair performed.

After surgery, participants follow a prescribed physical therapy and home exercise program. A standardized, postoperative rehabilitation protocol (eAppendix 3 in Supplement 2) has been developed for this trial with extensive input from our team of clinicians, researchers, and expert consultants. The duration of the postoperative physical therapy program is approximately 4 months, with participants attending physical therapy 1 to 2 times a week, for a total of approximately 16 to 32 visits. Participants are encouraged to perform exercises at home with a dosing of therapy (physical therapy and/or home exercises) of approximately 4 times per week. The treatment stages of the postoperative rehabilitation protocol are linked to specific time frames after surgery. Participants in the operative group who meet treatment goals for a given stage early are not advanced to the next stage ahead of schedule, to allow proper healing of their shoulder postoperatively. Participants perform a home exercise program during their postoperative rehabilitation program and may continue physical therapy beyond 4 months as prescribed or as deemed appropriate by their treating clinicians.

#### Potential Risks From Intervention

Both the operative and nonoperative treatments in this trial are standards of usual care. The adverse events and serious adverse events are therefore those inherent to standard-of-care treatments. Adverse events for this trial include postoperative infection, postoperative bleeding, thromboembolism, nerve injury, complications due to anesthesia, and adhesive capsulitis. Serious adverse events include death and an event requiring hospitalization (in-patient admission) related to the treatment.

#### Cointerventions

Participants are able to take analgesic medications, have shoulder injections, and undergo other nonsurgical interventions, because it would not be ethical to restrict the use of these interventions. Use of cointerventions is recorded in follow-up questionnaires completed by the participant.

### Assessment of Fidelity and Compliance With Rehabilitation Protocols

Patients are asked about their compliance with and frequency of attending physical therapy sessions and home exercises in the 3-, 6-, and 12-month questionnaires. Participants also maintain a self-record of their physical therapy visits, home exercises, and daily shoulder pain using a physical therapy diary. This diary can be completed either on paper or electronically via a smartphone application. Compliance with the rehabilitation program will be assessed in both the nonoperative and postoperative rehabilitation arms. The treating physical therapist completes a standardized report form that notes start and end dates and frequency of physical therapy attendance by the participant and the physical therapist’s compliance with the prescribed protocol.

### Crossover

Participants may cross over from one treatment to another at any point during the trial. Participants are encouraged to stay in their randomized treatment group for at least 6 months to allow the treatment to which they were randomized to be fully effective.

### Follow-up

Participants are followed up via study questionnaires at approximately 3, 6, and 12 months after randomization. Follow-up questionnaires may be completed via a paper copy or an electronic version. Physical therapy diaries are also collected through 12 months.

### Electronic Data Collection and Data Management

We follow Good Trial Practice guidelines for data capture and quality assurance, trial conduct, implementation, analysis, and reporting that were established by Piantadosi^36^ and Meinert^37^ and are now widely acknowledged industry standards. Study data are collected and managed using Research Electronic Data Capture^38^ electronic data capture tools.^39^ Data validation and query management procedures will identify suspicious data through the application of validation rules, generate requests for data review by study sites, and monitor the resolution of these requests. Data will also be reviewed for quality and audited.

### Input From Stakeholders

The ARC trial is built on and conducted with the ongoing input from a stakeholder advisory board. This diverse group includes clinicians, patients, caregivers, industry, payers, and researchers. The stakeholder advisory board was designed to incorporate diverse expertise and the involvement of patients or caregivers who represent the patient population with rotator cuff tears (ie, occupation, comorbidities such as mental health issues, and racial and ethnic diversity). Before funding of the study, the research team partnered with members of the stakeholder advisory board to understand research questions that were important to patients or caregivers and treating clinicians. Since the initiation of the trial, the stakeholder advisory board has continued to meet with the research team approximately once every 2 months. We seek formal feedback from our stakeholders on all aspects of the trial, including recruitment, patient brochures, retention, and website development. Stakeholders are considered team members.

### Outcomes and Measures

#### Primary Outcome

Patients with rotator cuff tear abnormalities present with shoulder pain and loss of function. Hence, our outcomes are patient-reported measures of pain and function. The primary outcome for the trial is shoulder pain and function measured using the Shoulder Pain and Disability Index (SPADI),^40^ a standardized 13-item questionnaire. The SPADI has a pain scale (5 items) and a disability scale (8 items) that are combined to provide a composite score. Score ranges for SPADI are from 0 to 100, with higher scores indicating greater pain and disability. The SPADI demonstrates good reliability and validity,^41,42,43,44,45,46^ and a minimally clinically important difference of 10 points has been described.^46^

#### Secondary Outcome

The American Shoulder and Elbow Surgeons Standardized Shoulder Form (ASES),^47^ an 11-item questionnaire with minor modifications, as described elsewhere,^24^ is the secondary outcome measure for the trial. Score ranges for ASES are from 0 to 100, with higher scores indicating greater pain and disability. The ASES was chosen because it is widely used, is shoulder specific, takes 2 minutes to complete, has an established minimally clinically important difference of more than 9 points,^48^ and has good psychometric properties.^41,49,50,51^

### Statistical Analysis Plan

#### Primary End Point and Analysis Population

The primary outcome measure is the SPADI score, and the primary end point is the change in SPADI score at 12 months relative to baseline. We define the intent-to-treat population as the group of patients randomized to a treatment arm regardless of any other consideration.

#### Primary Analysis

The primary analysis will be conducted on the intent-to-treat population as defined already. Our primary analysis will be conducted in the context of a mixed model. The estimate of interest from this longitudinal model is the predicted differential in 12-month SPADI score change, which is typically estimated as a contrast or difference in predicted 12-month SPADI score change. A directly parallel analysis will also be conducted, where baseline SPADI score will be used as a covariate.

A restricted cubic spline may be fit for continuous covariates to allow for nonlinearity. Furthermore, we will examine different parameterizations of time to appropriately capture longitudinal trends (eg, we might use a time-squared term or let time be categorical). To account for site-to-site variation, the model will include either a fixed effect or random effect for sites. Note that, in a randomized study, the purpose of covariate adjustment in a regression model is to obtain unbiased estimates of effects accounting for the enormous variation typical of longitudinal studies.

#### Heterogeneity of Treatment Effects Analysis and Analysis of Secondary Outcome

The heterogeneity of treatment effects analysis, which is part of the mixed model longitudinal data described already, will assess the association of age (years at randomization) and tear size (centimeters) with treatment on 12-month SPADI score change. These analyses will also be performed for our secondary outcome (ASES score).

### Sample Size Considerations

#### Primary Aim

**Figure 1.**
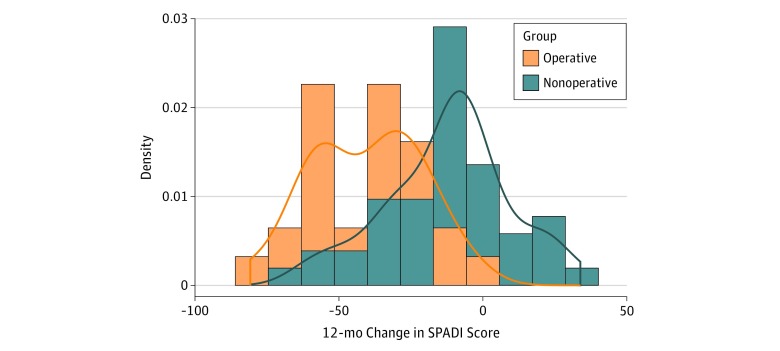
Distributions of 12-Month Change in Shoulder Pain and Disability Index (SPADI) Scores by Treatment Group Density is defined as relative frequency of SPADI scores.

A longitudinal mixed model will be used to compare the 12-month change in SPADI score between treatment groups. The operating characteristics of this plan depend on the marginal distribution of 12-month SPADI score change, the assumed true distributional shift, and observed covariate patterns. Figure 1 displays the marginal distribution of 12-month SPADI score change for patients by treatment group. These distributions were derived from preliminary data taken from an ongoing prospective, nonrandomized cohort of patients with degenerative rotator cuff tears receiving either nonoperative or operative intervention called the Rotator Cuff Outcomes Workgroup study.^52,53,54^ The mean (SD) 12-month SPADI score change was −16.7 (24.2) overall (72 patients). The mean (SD) estimates were −12.3 (21.55) for nonoperative therapy (45 patients) and −40.6 (19.14) for operative treatment (27 patients).^52,53,54^

#### Effect Size and Alternative Hypotheses

We used a 10-unit change in SPADI score as the smallest clinically meaningful change.^14^ In aim 1, we will test the null hypothesis that the 12-month SPADI score change for patients treated operatively vs nonoperatively is equal. As shown later, a sample size of 700 provides outstanding power to detect a 10-unit difference between treatment groups in 12-month SPADI score change from baseline.

#### Sample Size Projections

**Figure 2.**
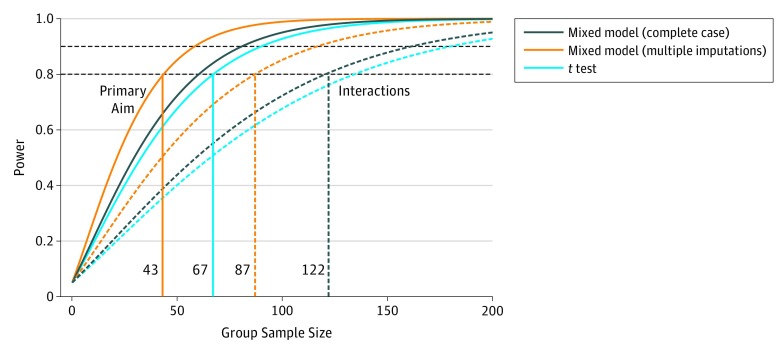
Power Curves for 3 Different Tests for Shoulder Pain and Disability Index Power curves are shown for primary treatment comparison (solid lines) and interactions (dashed lines) from covariate adjusted complete case (navy lines), imputed case (orange lines), and unadjusted (cyan lines) models.

A sample size of 700 participants (350 per treatment group) provides excellent power even with significant levels of dropout or lost-to-follow-up rates (a 2-sided type I error of .05 was used). Figure 2 illustrates the power for 3 different tests of 12-month treatment and SPADI score changes. The cyan line shows the power for a simple *t* test of 12-month SPADI score changes between treatment groups. The navy line shows the power for a least squared means Wald test of the 12-month SPADI score changes between groups but adjusted for baseline covariates (mixed model with complete case analysis). The orange line shows the power for the same test from the mixed model, but when multiple imputation is used to properly account for the large number of missing observations in the preliminary data (relative variance increase, 0.53). Here, relative variance increase is the mean relative increase (calculated for all coefficients) in variance estimates due to missing values. The largest fraction of missing information is 0.412. Interestingly, rather than gaining efficiency, the multiple imputation mixed model appears to be less efficient. Because the multiple imputation model properly accounts for variability in the presence of missingness, we can use this model to anticipate a loss of power due to missing data. The curves shown in Figure 2 are based on the estimated standard deviation from the preliminary data: *t* test on available data (cyan), mixed model on complete case data (navy), and multiply imputed mixed model (orange). The dotted lines show the power to detect the same minimally clinically meaningful change in binary subgroups across treatment groups.^55^ The standard deviations for these curves were similarly derived from preliminary data. This approach allows us to carefully leverage preliminary data to anticipate cluster correlation, covariate effects, missing data patterns, and longitudinal correlation patterns. These projections are conservative.

To be clear, power for the adjusted mixed model reaches 90% at 60 patients per group. With the increased variance from imputation, power reaches 90% with 82 patients in each treatment group. For the unadjusted *t* test, 90% power is achieved with 91 patients per treatment group. For the subgroup and patient heterogeneity analyses in aim 2, 80% power to detect a 2-way interaction as small as the minimally clinically important difference is achieved with 122 patients per group (total of 488) for the imputed data set. Consequently, we expect to have excellent power to detect heterogeneous treatment effects, even in the presence of significant missing data (fraction of missing information, 0.41). In addition, we note that in our preliminary data, the intraclass correlation coefficient for institution was 0.04 (essentially 0) and largely inconsequential, given the longitudinal correlation in SPADI measurements. If necessary, our approach permits the sequestering of site-to-site variability from residual error, increasing power for detecting fixed effects in the data.

### Model Assessment and Sensitivity Analyses

#### Treatment of Crossovers and Missing Data

Although we expect that a significant but manageable number of patients receiving nonoperative treatment will cross over to surgery, our analytic strategies presented here will account for the potential of high crossover rates. We order our analyses as follows. First, our primary model will be a strict intent-to-treat analysis, wherein patients who choose to cross over to surgical treatment (or vice versa) will have outcomes attributed to the treatment group as randomized. Second, we will treat crossover participants as dropouts at the time of crossover and treat the resulting data as missing.^56,57^ Our sample size estimates illustrate that these analyses have good power, even in the presence of significantly high rates of missing or crossover data, and we believe they will provide the best estimates of treatment and covariate effects. Third, we will assign a treatment effect of 0 to each crossover patient. This approach assigns an appropriate penalty to the nonsurgical treatment for having failed that patient. This approach will produce appropriately conservative statistical tests and preserve study power.

#### Assessing Model Fit

The assessment of model fit is indispensable for model development and implementation. Nonlinear predictor rescaling, in the form of restricted cubic splines, will be evaluated and models compared using Akaike criteria. Multivariable association and missing data patterns will be evaluated using trellis graphics and clustering algorithms. Such summary analyses may inform sensitivity analyses with respect to modeling assumptions, rescaling of predictors (eg, restricted cubic splines), and colinearity.^58^ We will repeat these analyses for the secondary outcome (ASES score). Along with standard goodness-of-fit and residual analyses, we will perform model validation and calibration using bootstrap methods^59^ as discussed by Harrell et al.^58,60^

#### Sensitivity Analyses

We will not rely on Gaussian parametric modeling only, because our primary mixed-effects model will be cross-checked using a proportional odds regression comparing treatment effects after adjusting for baseline SPADI score, study site, age, tear size, fatty infiltration, and interactions.^61,62,63^ We will conduct full information analysis and no levels will be combined. In model development, descriptive statistics and graphical displays of outcomes and predictor variables will be examined. Under this category, we will also perform sequential modeling under missing completely at random, missing at random, and missing not at random missing data analyses. We will also perform a sensitivity analysis of the time from baseline to a 10-point (minimally clinically important difference) change in SPADI score using Cox regression, the binary outcome of achieving a 10-point change in SPADI score, and 30% and 50% improvement in outcome scores (success) vs failure using logistic regression. We will repeat sensitivity analyses for the secondary outcome.

### Trial Status

As of May 2, 2019, a total of 74 participants have been recruited in the clinical trial. All 12 sites are actively recruiting, and 10 sites have successfully recruited at least 1 participant. Participants were recruited from among 787 eligible patients, and a total of 6293 patients have been screened for the trial.

## Conclusions

The ARC trial will provide much needed data on operative vs nonoperative treatment for atraumatic rotator cuff tears. Results of this study may help patients, clinicians, and policy makers assess the pivotal question on comparative effectiveness of surgery vs physical therapy for rotator cuff tears.

## Notes

Supplement 1. **Trial Protocol**

Supplement 2. **eAppendix 1.** Nonoperative Rehabilitation Protocol **eAppendix 2.** ARC Surgical Protocol **eAppendix 3. **Postoperative Rehabilitation Protocol
