# Supplementary material for: Operative vs Nonoperative Treatment for Atraumatic Rotator Cuff Tears: A Trial Protocol for the Arthroscopic Rotator Cuff Pragmatic Randomized Clinical Trial
Source: JAMA Netw Open. 2019 Aug 9;2(8):e199050. doi: 10.1001/jamanetworkopen.2019.9050 (PMC6692688; doi:10.1001/jamanetworkopen.2019.9050)
Supplement: Supplement 1. — Trial Protocol [file jamanetwopen-2-e199050-s001.pdf]

ARTHROSCOPIC ROTATOR CUFF TRIAL

Operative versus Non-Operative Treatment for Atraumatic Rotator Cuff Tears: A Multicenter Randomized Controlled Pragmatic Trial

Arthroscopic Rotator Cuff (ARC) Clinical Trial

IRB Protocol Number: 171863

PCORI Contract Number: PCS-1605-35413

Principal Investigator: Nitin Jain, MD, MSPH, Associate Professor,
Department of Physical Medicine & Rehabilitation
Department of Orthopaedics
Vanderbilt University Medical Center

PCORI Program Official: Laura Esmail

Draft or Version Number: 2.0

12 December 2018

PROTOCOL REVISION HISTORY

Version Number	Version Date	Summary of Revisions Made:
0.5	15 Sep 2017	Draft version for DSMC review
0.6	26 Sep 2017	Draft version after DSMC review – clarifications, additions, and corrections
1.0	3 Oct 2017	Version submitted to IRB
2.0	12 Dec 2018	GENERAL: Contact info updated, new study radiologist added, minor qualifications & clarifications added, and correction of minor typos and grammatical errors throughout<br>• **Reasons for withdrawal Revised (5.5.1)** – Randomized subjects who are later found to meet an exclusion criterion will not be withdrawn from the study.<br>• **6-mo PT Report Timepoint Added (6.5.2)** – For subjects who have not started physical therapy by the one-month mark (or for whom treating PT info is not available), an additional PT Report Form will be sent at 6 months.<br>• **Collection of Crossover Treatment Data (6.5.5)** – Provisions added for collection of treatment data for crossover patients when possible/feasible<br>• **PCP/Non-Op Provider Referrals (7.1)** – Provision added for recruitment via referral by PCPs / Non-operative providers to study physicians (as appropriate)<br>• **Follow-up (7.4.1 & 2)** – Clarification added regarding what is/is not a protocol deviation with regards to patient follow-up and treatment timeline<br>• **Physical Therapy Diary (7.4.2)** – Provision added to collect PT Diary info from crossover/delayed treatment subjects at 12 months as appropriate<br>• **Protocol Deviation Clarified (16.6)** – Definition revised to accord with guidelines of the reviewing, single IRB (VUMC) and distinction added between protocol deviations and protocol violations.<br>• **Appendices -** List of appendices updated to include new study documents and reorganized to group related documents together by type

FUNDING ACKNOWLEDGEMENTS

Research reported in this publication was partially funded through a Patient-Centered Outcomes Research Institute (PCORI) Award (1605-35413). The statements presented in this work are solely the responsibility of the author(s) and do not necessarily represent the views of the Patient-Centered Outcomes Research Institute (PCORI), its Board of Governors or Methodology Committee.

Research reported in this publication was supported by the National Institute of Arthritis and Musculoskeletal and Skin Diseases of the National Institutes of Health under award number U34AR069201. The content is solely the responsibility of the authors and does not necessarily represent the official views of the National Institutes of Health.

The publication described was supported by CTSA award No. UL1TR000445 from the National Center for Advancing Translational Sciences. Its contents are solely the responsibility of the authors and do not necessarily represent official views of the National Center for Advancing Translational Sciences or the National Institutes of Health.

STATEMENT OF COMPLIANCE

The study will be conducted in accordance with the International Conference on Harmonisation guidelines for Good Clinical Practice (ICH E6), the Code of Federal Regulations on the Protection of Human Subjects (45 CFR Part 46), and PCORI Terms of Award. All personnel involved in the conduct of this study have completed human subjects protection training and Good Clinical Practice (GCP) training.

SIGNATURE PAGE

The signature below constitutes the approval of this protocol and the attachments and provides the necessary assurances that this trial will be conducted according to all stipulations of the protocol, including all statements regarding confidentiality, and according to local legal and regulatory requirements and applicable US federal regulations and ICH guidelines.

To be signed by all of the following:

- Principal Investigator
- Co-Investigators
- Clinical Site Investigators (Site PIs)
- DSMC Members

ARTHROSCOPIC ROTATOR CUFF TRIAL

"Operative versus Non-Operative Treatment for Atraumatic Rotator Cuff Tears: A Multicenter Randomized Controlled Pragmatic Trial"

Protocol Version 2.0

12 DEC 2018

I have read and understand the information in this Study Protocol and will ensure that all associates, colleagues, and employees assisting in the conduct of the study are informed about the obligations incurred by their contribution to the study.

Signed: ______________________________ Date: ______________

Name:

Title:

Site/Location:

Study Role:

TABLE OF CONTENTS

PAGE
PROTOCOL REVISION HISTORY I
FUNDING ACKNOWLEDGEMENTS II
STATEMENT OF COMPLIANCE II
SIGNATURE PAGE III
TABLE OF CONTENTS IV
LIST OF ABBREVIATIONS VIII
PROTOCOL SUMMARY X
1 KEY ROLES AND CONTACT INFORMATION 12
2 INTRODUCTION: BACKGROUND INFORMATION AND SCIENTIFIC RATIONALE 22
2.1 Background Information 22
2.2 Rationale 22
2.3 Hypotheses 23
2.4 Potential Risks and Benefits 23
2.4.1 Potential Risks 23
2.4.2 Potential Benefits 24
3 OBJECTIVES 25
3.1 Study Objectives 25
3.2 Study Outcome Measures 25
3.2.1 Primary Outcome 25
3.2.2 Secondary Outcome 25
4 STUDY DESIGN 26
4.1 Primary Study 26
4.2 Sub-study 26
5 STUDY ENROLLMENT AND WITHDRAWAL 27
5.1 Subject Inclusion Criteria 27
5.2 Subject Exclusion Criteria 29
5.3 Strategies for Recruitment and Retention 30
5.3.1 Recruitment / Consent Videos 31
5.3.2 Crossover 31
5.3.3 Compensation 31
5.3.4 Long-term Participation Strategies 32
5.3.5 Sharing Results with Participants 33
5.4 Treatment Assignment Procedures 33
5.4.1 Randomization Procedures 33
5.4.2 Masking Procedures 34
5.5 Subject Withdrawal 34
5.5.1 Reasons for Withdrawal 34
5.5.2 Handling of Subject Withdrawals or Subject Discontinuation of Study Intervention 34

5.6 Premature Termination or Suspension of Study 35
6 STUDY INTERVENTION 36
6.1 Study Procedural Intervention(s) Description 36
6.1.1 Non-operative Intervention 36
6.1.2 Operative Intervention 36
6.2 Concomitant Medications/Treatments 37
6.3 Administration of Procedural Intervention 37
6.3.1 Surgery 37
6.3.2 Physical Therapy 37
6.3.3 Home Exercise Program 38
6.4 Procedures for Training of Clinicians on Procedural Intervention 38
6.4.1 Treating Surgeons 38
6.4.2 Treating Physical Therapists 39
6.4.3 Subjects 39
6.4.4 Research Staff 39
6.5 Assessment of Clinician and/or Subject Compliance with Study Procedural Intervention 40
6.5.1 Treating Surgeons 40
6.5.2 Treating Physical Therapists 40
6.5.3 Subjects 40
6.5.4 MRI Review by Study Radiologist 41
7 STUDY SCHEDULE 42
7.1 Pre-Screening (Prior to Clinic Visit) 42
7.2 Screening (During Clinic Visit) 42
7.3 Enrollment/Baseline (During &/or After Clinic Visit) 43
7.4 Follow-Up 46
7.4.1 Follow-Up Questionnaires (± 4-6 weeks from follow-up time point) 46
7.4.2 Physical Therapy Diary (for duration of prescribed physical therapy program) 48
7.5 Follow-Up Visit & MRI (2-7 years after treatment for sub-study) 49
8 STUDY PROCEDURES / EVALUATIONS 51
8.1 Study Procedures/Evaluations 51
9 ASSESSMENT OF SAFETY 53
9.1 Specification of Safety Parameters 53
9.1.1 Unanticipated Problems 53
9.1.2 Adverse Events (AEs) 53
9.1.3 Serious Adverse Events (SAEs) 53
9.2 Time Period and Frequency for Event Assessment and Follow-Up 54
9.3 Characteristics of an Adverse Event 54
9.3.1 Relationship to Study Intervention 54
9.3.2 Expectedness of Event 54

9.3.3 Severity of Event 55
9.4 Reporting Procedures 55
9.4.1 Related SAEs & Safety Issues 55
9.4.2 AEs & Unrelated SAEs 55
9.4.3 Unanticipated Problems 56
9.5 Halting Rules 56
10 STUDY OVERSIGHT 57
10.1 Data and Safety Monitoring Committee (DSMC) 57
10.2 Scientific Advisory Board 57
10.3 Study Steering Committee 57
10.4 Stakeholder Advisory Board 57
11 CLINICAL SITE MONITORING 58
11.1 Screening/Enrollment Monitoring Reports 58
11.2 Site Monitoring Calls 58
11.3 Site Visits 58
12 STATISTICAL CONSIDERATIONS 59
12.1 Study Hypotheses 59
12.2 Final Analysis Plan 59
12.2.1 Primary Analysis 59
12.2.2 Secondary Analyses 61
12.3 Sample Size Considerations 62
12.3.1 Primary Aim 62
12.3.2 Effect Size and Alternative Hypotheses 62
12.3.3 Sample Size Projections 63
12.3.4 Safety Review 64
12.3.5 Efficacy Review 64
12.4 Model Assessment and Sensitivity Analyses 64
12.5 Sub Study 65
13 SOURCE DOCUMENTS & ACCESS TO SOURCE DATA/DOCUMENTS 66
13.1 Source Documents 66
13.2 Maintenance of and Access to Source Documents 67
13.2.1 Clinical Screening Forms 67
13.2.2 Consent Forms 67
13.2.3 Paper Case Report Forms 68
13.2.4 Electronic Case Report Forms 68
13.2.5 MRI / X-Ray Images 69
14 QUALITY CONTROL AND QUALITY ASSURANCE 70
14.1 Electronic Data Capture (REDCap) 70
14.2 Data Security 70
14.3 Data Entry 70
14.4 Staff Training and Tracking 70

14.5 Standard Operating Procedures (SOPs) 71
14.6 Data Review 71
14.7 Data Validation and Query Management 71
14.8 Quality Review and Data Auditing 72
15 ETHICS/PROTECTION OF HUMAN SUBJECTS 73
15.1 Ethical Standard 73
15.2 Institutional Review Board 73
15.3 Informed Consent Process 73
15.4 Exclusion of Women, Minorities, and Children (Special Populations) 74
15.5 Subject Confidentiality 74
16 DATA HANDLING AND RECORD KEEPING 75
16.1 Data Management Responsibilities 75
16.1.1 Recruiting Sites 75
16.1.2 Coordinating Center (VUMC) 76
16.2 Data Capture Methods 76
16.3 Types of Data 77
16.4 Schedule and Content of Reports 77
16.4.1 Monthly Screening, Enrollment and Data Report (every month) 77
16.4.2 Interim Progress Reports to PCORI (every 6 months) 77
16.4.3 Data Monitoring Reports to DSMC (every 6-12 months) 77
16.4.4 Final Progress Report to PCORI (at conclusion of study) 77
16.5 Study Records Retention 78
16.6 Protocol Deviations and Violations 78
16.6.1 Protocol Deviations 78
16.6.2 Protocol Violations 79
17 PUBLICATION / DATA SHARING POLICY 80
17.1 Publication Guidelines 80
18 LITERATURE REFERENCES 81
SUPPLEMENTAL MATERIALS 84
APPENDICES 85
APPENDIX A: SCHEDULE OF EVENTS 87

LIST OF ABBREVIATIONS

AE	Adverse Event
ASA	American Society of Anesthesiologists
ASES	American Shoulder and Elbow Surgeons
CFR	Code of Federal Regulations
CONSORT	Consolidated Standards of Reporting Trials
CRF	Case Report Forms
DAG	Data Access Group
DCC	Data Coordinating Center
DSMC	Data & Safety Monitoring Committee
DSMP	Data & Safety Monitoring Plan
eCRF	Electronic Case Report Form
FWA	Federalwide Assurance
GCP	Good Clinical Practice
HEP	Home Exercise Program
HIPAA	Health Insurance Portability and Accountability Act
ICF	Informed Consent Form
ICH	International Conference on Harmonisation
ICMJE	International Committee of Medical Journal Editors
IRB	Institutional Review Board
ISM	Independent Safety Monitor
ITT	Intent-to-Treat Analysis
MD	Medical Doctor / Physician
MOON	Multicenter Orthopedic Outcomes Network
MOOP	Manual of Operating Procedures
MRI	Magnetic Resonance Imaging
N	Number (typically refers to subjects)
NIH	National Institutes of Health
OHRP	Office for Human Research Protections
PCORI	Patient-Centered Outcomes Research Institute
PCP	Primary Care Provider
PHI	Protected Health Information
PI	Principal Investigator

PT	Physical Therapy
QA	Quality Assurance
QC	Quality Control
RA	Research Assistant/Research Coordinator
REDCap	Research Electronic Data Capture
ROM	Range of Motion
DASH	Disabilities of Arm, Shoulder, and Hand Questionnaire
SAE	Serious Adverse Event
SOP	Standard Operating Procedure
SPADI	Shoulder Pain and Disability Index
UP	Unanticipated Problem
US	United States
WHO	World Health Organization

PROTOCOL SUMMARY

Title: Operative versus non-operative treatment for atraumatic rotator cuff tears: A multi-center pragmatic randomized controlled trial

Précis: This is a randomized controlled trial in patients with atraumatic rotator cuff tears. The study will recruit 700 patients randomized to arthroscopic surgery versus physical therapy treatments. The primary study and analysis will compare pain and function (measured by SPADI) at one (1) year follow-up.

Objectives: To compare pain and function in patients undergoing operative versus non-operative treatment of atraumatic rotator cuff tears at 12 months of follow-up

To assess effects of rotator cuff tear size and age on comparative outcomes (measured by SPADI) in operative versus non-operative treatments for atraumatic rotator cuff tears at 12 months of follow-up

Primary Outcome: SPADI

Secondary: ASES

Population: Patients of age ≥50 to <85 years diagnosed with a rotator cuff tear (with MRI confirmation) who are fit for either arthroscopic rotator cuff surgery or physical therapy will be recruited.

Sample Size: 700 subjects

Phase: III

Number of Sites: 12

Description of Intervention: Operative intervention includes arthroscopic rotator cuff surgery followed by post-operative physical therapy. Non-operative intervention includes physical therapy only.

Study Duration: Approximately 5 years for the primary study

Estimated Time to Complete Enrollment: Approximately 3.5 years

Schematic of Study Design:

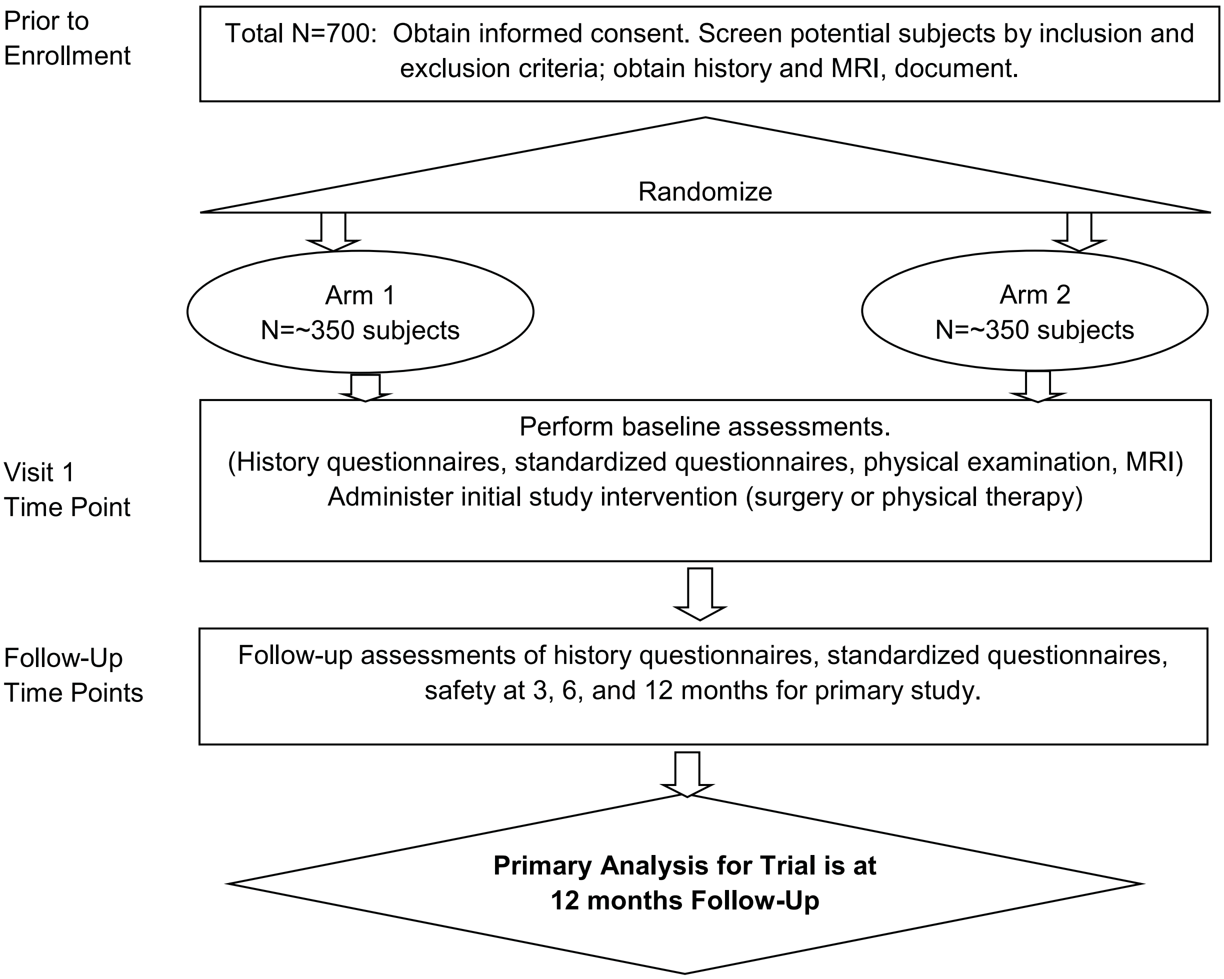

1 KEY ROLES AND CONTACT INFORMATION

Principal Investigator:

Nitin Jain, MD, MSPH
Associate Professor of Physical Medicine & Rehabilitation
Associate Professor of Orthopaedics
Associate Professor of Medicine, Vanderbilt Epidemiology Center
Vanderbilt University Medical Center
Vanderbilt Stallworth Rehabilitation Hospital
Physical Medicine & Rehabilitation
2201 Children's Way, Suite 1318
Nashville, TN 37212
nitin.jain@vumc.org
P: (615) 936-8508
F: (615) 322-7454

PCORI Program Official:

Laura Esmail, PhD, MSc
Program Officer
Patient-Centered Outcomes Research Institute
Science, Clinical Effectiveness and Decision Science
1828 L Street, NW, Suite 900
Washington, DC 20036
lesmail@pcori.org
P: (202) 827-7700 ext 176

Clinical Site Investigators:

Keith M. Baumgarten, MD (Site 4 - Orthopedic Institute)
Title: Clinical Orthopedic Physician
Orthopedic Institute
810 East 23rd St.
Sioux Falls, SD 57117
kbaumga@yahoo.com
P: (605) 977-6848

Carolyn Hettrich, MD, MPH (Site 8 - University of Kentucky)
Title: Associate Professor, Department of Orthopaedic Surgery
Orthopaedic Surgery & Sports Medicine
740 S. Limestone, Suite K401
Lexington, KY 40536
carolyn.hettrich@gmail.com
P: (206) 356-3356

John D. Kelly, IV, MD (Site 10 - University of Pennsylvania)
Title: Associate Professor of Clinical Orthopaedic Surgery
Director, Sports Shoulder
University of Pennsylvania
Dept. of Orthopaedic Surgery
34th and Spruce St.
Philadelphia, PA 19104
johndak4@gmail.com
P: (215) 615-4400

Michael Khazzam, MD (Site 11 - University of Texas Southwestern)
Title: Associate Professor of Orthopaedic Surgery
Shoulder Service
Department of Orthopaedic Surgery
UT Southwestern Medical Center
1801 Inwood Rd.
Dallas, Texas 75390-8883
michael.khazzam@UTSouthwestern.edu
P: (214) 645-8917
F: (214) 645-3323

C. Benjamin Ma, MD (Site 5 - University of California San Francisco)

Title: Professor in Residence, Orthopaedic Surgery,

Chief of Sports Medicine and Shoulder Service

UCSF Orthopaedic Institute

1500 Owens Street, Suite 200

San Francisco CA 94158

maBen@ucsf.edu

P: (415) 353-7586

F: (415) 885-3838

Eric McCarty, MD (Site 6 - University of Colorado)

Title: Chief of Sports Medicine and Shoulder Surgery

University of Colorado Sports Medicine

2150 Stadium Drive

Boulder, CO 80309

eric.mccarty@ucdenver.edu

P: (720) 848-1900

Bruce S. Miller, MD, MS (Site 9 - University of Michigan)

Title: Associate Professor, Department of Orthopaedic Surgery

Medical Director, Medsport Sports Medicine – Shoulder & Knee Surgery

University of Michigan Orthopaedic Surgery

24 Frank Lloyd Wright Dr.

Ann Arbor MI 48106

bsmiller@med.umich.edu

P: (734) 232-9603

Andrew Nevaiser, MD (Site 3 - Ohio State University)

Title: Associate Professor of Clinical Orthopaedics, Division of Shoulder

Ohio State Sports Medicine Center

2835 Fred Taylor Drive

Columbus, Ohio 43202

aneviaser@gmail.com

P: (614) 293-3600

F: (614) 293-2910

Matthew V. Smith, MD, MSc (Site 12 - Washington University)

Title: Assistant Professor of Orthopedic Surgery

Washington University and Barnes-Jewish Orthopedic Center

14532 South Outer Forty Dr.

Chesterfield, MO 63017

smithmv@wudosis.wustl.edu

P: (314) 514-3500

Edwin E. Spencer, Jr., MD (Site 2 – Knoxville Orthopaedic Clinic)

Title: Orthopedic Surgeon

Knoxville Orthopaedic Clinic

256 Fort Sanders West Boulevard Suite 200

Knoxville, TN 37922

spencershoulder13@gmail.com

P: (865) 558-4400

F: (865) 769-4557

Brian R. Wolf, MD, MS (Site 7 - University of Iowa)

Title: Ralph and Marcia Congdon Professor of Orthopaedic Surgery

Director, University of Iowa Sports Medicine,

Head Team Physician, University of Iowa Athletics,

Associate Professor of Orthopedics and Rehabilitation,

Associate Professor of Physical Therapy and Rehabilitation Science

UI Sports Medicine Center

2701 Prairie Meadow Dr.

Iowa City, IA 52242

brian-wolf@uiowa.edu

P: (319) 353-7954

F: (319) 353-6754

Rick W. Wright, MD (Site 12 - Washington University)

Title: Jerome J. Gilden Distinguished

Professor of Orthopaedic Surgery,

Executive Vice Chairman, Department of Orthopaedic Surgery

Washington University School of Medicine

Department of Orthopedic Surgery

Campus Box 8233

660 S. Euclid Ave.

Saint Louis, MO 63110

rwwright1@aol.com

P: (314) 747-2639

F: (314) 747-2599

Institutions:

Vanderbilt University Medical Center (Site 1 & Coordinating Center)

1301 Medical Center Dr.

Nashville, TN 37232

P: (615) 322-5000

PI: Nitin Jain, MD, MSPH

Knoxville Orthopaedic Clinic (Site 2)

260 Fort Sanders West Boulevard, Building #6

Knoxville, TN 37922

P: (865) 558-4400

PI: Edwin E. Spencer, Jr., MD

The Ohio State University Wexner Medical Center (Site 3)

410 W 10th Avenue

Columbus, OH 43210

P: (614) 293-8000

PI: Andrew Nevaiser, MD

Orthopedic Institute (Site 4)

810 East 23rd St.

Sioux Falls, SD 57117

P: (605) 331-5890

PI: Keith M. Baumgarten, MD

University of California San Francisco Medical Center (Site 5)
505 Parnassus Avenue
San Francisco, CA 94143
P: (415) 476-1000
PI: C. Benjamin Ma, MD

University of Colorado Denver (Site 6)
1201 Larimer Street
Denver, CO 80204
P: (303) 315-5969
PI: Eric McCarty, MD

University of Iowa (Site 7)
Iowa City, IA 52242
P: (319) 335-3500
PI: Brian R. Wolf, MD, MS

University of Kentucky Research Foundation (Site 8)
102 Kinkead Hall
Lexington, KY 40506-0057
P: (859) 257-8288
PI: Carolyn Hettrich, MD, MPH

University of Michigan (Site 9)
500 S. State Street
Ann Arbor, MI 48109
P: (734) 764-1817
PI: Bruce S. Miller, MD, MS

University of Pennsylvania (Site 10)
Philadelphia, PA 19104
P: (215) 898-5000
PI: John D. Kelly, IV, MD

University of Texas Southwestern Medical Center (Site 11)
5323 Harry Hines Blvd.
Dallas, TX 75390
P: (214) 648-3111
PI: Michael Khazzam, MD

Washington University in St. Louis (Site 12)
1 Brookings Drive
St. Louis, MO 63130
P: (314) 362-5000
PI: Matthew V. Smith, MD, MSc
Co-PI: Rick W. Wright, MD

Other Core Personnel:

John E. Kuhn, MD, MS (Co-Investigator, Surgical Core Leader)
Kenneth D. Schermerhorn Professor of Orthopaedic Surgery,
Chief of Shoulder and Sports Medicine
Vanderbilt Ortho-Sports Medicine
1215 21st Ave. South, Suite 4200
Medical Center East, South Tower
Nashville, TN 37232-8774
j.kuhn@vumc.org
P: (615) 322-7878
F: (615) 322-7126

Kristin R. Archer, PhD, DPT (Co-Investigator, Rehabilitation Core Leader)

Associate Professor, Orthopaedic Surgery & Physical Medicine and Rehabilitation

Director of Research Division, Trauma & Spine Research

Vanderbilt Ortho-Sports Medicine

1215 21st Ave. South, Suite 4200

Medical Center East, South Tower

Nashville, TN 37232-8774

kristin.archer@vumc.org

P: (615) 322-2732

F: (615) 875-1079

Gregory D. Ayers, MS (Co-Investigator, Principal Statistician)

Faculty, Senior Associate in Biostatistics

Vanderbilt University School of Medicine

Division of Cancer Biostatistics

2220 Pierce Ave., Suite 571

Preston Research Building

Nashville, TN 37232-6848

dan.ayers@vumc.org

P: (615) 343-9180

F: (615) 936-2602

Marian Derryberry, M.Ed., Ed.S. (Co-Investigator/Patient Partner, Stakeholder Advisory Board Chair)

1600 Washington St. Ph 4

West Newton, MA 02465

mderryberry@gmail.com

P: (617) 480-9190

Jeffrey D. Blume, PhD (Consultant Statistician)

Associate Professor of Biostatistics

Director of Graduate Studies

Vanderbilt University School of Medicine

Department of Biostatistics

2525 West End Ave., Suite 11000

Nashville, TN 37203

j.blume@vumc.org

P: (615) 343-9267

F: (615) 343-4924

David S. Taber, MD (Radiology Core Lead)

Assistant Professor of Radiology and Radiological Sciences

Assistant Professor of Emergency Medicine

Vanderbilt University School of Medicine

Department of Radiology

1161 21st Ave. South

Medical Center North, CCC-1118

Nashville, TN 37232-2675

david.taber@vumc.org

P: (615) 875-1885

F: (615) 322-3764

2 INTRODUCTION: BACKGROUND INFORMATION AND SCIENTIFIC RATIONALE

2.1 Background Information

Shoulder symptoms accounted for 11.5 million ambulatory care visits in 2010 in the United States[1]. Rotator cuff tears are one of the leading causes of shoulder pain and disability and accounted for an estimated 272,148 ambulatory surgeries in 2006[2 3]. Despite the lack of evidence on operative versus non-operative treatment for rotator cuff tears, there has been an increase in surgery as the initial treatment for rotator cuff tears over time and billions of dollars spent by our healthcare system on these treatments[4]. This lack of evidence and the urgent need for research in this area is highlighted by the American Academy of Orthopaedic Surgeons[5], Agency for Healthcare Research and Quality[6], and Cochrane reviews[7 8].

We will compare operative and non-operative treatment in patients with atraumatic rotator cuff tears. Operative treatment includes arthroscopic rotator cuff surgery followed by post-operative physical therapy. Non-operative treatment includes physical therapy (without surgery).

2.2 Rationale

Our interest is in treatment for chronic degenerative rotator cuff tears. Therefore, patients ≥50 years of age will be eligible. Acute traumatic tears will be excluded because they are treated surgically as per expert opinion[9 10]. Since rotator cuff specific literature on what constitutes trauma is unavailable, we draw from the osteoporosis literature and use the criteria proposed by Mackey et al.[11] Patients reporting severe trauma will be excluded whereas those with low velocity trauma will still be included since the low velocity trauma likely exacerbated a pre-existing rotator cuff tear. Both partial-thickness and full-thickness tears will be included since the dilemma of operative versus non-operative treatment applies to both settings. Patients with Grade IV fatty infiltration will not be included since these patients may not be candidates for surgery in clinical practice. Patients with isolated subscapularis tears will also be excluded since these tears are usually traumatic and treated surgically. Detailed inclusion and exclusion criteria are presented in Sections 5.1 & 5.2.

Both operative and non-operative treatments are standards of usual care. Operative treatment will include arthroscopic rotator cuff surgery. The standards of care/protocol for operative treatment are included in Supplemental Materials. As in usual clinical practice, patients will have post-operative physical therapy after surgery. The post-operative physical therapy lasts for approximately 4 months. A standardized post-operative physical therapy regimen for this trial is included in Supplemental Materials.

Patients randomized to non-operative treatment will only receive physical therapy. This usually lasts for approximately 3 months. A standardized non-operative physical therapy regimen for this trial is included in Supplemental Materials.

2.3 Hypotheses

Our hypotheses for the primary analysis at year one follow-up are:

Hypothesis 1: Patients undergoing operative treatment will have significantly greater improvement in pain reduction and function (measured by baseline to 12 month change in SPADI)[12] as compared with those treated non-operatively at 12 months of follow-up.

Hypothesis 2a: Operative treatment will be more effective than non-operative treatment in cuff tears of increasing size.
Hypothesis 2b: Operative treatment will be more effective than non-operative treatment of rotator cuff tears with decreasing age.

2.4 Potential Risks and Benefits

2.4.1 Potential Risks

Potential physical risks include:

- fatigue due to answering study questionnaires
- pain and discomfort during the physical exam

The medical risks of participating in this study are those inherent to assigned treatments which are standards of care.

- For **physical therapy**, the risks include increased pain or no improvement of pain and function, and adhesive capsulitis (frozen shoulder).
- For **surgery**, the risks include those commonly associated with surgery, such as: post-operative bleeding or infection, blood clot, hospital readmission, complications related to anesthesia, and, in rare cases, death. Other risks for surgical treatment of rotator cuff tear include: nerve injury, adhesive capsulitis (frozen shoulder), and no improvement of pain & function.

MRI Risks: There are no known major risks associated with an MRI scan. But, it is possible that harmful effects could be discovered in the future. Known risks

include claustrophobia, nausea, dizziness, flashing lights in eyes, and metallic taste.

In addition, there may be risks associated with the study that are unknown or unexpected.

2.4.2 Potential Benefits

There are no direct benefits to subjects participating in the study. Knowledge gained about the effectiveness of surgery versus non-operative treatments may benefit future patients and providers.

3 OBJECTIVES

3.1 Study Objectives

Aim 1: To compare pain and function in patients undergoing operative versus non-operative treatment of atraumatic rotator cuff tears at 12 months of follow-up.

Aim 2: To assess effects of rotator cuff tear size and age on comparative outcomes (measured by SPADI) in operative versus non-operative treatments for atraumatic rotator cuff tears.

3.2 Study Outcome Measures

Patients with rotator cuff tear present with shoulder pain and loss of function. Hence, our outcomes are patient-reported measures of pain and function.

3.2.1 Primary Outcome

The primary outcome measure for this study is the difference between baseline and 12 month SPADI[12].

3.2.2 Secondary Outcome

ASES[13] will be used as a secondary outcome.

4 STUDY DESIGN

4.1 Primary Study

This is an unblinded, multi-center, pragmatic randomized controlled clinical trial of operative versus non-operative treatments for atraumatic rotator cuff tears. A sample size of 700 patients will be enrolled with approximately equal numbers of patients randomized to operative treatment (surgery + post-op physical therapy) and non-operative treatment (physical therapy only without surgery). Both treatments are current standards of care. The duration of enrollment is expected to last approximately 3.5 years. For the main study and analysis, a 12-month follow-up is needed, with the duration of the primary study lasting for approximately 5 years.

Upon enrollment at baseline, patients will fill out study questionnaires, undergo a brief physical examination, and, if applicable, have a research MRI. Follow-up for the primary study will be performed via questionnaires at 3, 6, and 12 months.

4.2 Sub-study

A sub-study will follow subjects after the primary end point of one (1) year for the trial has been met. The sub-study will follow subjects via questionnaires at yearly intervals for up to 10 years (i.e., for 9 years after the primary end point). The sub-study may also include a physical examination and research MRI at 2-7 years interval from enrollment. The objective of the sub-study is to assess longer-term patient-reported outcomes of operative and non-operative treatments (measured by SPADI and ASES) in patients with rotator cuff tears. It will also assess whether structural changes such as tear size and fatty infiltration of the rotator cuff muscle are different in the operative and non-operative groups.

5 STUDY ENROLLMENT AND WITHDRAWAL

**Target Sample Size:**	700 participants
**Gender:**	Both genders
**Age:**	≥50 years to <85 years
**Demographic Group:**	All demographic groups included
**General Health Status:**	Patients with shoulder pain and atraumatic rotator cuff tear

Screening to achieve 700 participants:

- Expected number pre-screened: 175,000 (250 for every 80 evaluable patients)
- Expected number screened in clinic: 56,000 (80 for every 8 eligible)
- Expected number eligible for study: 5,600 (8 for every 1 recruited)

5.1 Subject Inclusion Criteria

In order to be eligible to participate in this study, an individual must meet all of the following criteria:

- Aged ≥50 years to <85 years
- Shoulder pain and/or loss of active motion, strength or function
- MRI-confirmed partial- or full-thickness supraspinatus and/or infraspinatus tear of 4cm or less in longitudinal dimension
- Medically fit for surgery, defined as Category I-III per American Society of Anesthesiologists (ASA) Physical Status Classification (see Table 1: ASA Physical Status Classifications and Examples)
- Ability and willingness to provide informed consent

TABLE 1. ASA Physical Status Classifications and Examples

ASA Physical Status Classification	Definition	Examples
**ASA I**	A normal healthy patient	Healthy, nonsmoking, no or minimal alcohol use
**ASA II**	A patient with mild systemic disease	Mild diseases only without substantive functional limitations. Examples include (but not limited to): current smoker, social alcohol drinker, pregnancy, obesity (30<BMI<40), well-controlled DM/HTN, mild lung disease
**ASA III**	A patient with severe systemic disease	Substantive functional limitations; one or more moderate to severe diseases. Examples include (but not limited to): poorly controlled DM or HTN, COPD, morbid obesity (BMI ≥ 40), active hepatitis, alcohol dependence or abuse, implanted pacemaker, moderate reduction of ejection fraction, ESRD undergoing regularly scheduled dialysis, premature infant PCA <60 wk, history (>3 mo) of MI, CVA, TIA or CAD/stents
**ASA IV**	A patient with severe systemic disease that is a constant threat to life	Examples include (but not limited to): recent (<3 mo) MI, CVA, TIA or CAD/stents; ongoing cardiac ischemia or severe valve dysfunction; severe reduction of ejection fraction; sepsis; DIC; ARD; or ESRD not undergoing regularly scheduled dialysis
**ASA V**	A moribund patient who is not expected to survive without the operation	Examples include (but not limited to): ruptured abdominal/thoracic aneurysm, massive trauma, intracranial bleed with mass effect, ischemic bowel in the face of significant cardiac pathology or multiple organ/system dysfunction

ASA Physical Status Classification	Definition	Examples
**ASA VI**	A declared brain-dead patient whose organs are being removed for donor purposes	
ARD, acid reflux disease; ASA, American Society of Anesthesiologists; BMI, body mass index; CAD, coronary artery disease; COPD, chronic obstructive pulmonary disease; CVA, cerebral vascular accident; DIC, disseminated intravascular coagulation, DM, diabetes mellitus, ESRD, end-stage renal disease; HTN, hypertension; MI, myocardial infarction; PCA, postconceptural age; PS, physical status; TIA, transient ischemic attack		

5.2 Subject Exclusion Criteria

Any individual who meets any of the following criteria will be excluded from participation in this study:

- Primary diagnosis is something other than a rotator cuff tear
- History (in last 2 years) of shoulder fracture involving the humeral head on affected side
- Previous rotator cuff surgery on affected side
- Isolated subscapularis &/or teres minor tear on affected side
- Acute rotator cuff tear caused by a severe trauma (see TABLE 2: Trauma Classification)
- Shoulder used as a weight-bearing joint
- Contraindication to MRI (claustrophobia, pacemaker, pregnancy, shoulder implant, etc.)
- Glenohumeral osteoarthritis on xrays/MRI, as determined by recruiting MD
- Grade 4 fatty infiltration of rotator cuff (any tendons)

- Candidate for reverse shoulder arthroplasty or total shoulder arthroplasty at baseline
- Non-English speaking (questionnaires only validated in English)
- Severe problems with maintaining follow-up expected (such as but not limited to history of substance abuse, homelessness/incarceration, dementia, brain injury, and psychotic disorders)

Patients receiving workers' compensation are not excluded from the trial.

TABLE 2: Trauma Classification (adapted from Mackay, et. al., 2007)

Low Velocity Trauma	Severe Trauma
Falls from standing height or less	Falls from greater than standing height (e.g., falls while standing on a ladder, chair, porch, table, steps, or other raised surface)
Minimal trauma other than a fall (e.g., turning over in bed)	Motor vehicle crashes
Moderate trauma other than a fall (e.g., collisions with objects or another person during normal activities)	Struck by vehicle or other fast-moving projectile (e.g., bullet, baseball, etc.)
	Assault (i.e., injuries intentionally inflicted by another person)

5.3 Strategies for Recruitment and Retention

Study participants will be recruited through the clinics of designated recruiting physicians at each site. Potential recruits may be identified through other mechanisms (e.g. primary care provider or non-operative provider referral, patient self-referral after hearing about the study, advertising, etc.) and referred to the clinics of recruiting

physicians for evaluation. Patients in these clinics will be screened to determine eligibility and, if eligible, approached for enrollment in the study. Patients may also be contacted prior to their clinic visit to make them aware of the study.

5.3.1 Recruitment / Consent Videos

Patients will typically be shown two short, standardized videos:

1) Recruitment Video – This video provides information about the trial (purpose and design), what is involved (length and procedures), and participant compensation.

2) Consent Video – This video provides information about risks and benefits, withdrawal/removal from the study, and information that will be collected.

Use of these videos can help ensure consistency of information presented to study candidates across all sites. The recruitment video has been designed to educate participants about the purpose of the trial and what it involves as well as the importance of randomized assignment and following through with their assigned treatment for 6 months.

The RAs and recruiting MD will also introduce the study to the patient via sample standardized scripts and be available to answer questions that the patient may have. In the event that the patient does not wish to watch the videos but wants to participate in the study, the RA may substitute a verbal discussion of the content using the video scripts as a guide.

5.3.2 Crossover

Subjects may cross over from one treatment to another at any point during the trial. Crossovers are usually from the non-operative to the operative arm (although a few patients randomized to the operative arm may not have surgery and have only physical therapy). It takes about 6 months for non-operative physical therapy to be effective. Hence, at the time of recruitment, subjects will be encouraged to stay in their randomized treatment arm for at least 6 months. However, subjects may cross over at any time point if they choose to do so and will still remain in the study.

If a subject crosses over within 6 months following randomization, or does not pursue either treatment within 12 months after randomization, it will be considered a protocol deviation.

5.3.3 Compensation

Subjects will be compensated for the time and inconvenience of participating in the study. They will be paid:

- $100 for the baseline enrollment visit,
- $50 for each follow-up questionnaire completed (at 3mo, 6mo, 12mo for the main study, and annually in years 2-10 for the sub-study), and
- $100 for a return visit & MRI for the sub-study (once, approximately 2-7 years after treatment for the sub-study).
- Subjects who undergo a screening MRI at baseline for research purposes will be paid $100 for the MRI visit.

In a subset of patients, a research MRI to determine eligibility for the study may be offered to the subject as determined by the recruiting physician. Those subjects who receive a screening MRI for research purposes will be paid $100 for the time and inconvenience of returning for the screening MRI.

Main Study						Sub-Study									
Screening MRI (if applicable)	Base-line Visit	3 Mo	6 Mo	12 Mo		2 Yr	3 Yr	4 Yr	5 Yr	6 Yr	7 Yr	8 Yr	9 Yr	10 Yr	Return Visit & MRI
$100	$ 100	$50	$50	$50		$50	$50	$50	$50	$50	$50	$50	$50	$50	$100

5.3.4 Long-term Participation Strategies

Several strategies will be used to promote continued participation.

- **Complete Contact Information** - Both primary and secondary addresses, phone numbers, and emails will be collected for subjects at baseline as well as on all follow-up questionnaires. If subjects live at an alternate address for part of the year, it will be noted which months are spent at each address.
- **Multiple Means of Contact** – Subjects will be contacted by phone, email, mail, or text as needed. Subjects can specify their preferred means of contact. Alternate means of contact may be used if initial attempts to reach the subject via their preferred means are unsuccessful.
- **Secondary Contact Person** – The name, relationship, address, phone, and email of a secondary contact person will be collected at baseline as well as on all follow-up questionnaires. This person will be contacted if all other attempts to reach the subject fail.

- **Option of Paper or Electronic Questionnaires** – Subjects may choose to complete follow-up questionnaires either on paper or electronically (via tablet or computer) per their preference.

- **Follow-Up Reminders & PI calls** – Subjects who do not complete/return follow-up questionnaires within the expected timeframe will receive follow up reminders. If questionnaires are not completed/returned after at least three reminders by study personnel, subjects may receive a call from the study PI or site PI.

5.3.5 Sharing Results with Participants

Results of the study will be made available on the study website: *www.shoulderstudy.org/studies/arc-trial*.

5.4 Treatment Assignment Procedures

Enrolled participants will be assigned to treatment with approximately equal numbers of subjects randomized to operative and non-operative treatment. Subjects will be randomized ONLY after eligibility has been confirmed and informed consent has been obtained.

Randomization will be automatically assigned within REDCap after strata information has been entered. Randomization is initiated manually by study personnel. The database has been programmed so that randomization is only possible if all necessary information has been entered and all eligibility criteria have been documented as met.

5.4.1 Randomization Procedures

Randomization will be stratified by site, age class (< 65 years; ≥ 65 years), and tear size class (> 0 < 2 cm; ≥ 2 ≤ 4 cm) and blocked within strata using a random sequence of differing block sizes (i.e., 2, 4 and 6). Accordingly, the maximum treatment assignment imbalance at any site is 3 participants. Randomization strings for each site will be unique, randomly generated, and administered in real time using a randomization module within REDCap.

The study principal investigator, trial coordinator, and site investigator will receive automatically generated emails when a participant is randomized.

Randomization assignments will be accessible 24 hours a day, 7 days a week via REDCap. If REDCap is unavailable locally for any reason, site personnel will call the study coordinator at the coordinating center to complete randomization. If REDCap

is down globally, randomization will not take place until the system is back up, or the subject affected will not be randomized.

Additional placeholder sites have been included in the randomization table, so that new sites or replacement sites may easily be added to the randomization module in REDCap (if needed) once the study is under way.

5.4.2 Masking Procedures

Intervention arms will not be masked, as this is a non-blinded study.

5.5 Subject Withdrawal

Subjects may withdraw from the study voluntarily or be terminated by the principal investigator.

5.5.1 Reasons for Withdrawal

Subjects are free to withdraw from participation in the study at any time upon request.

An investigator may terminate a subject's participation in the study if:

- Any serious adverse event (SAE) or other medical condition or situation occurs such that the subject cannot continue or continued participation in the study would not be in the best interest of the subject.
- The subject is deceased.
- The subject does not complete any follow-up questionnaires in the first year.
- The subject does not complete annual follow-up questionnaires two years in a row for the sub-study.
- The study ends earlier than anticipated.

5.5.2 Handling of Subject Withdrawals or Subject Discontinuation of Study Intervention

Subjects who are withdrawn from the study, either through voluntary discontinuation, for failure to complete study questionnaires, or by decision of the principal investigator for reasons enumerated above, will be recorded as terminated. They will be sent a letter confirming their early termination from the study and will no longer be

followed. They will not be sent any further follow-up questionnaires or brought back for a follow-up visit & MRI (if applicable).

If a subject is withdrawn due to an SAE or other medical condition that precludes their continuation in the study, the study or site PI will call the subject to discuss and ensure appropriate medical treatment or follow up is arranged.

The study sample size has been calculated to allow for attrition due to early withdrawal or discontinuation.

5.6 Premature Termination or Suspension of Study

The study may be suspended or prematurely terminated if there is sufficient reason or cause. Per the Data & Safety Monitoring Committee (DSMC) charter, the DSMC will monitor progress of the trial and determine any actions related to suspension or termination of the trial. (See Supplemental Materials)

Written notification, documenting the reason for study suspension or termination, will be provided by the suspending or terminating party to the investigator, funding agency, and regulatory authorities. If the study is prematurely terminated or suspended, the principal investigator will promptly inform the IRB and will provide the reason(s) for the termination or suspension.

6 STUDY INTERVENTION

6.1 Study Procedural Intervention(s) Description

Patients will be randomized to either an operative or non-operative treatment regimen. Non-operative and operative interventions performed for this study are consistent with routine clinical standards of care for rotator cuff tears.

6.1.1 Non-operative Intervention

Subjects randomized to the non-operative arm of the study will follow a standardized non-operative rehabilitation program. They will not undergo rotator cuff surgery (unless subjects cross over).

Non-operative Rehabilitation Regimen: Subjects randomized to the non-operative arm of the study will follow a prescribed (approximately 3-month) physical therapy and home exercise program to strengthen and stabilize the shoulder muscles. A standardized, non-operative rehabilitation protocol (see Supplemental Materials) has been developed for this trial with extensive input from our team of clinicians, researchers, and expert consultants. Subjects may continue physical therapy beyond 3 months as prescribed/deemed appropriate by their treating clinicians.

6.1.2 Operative Intervention

Subjects randomized to the operative arm of the study will undergo surgery to debride or repair the rotator cuff tear and then follow a standardized post-operative rehabilitation program.

Surgery: The subject's attending surgeon will perform a rotator cuff repair or debridement based on the evidence-based surgical protocol (see Supplemental Materials) developed for this trial. This protocol has been developed to maximize the potential for rotator cuff tear repair healing and to reduce the variables that could affect surgical outcomes.

Information will be collected on the specific surgical techniques used, such as number of anchors and type of repair (e.g., single row, double row, transosseous, or transtendinous). Any concomitant procedures such as subacromial decompression and acromioclavicular joint resection will also be noted. (See Appendix, Surgery Report Form.)

Post-Operative Rehabilitation Regimen: Following surgery, subjects will follow a prescribed (approximately 4-month) physical therapy and home exercise program to strengthen and stabilize the shoulder muscles. A standardized, post-operative rehabilitation protocol (see Supplemental Materials) has been

developed for this trial with extensive input from our team of clinicians, researchers, and expert consultants. Subjects may continue physical therapy beyond 4 months as prescribed/deemed appropriate by their treating clinicians.

The post-operative rehabilitation regimen is similar to the one that will be followed for non-operative rehabilitation. The main difference between the two intervention arms is the rotator cuff surgery.

6.2 Concomitant Medications/Treatments

Subjects will be able to take analgesic medications or have shoulder injections, since it would not be ethical to restrict the use of these interventions. Use of co-interventions will be recorded in follow-up questionnaires completed by the subject.

6.3 Administration of Procedural Intervention

Interventions will be standardized to the extent this is possible. Standardized protocols have been developed for surgery, as well as for both non-operative and post-operative rehabilitation regimens, and will be disseminated to treating clinicians.

6.3.1 Surgery

Study surgeons at each recruiting site will administer the surgical rotator cuff repair/debridement for those subjects in the operative arm of the study recruited from their site. Surgery should be scheduled to occur within 8 weeks after randomization to the operative treatment arm. If surgery occurs later than 8 weeks after randomization, it will be considered a protocol deviation.

6.3.2 Physical Therapy

Local physical therapists will administer the post-operative and non-operative rehabilitation regimens for subjects in both arms of the study. Site coordinators will assist in referring subjects to a physical therapist at a location that is convenient for them. Preferably, this should be a physical therapist familiar with the study or a physical therapist at one of the designated local PT networks for each site, whenever feasible. Subjects may also utilize any physical therapist of their choice.

Physical therapy should begin within 8 weeks after randomization to the non-operative treatment arm or within 8 weeks following surgery, if in the operative arm. If physical therapy begins later than this time period after either assignment to the non-operative arm or undergoing surgery, it will be considered a protocol deviation.

Non-Operative Physical Therapy: The duration of the non-operative physical therapy program will be approximately 3 months, with subjects attending physical therapy 1-2 times a week on average, for a total of approximately 12-24 visits.

The treatment stages of the non-operative rehabilitation protocol are goal/performance based. Non-operative subjects who make rapid progress may be advanced to the next stage of the rehabilitation treatment protocol if they meet the criteria to do so and may be discharged from physical therapy earlier than 3 months as appropriate.

Post-operative Physical Therapy: The duration of the post-operative physical therapy program will be approximately 4 months, with subjects attending physical therapy 1-2 times a week on average, for a total of approximately 16-32 visits.

The treatment stages of the post-operative rehabilitation protocol are pegged to specific timeframes following surgery. Subjects in the operative arm who meet treatment goals for a given stage early should NOT be advanced to the next stage ahead of schedule, in order to allow proper healing of their shoulder post-op. Rather, the interval between physical therapy visits should be increased as appropriate.

The actual number and frequency of physical therapy visits will vary over the course of treatment and by subject, based on individual progress and needs.

6.3.3 Home Exercise Program

Subjects themselves will be responsible for attending scheduled physical therapy treatments and adhering to the home exercise program as prescribed by their physical therapist until discharged from physical therapy.

To be considered compliant with the rehabilitation regimen for either arm of the study, subjects are expected to attend physical therapy &/or complete their home program exercises a total of at least three times each week. The three times can be any combination of the two (e.g., 2 physical therapy visits + 1 day of home exercises, 1 physical therapy visit + 2 days of home exercises, and so on).

6.4 Procedures for Training of Clinicians on Procedural Intervention

6.4.1 Treating Surgeons

All study surgeons will be briefed on the standardized surgical protocol that has been developed with physician input and will follow the surgical protocol.

6.4.2 Treating Physical Therapists

To ensure that treating physical therapists – many of whom will be otherwise unacquainted with the study – are aware of the subject's participation in and the requirements of the study, each recruiting site will have a designated Lead Physical Therapist. His/her job will be to contact the treating physical therapist for all subjects recruited from their site as soon as possible after treating physical therapist contact information is obtained, ideally within 2-3 weeks *after randomization* (for the non-operative arm) or within 2-3 weeks *after surgery* (for the operative arm).

The site Lead Physical Therapist will ensure that the treating physical therapist receives a copy of the appropriate rehabilitation protocol (operative or non-operative), will explain the details of the study, will stress the importance of protocol adherence, and will answer any questions the treating physical therapist has regarding the treatment protocol. Treating physical therapists will be encouraged to call their Lead Physical Therapist at any time they have a question or concern during the course of treatment.

6.4.3 Subjects

Subjects will be provided with a Rotator Cuff Home Exercise Program (see Appendix) that contains various exercises which may be assigned to them during their home exercise program. They will be instructed to bring this brochure with them to each physical therapy visit so that their physical therapist can update the assignment of exercises and the set/reps, frequency, etc. for each exercise at each visit. Exercises that no longer need to be performed will be crossed off as finished/discharged. Proper use of the patient exercise brochure will help to ensure that subjects know exactly which exercises they should be doing (in what amount and with what frequency) at any given time and will promote compliance with the home program.

6.4.4 Research Staff

Research staff will not administer treatment interventions but will be trained by their site physical therapist and provided written instructions on how to perform a physical exam in clinic to measure the strength of both shoulders for each subject (using a dynamometer). In some cases, a physician may designate staff member (e.g., a Physician's Assistant or qualified delegate) to perform the range of motion portion of the physical exam.

6.5 Assessment of Clinician and/or Subject Compliance with Study Procedural Intervention

A systematic effort will be made to obtain relevant information regarding clinician and/or subject compliance with study interventions through the means outlined below. However, failure to obtain this information after reasonable follow-up attempts will not be considered a protocol deviation, except in the case of patient questionnaires.

6.5.1 Treating Surgeons

Following rotator cuff surgery, surgeons will complete a Surgery Report Form (CRF-05, see Appendix) detailing the type of tear, the repair/procedures performed, number of anchors used, tendon quality and retraction, etc. A copy of the surgical note from the subject’s medical record will be submitted as well. An effort will be made to also collect this information for crossover patients when possible/feasible.

6.5.2 Treating Physical Therapists

Typically, treating physical therapists will complete a Physical Therapist Report Form (CRF-06, see Appendix) twice over the course of the subject’s rehabilitation program – The first report will be sent approximately one month after the patient is randomized (for non-op patients) or one month after surgery (for operative patients). The second will be sent approximately two months later, at the three-month benchmark. If a patient has not started physical therapy by the one-month mark (or contact info for the treating physical therapist was not available at that time), an additional report will be sent at the 6-month mark. The report form will capture information about the subject’s physical therapy start and end dates and which exercises are being/have been performed with the subject. Subjects will sign an authorization form allowing treating physical therapists to share this information with the study research team. If any problems or issues of concern are identified at the first report time point, the site Lead Physical Therapist will contact the treating physical therapist in order to address them.

For crossover patients, an effort will be made to obtain physical therapy treatment information for the crossover treatment arm when possible/feasible. This may include retrospective collection of available data (e.g., if PT treatment is already complete or nearly so) and/or prospective data collection similar to that for the assigned treatment arm (e.g., if PT treatment is still in the early stages).

6.5.3 Subjects

Subjects will complete a Physical Therapy Diary (CRF-04, see Appendix) – on paper or via the MyCap smartphone app - to record on which days they attended physical therapy (PT) &/or performed their home exercise program (HEP). Diary tracking will begin on the date of the subject’s first physical therapy visit after either

randomization (for the non-operative treatment group) or rotator cuff surgery (for the operative group) and will officially end when the subject is discharged from physical therapy.

On follow-up questionnaires, subjects will report the date of their shoulder surgery (if applicable) and answer questions regarding their physical rehabilitation program (e.g., start & end dates of PT, average frequency of PT attendance and of performance of HEP).

For crossover patients, an effort to obtain subject PT info will be made when possible/feasible by providing a second version of the PT Diary for tracking physical therapy & home exercises for the crossover treatment arm.

6.5.4 MRI Review by Study Radiologist

The study radiologist will review the MRI images and, if available, x-rays of all randomized subjects from all recruiting sites to verify the presence and size of a rotator cuff tear for each subject's affected shoulder. To eliminate possible bias, all images will be de-identified prior to being reviewed (except in cases where images are unable to be de-identified because of technical software issues). Only the subject's study ID will link the images to the subject. The reviewing radiologist will complete an MRI Review Form (CRF-07, see Appendix) for each subject.

If a subject is found to have no rotator cuff tear or a tear larger than 4cm, or if the radiologist identifies anything of concern other than a rotator cuff tear (e.g., acute fracture, tumor, etc.), the study PI is to be notified immediately. If an area of concern is identified, the study PI will notify the treating/recruiting physician. If the subject does not have a rotator cuff tear, or has a tear larger than 4cm, a re-check (by the study radiologist) that will override the original read will be requested to verify that the initial reading is accurate. Regardless of the results of the MRI review, the subject will not be withdrawn and will continue to be followed for the duration of the study.

7 STUDY SCHEDULE

7.1 Pre-Screening (Prior to Clinic Visit)

Potential subjects will be pre-screened prior to their clinic visit via a review of the clinic schedules of participating study physicians or, in some cases, of referring non-operative providers. Those who meet the study age range, present with a shoulder complaint, and are not returning for a post-op visit will be identified as potentially eligible. Some sites may wait until patients have had an MRI of their shoulder before including them in pre-screening. (Subject Locator, or similar institutionally-approved software applications at recruiting sites, may be used to facilitate the process of identifying potential participants.) If no obvious exclusionary factors are found in a review of available medical records, identified subjects will be screened in clinic by study MDs to determine study eligibility.

PCPs or non-operative specialty providers (such as sports medicine MDs and physiatrists) may refer patients to the clinics of participating study physicians as appropriate for further evaluation.

Activities:

- Review clinic schedules to identify potentially eligible subjects
- PCP/non-op provider referrals to study physicians (as appropriate)
- Review available medical records for any obvious exclusionary factors
- Create a Clinic Screening Form (CRF-00) for potentially eligible subjects with no known exclusionary factors
- Deliver Clinic Screening Form (CRF-00) to examining physician prior to potential subject's clinic visit
- Enter preliminary screening information into REDCap Screening Log

7.2 Screening (During Clinic Visit)

Potentially eligible subjects will be evaluated in clinic based on the physician's clinical examination, clinical MRI results, and screening questions that address the study inclusion and exclusion criteria using the Clinic Screening Form (CRF-00, see Appendix).

Subjects may receive a screening MRI for research purposes in order to determine eligibility. Screening research MRIs may be ordered at the discretion of the recruiting

physician. Although there is no attempt to standardize an MRI protocol for those that are ordered for clinical care, a protocol has been developed for research MRIs. (See Supplemental Materials)

Patients receiving a screening MRI will be consented before the procedure and provisionally enrolled in the trial (including completion of baseline measurements), but will not be randomized to treatment until their eligibility has been confirmed by MRI. The same is true for otherwise eligible subjects with pending MRIs who will not return to clinic in person for their MRI results.

Activities:

- Clinic Screening Form (CRF-00) completed by physician for potentially eligible subjects to determine eligibility
- Obtain and document consent from potential subjects who will receive screening MRI
- Schedule screening MRI for individuals who will have one
- Complete Screening Log records for ineligible subjects

7.3 Enrollment/Baseline (During &/or After Clinic Visit)

Subjects who meet all eligibility criteria per the Clinic Screening Form (CRF-00) will be approached for participation in the trial. The MD will briefly introduce the study. If the patient is interested in learning more, a trained research assistant/coordinator will present the study using a sample script and standardized recruitment and consent videos, if the patient agrees to watch them. Subjects who consent to participate in the trial and be randomized will sign a written consent form and be given a baseline questionnaire to complete while in clinic – either on paper or electronically on a tablet or computer (per the subject’s preference). Study staff will also perform a brief physical exam to measure and record the subject’s strength and range of motion in both arms.

Study staff will review the completed questionnaire for completeness and confirm the subject’s willingness to be randomized. If eligible for the study, the subject will then be randomized to treatment via the REDCap randomization module and either scheduled for surgery or referred for physical therapy as appropriate.

Subjects will also receive a study packet with information about the trial, a copy of their signed consent form, a physical therapy protocol for their assigned arm of the study to give to their treating physical therapist (if subject is being randomized that day in clinic) , a patient exercise brochure to use in completing their home exercise program, and a physical therapy diary for tracking their rehabilitation program. Subjects who elect to

complete their physical therapy diary via smartphone app will be assisted by study staff to install and set up the MyCap app before they leave the clinic.

Subjects who want more time to consider participation in the study will be provided contact information for the site coordinator/RA in case they decide to participate or have questions. If they later choose to participate, they would return in person to be consented and to complete enrollment procedures (physical exam, baseline questionnaire, and randomization). Study coordinators will follow up with patients after approximately one week, if the patient has not yet reached out to either decline or express interest in the study.

In cases where a subject lives far away or it is not feasible to return in person to receive their MRI results, a physician may choose instead to call and discuss the subject's MRI results with them over the phone. Subjects with a pending MRI who meet all other pre-MRI eligibility criteria and will not return to clinic in person to receive their MRI results may be approached about the study before leaving the clinic. Those who agree to participate in the trial if their pending MRI confirms they meet eligibility requirements will be consented and complete the baseline questionnaire and physical exam. They will be paid for the baseline/enrollment visit and will be provisionally enrolled in the study but will not be randomized to treatment unless and until their eligibility is confirmed by MRI. Once their eligibility has been verified, the subject's willingness to be randomized will be confirmed by phone prior to randomizing them to treatment via the REDCap randomization module.

Subjects who consent and are enrolled prior to MRI confirmation of their eligibility and are subsequently determined to be ineligible will be counted as screen-fails. They will not be randomized to treatment or followed thereafter and will not count toward the total accrual numbers for the trial.

Potential subjects who meet all eligibility criteria (including MRI confirmation of tear):

- Approach patient in clinic and provide information about the trial
- Obtain and document consent from subjects who agree to participate
- Have subject complete authorization form allowing treating physical therapist to share information about PT Treatment (see Appendix)
- Administer physical exam (obtain ROM & strength measurements) for both shoulders & complete Physical Exam Form (CRF-02, see Appendix). Physician or delegate completes Part A (ROM) and RA completes Part B (strength).
- Administer baseline questionnaire (CRF-03.1, see Appendix) & review for completeness when done

- Complete enrollment portion of CRF-01 Patient Study Info Overview (electronic CRF)
- Re-confirm subject's willingness to be randomized
- Randomize subject to treatment via REDCap randomization module
    - If subject assigned to operative treatment arm, refer subject to be scheduled for surgery
    - If subject assigned to non-operative treatment arm, refer subject for physical therapy
- Provide subject appropriate PT protocol for assigned treatment arm (to give to treating physical therapist), patient PT brochure, & PT Diary – Part I (CRF-04.1, see Appendix)
- If subject elects to complete PT diary via smartphone, assist subject with installation and setup of MyCap app and provide instructions for use.
- If subject requests more time to consider participation in study, provide contact info for RA and instruct subject to call and schedule return visit to complete enrollment procedures if he/she decides to participate. (RA to follow up with subject approximately one week later to ask if they have made a decision.)

Potential subjects who meet pre-MRI criteria & will not return in person for MRI results or will have a screening MRI:

In Clinic:

- Approach patient and provide information about the trial
- Obtain and document consent from subjects who agree to participate if their MRI confirms eligibility
- Have subject complete authorization form allowing treating physical therapist to share information about PT treatment (see Appendix)
- Administer physical exam (obtain ROM & strength measurements) for both shoulders & complete Physical Exam Form (CRF-02, see Appendix). Physician or delegate completes Part A (ROM) and RA completes Part B (strength).
- Administer baseline questionnaire (CRF-03.1, see Appendix) & review for completeness when done
- Complete the enrollment portion of CRF-01 Patient Study Info Overview (electronic CRF)

- Provide subject patient home exercise PT brochure & PT diary. (PT protocol for appropriate arm to be provided directly to treating physical therapist following randomization.)
- If subject elects to complete PT diary via smartphone, assist subject with installation and setup of MyCap app and provide instructions for use

By phone after MRI:

- Review MRI results with physician (prior to call) to see if subject meets post-MRI eligibility
    - If subject not eligible, inform subject and record as a screen fail
    - If subject eligible, inform subject and re-confirm willingness to participate and be randomized
- Randomize subject to treatment via REDCap randomization module
    - If subject assigned to operative treatment arm, refer subject to be scheduled for surgery
    - If subject assigned to non-operative treatment arm, refer subject for physical therapy

The REDCap Screening Log is to be completed for each subject once they are found to be ineligible, have declined to participate, or are consented for the study. In most cases, screened subjects with pending MRIs should be tracked until MRI results are known and eligibility can be determined.

7.4 Follow-Up

7.4.1 Follow-Up Questionnaires (± 4-6 weeks from follow-up time point)

Subjects enrolled in the study and randomized to treatment will be sent follow-up questionnaires at the following intervals:

- 3 months after randomization
- 6 months after randomization
- 12 months after randomization
- Annually thereafter for years 2-10 (for the sub-study)

Questionnaires can be completed on paper or electronically (via tablet or computer), per the subject's preference.

The target window for completion of follow-up questionnaires is -4 weeks/+6 weeks from the ideal follow-up time point, based on date of randomization. For example, if a subject was randomized to treatment on June 1st, the ideal date for completion of his/her 3-month follow up questionnaire would be Sept 1st (+3 months); the lower window would be August 1st (ideal date minus 1 month) and the upper window would be Oct 14th (ideal date + 6 weeks). Questionnaires will be sent out no earlier than the lower window date.

A concerted, systematic effort will be made to obtain completed questionnaires by the target upper window date (including up to 3 contacts by research staff and a PI call, if needed). However, it will not be considered a protocol deviation if a patient questionnaire is completed after the upper window date. It will only be considered a protocol deviation if a patient questionnaire is not completed at all for a given time point. The date a questionnaire was completed in relation to the ideal date for a given time point will be taken into account during data analysis.

At ALL Follow-Up Time Points:

- Send questionnaires (by mail or electronically) to subjects shortly after their lower window date
- If completed on paper, enter data into REDCap
- If completed electronically, review answers provided in REDCap
- If important data is missing or is ambiguous/contradictory, contact subject to resolve
- If questionnaire not completed/returned within 1-2 weeks, follow up with subject per follow-up procedures specified in MOOP
- If questionnaire not completed/returned for time point after appropriate follow-up procedures are exhausted, record as protocol deviation

At 3-month, 6-month, & 12-month Follow-Up Time Points:

- Review SAE/AE section of questionnaire
    - If subject reports SAE on follow-up questionnaire:
        1) Contact study PI & study coordinator immediately
        2) Complete event report in REDCap

- If subject reports AE on follow up questionnaire:
  1) Complete event report in REDCap
- Monitor changes to assigned treatment (e.g.,crossover) or failure to obtain study treatments, as reported via the Surgery/Trauma Update &/or Treatment sections of the questionnaires:
  - If subject reports information indicating crossover or no treatment:
    1) Verify information provided with patient &/or site research staff
    2) Record as a protocol deviation, if applicable (e.g., if assigned treatment not started within 8 weeks after randomization)
    3) Record as "crossover" in the patient's research record, if applicable (e.g., if crosses over within 6 months after randomization)

At 12-month Follow-up Time Point Only:

- Perform a review of subject's record to see if either surgery and/or physical therapy treatment has been reported by or for subject.
  - If no record of either treatment, verify with patient &/or site research staff, as appropriate
  - If no treatment received within 12 months after randomization, record in research record as "no treatment received" and record as protocol deviation

7.4.2 Physical Therapy Diary (for duration of prescribed physical therapy program)

Subjects who choose to complete their physical therapy diary on paper will typically submit the completed PT diary in two installments, at the time of their 3-month and 6-month follow-up questionnaires, respectively. The first installment (CRF-04.1, see Appendix) will be given to them in clinic at their enrollment visit. The second installment (CRF-04.2, see Appendix) will be sent to them at the time of their 3-month questionnaire. Patients for whom treatment start is delayed &/or who cross over to the other treatment arm may be sent additional copies of the diary, as appropriate. Collection of PT diaries may occur at up to three time points (3, 6, and 12 months) to help ensure that the full duration of their physical therapy program is captured.

Subjects who choose to complete their physical therapy diary via smartphone will enter their information daily until discharged from physical therapy. Verification that this information has been submitted via the app will occur at the time the 3-month, 6-month, and (if needed) 12-month follow-up questionnaires are sent out, with periodic monitoring in between to catch any technical issues.

At 3-Month & 6-Month Follow-Up Time Points Only:

- If subject is completing PT Diary on paper, send subject PT Diary – Part II (CRF-04.2, see Appendix) to replace the PT Diary form that will be submitted with the follow-up questionnaire for this time point or provide additional copies of PT Diary, as appropriate.

At 3-Month, 6-Month, & 12-Month Follow-Up Time Points:

- If the PT Diary is being completed on paper, send collection request to patient, as appropriate
  - If the PT Diary is not returned with the paper questionnaire or is not submitted within 1-2 weeks after the follow-up questionnaire is completed via electronic survey, follow up with subject regarding any outstanding PT treatment data, as appropriate, per procedures specified in the MOOP
  - Once received, enter data into REDCap
- If the PT Diary is completed electronically, review answers provided in REDCap
- If data is missing or is ambiguous/contradictory, contact subject to resolve

A systematic effort will be made to obtain physical therapy compliance data from subjects via the PT diary. However, failure to obtain this information after reasonable follow-up attempts will not be considered a protocol deviation. PT information is also collected from the subject on the follow-up questionnaires during the first year of study participation and from the treating physical therapist(s) via Physical Therapist Report forms.

7.5 Follow-Up Visit & MRI (2-7 years after treatment for sub-study)

On one occasion, approximately 2-7 years after randomization to treatment, subjects may be asked to return for a follow up visit and MRI for the sub-study. During the visit, a physical exam will be performed again to measure range of motion (ROM) and strength in both shoulders.

Subjects may also undergo a research MRI similar to the clinical or screening MRI performed prior to enrollment in the trial.

- Schedule subject to have research MRI
- While subject on site for MRI, perform physical exam (ROM & strength measurements) for both shoulders & complete Physical Exam Form (CRF-02, see Appendix)

8 STUDY PROCEDURES / EVALUATIONS

Study procedures and evaluations will consist of subject- and clinician-reported outcomes and observations as well as imaging to document structural features of the shoulder/rotator cuff.

8.1 Study Procedures/Evaluations

- **Pre-Screening –** review of clinical schedule & available medical records conducted by site coordinator/RA to identify potentially eligible subjects for study

- **Clinical Screening** – Assessment of medical factors related to eligibility for study obtained via interview and observation by physician during clinical examination and recorded on Clinic Screening Form (CRF-00, see Appendix).

- **Screening MRI (if applicable)** – Research MRI of shoulder (without contrast) to determine eligibility for study, at the discretion of the physician. Most subjects will be enrolled in the trial on the basis of a clinical MRI performed as part of their standard of care clinical evaluation. The decision to offer a screening MRI (for research purposes) is a point-of-care decision by the MD.

- **Physical Examination –** Measurement of strength (using a dynamometer) and range of motion (using a visual diagram) of both shoulders in clinic by site Coordinator/RA and the physician or designated assistant. Performed twice – once at enrollment/baseline (for the primary study) and again 2-7 years after treatment (for the sub-study). Recorded on Physical Examination Form (CRF-02, see Appendix).

- **Baseline & Follow-Up Questionnaires -** Patient questionnaires (CRF-03.1, 03.2, 03.3, 03.4, see Appendix) that ask about pain and movement, shoulder symptoms, daily and recreational activities, general and emotional health, and what treatments have been used to help the subject's shoulder. Completed by the subject at baseline and each designated follow-up time point (3mo, 6mo, 12mo, and annually thereafter for years 2-10).

- **Physical Therapy Diary** – Patient-reported record (CRF-04.1, 04.2, see Appendix) of which days the subject attended physical therapy &/or completed their home exercise program during their prescribed rehabilitation program (post-

op or non-op). Collected at 3-month, 6-month, & 12-month follow-up timepoints, as appropriate. Subjects may also opt to complete the diary via smartphone using the installed MyCap app.

- **Surgery Report** – Post-surgery report form (CRF-05, see Appendix) completed by treating surgeon after performing rotator cuff surgery, noting intra-operative observations of tear size, location, and shape; tendon quality and retraction; and cuff repair performed.

- **Physical Therapist Report** – Physical Therapy report form (CRF-06, see Appendix) completed by treating physical therapist, noting start/end dates and frequency of physical therapy attendance by subject and physical therapist's compliance with the prescribed protocol. Completed once at 1 month and again at 3 months after surgery (for subjects in the operative arm) or 3 months after randomization (for subjects in the non-operative arm), respectively. If the subject's treatment start is delayed (or treating physical therapist info is not available) at 1-month time point, a report will also be sent at 6 months.

- **MRI Reading** – MRI Reading Form (CRF-07, see Appendix) completed by study radiologist following an independent review of subject's MRI images and x-rays (if available), noting size, location, and degree of tear; status of biceps tendon, muscle atrophy, tendon retraction and fatty infiltration; as well as presence of arthritis, lesions, and other pathologies. Performed for both initial MRI (at enrollment/baseline) and follow-up MRI (at 2-7 years after treatment).

- **Follow-Up MRI** - Research MRI of shoulder (without contrast) performed at 2-7 years after treatment for sub-study to assess structural changes such as tear size and fatty infiltration of the rotator cuff muscle over time since treatment.

9 ASSESSMENT OF SAFETY

9.1 Specification of Safety Parameters

The operative and non-operative treatments in this trial are standards of usual care. The Adverse Events (AEs) and Serious Adverse Events (SAEs) are therefore those inherent to standard of care treatments. Safety parameters will be recorded in our online data capture system (REDCap). These parameters include the date of occurrence of AE/SAE, a detailed description of the nature of the event, assessment, follow up, and the resolution of the AE/SAE, as appropriate to the event. The safety procedures will be performed for our primary study/trial that ends at one (1) year of follow up and not for the sub-study. Further details are provided in the sections below.

9.1.1 *Unanticipated Problems*

Unanticipated problems include breach of confidentiality or loss of data. Safeguards to prevent this include storage of data in locked file cabinets, use of password protected REDCap databases, use of courier service with tracking for transfer of MRI discs with PHI to the data coordinating center at VUMC, and restricting access to data by authorized study personnel only.

9.1.2 *Adverse Events (AEs)*

AEs for this trial include:

- Post-operative infection
- Post-operative bleeding
- Nerve injury
- Complications due to anesthesia
- Adhesive capsulitis (frozen shoulder)

9.1.3 ***Serious Adverse Events (SAEs)***

SAEs for this trial include:

- Death
- Event requiring hospitalization (in-patient admission) related to treatment

9.2 Time Period and Frequency for Event Assessment and Follow-Up

All reportable events will be recorded in the study record with start dates occurring any time after informed consent is obtained until 7 days (for non-serious AEs) or 30 days (for SAEs) after the last day of study participation for the main study (1 year of follow-up). Subjects will be asked about the occurrence of AE/SAEs on each follow-up questionnaire in the first year.

Events will be followed for outcome information until resolution or stabilization, as appropriate. Unanticipated problems will be recorded in the data collection system throughout the study as discovered.

9.3 Characteristics of an Adverse Event

9.3.1 Relationship to Study Intervention

Study PI will assess relationship to study intervention. To assess relationship of an event to study intervention, the following guidelines will be used:

1. Related (Possible, Probable, Definite)
    a. The event is known to occur with the study intervention.
    b. There is a temporal relationship between the intervention and event onset.
    c. The event abates when the intervention is discontinued.
    d. The event reappears upon a re-challenge with the intervention.
2. Not Related (Unlikely, Not Related)
    a. There is no temporal relationship between the intervention and event onset.
    b. An alternate etiology has been established.

9.3.2 Expectedness of Event

The Study PI will be responsible for determining whether an adverse event is expected or unexpected. An adverse event will be considered unexpected if the nature, severity, or frequency of the event is not consistent with the risk information for the intervention.

9.3.3 Severity of Event

The study PI will determine severity of event. The following scale will be used to grade adverse events:

1. Mild: no intervention required; no impact on activities of daily living (ADL)
2. Moderate: minimal, local, or non-invasive intervention indicated; moderate impact on ADL
3. Severe: significant symptoms requiring invasive intervention; subject seeks medical attention, needs major assistance with ADL

9.4 Reporting Procedures

9.4.1 Related SAEs & Safety Issues

The following will be reported to the IRB, the funding agency (PCORI), and the DSMC within 7 calendar days of the PI becoming aware of the event:

- Any serious adverse event that in the investigator's opinion was unanticipated or unexpected, involved risk to participants or others, and was possibly related to the research procedures
- Any noncompliance with the IRB-approved protocol that increased risk or affected the participant's rights, safety, or welfare.

9.4.2 AEs & Unrelated SAEs

Anticipated AEs or unrelated SAEs will be handled in a less urgent manner but will be reported to the DSMC, IRB, and funding agency (PCORI). All individual AE and Safety reports will be maintained by the investigator and a summary (not individual reports) of all adverse events that have occurred within the last approval period that are associated with the study, whether related or non-related, will be submitted to the IRB at the time of continuing review. These reports will also be submitted to the DSMC and the funding agency (PCORI) during the yearly DSMC meetings.

Adverse events may include a participant's death as a result of a longtime illness (non-related), a breach in confidentiality, or any complaint of a participant unless the risk involved is serious (in which case, the event is reported as an unanticipated problem involving risk to participants or others or as a serious adverse event at the time of occurrence).

9.4.3 Unanticipated Problems

Incidents or events that meet the OHRP criteria for unanticipated problems will be recorded in an event report form. The following information will be included when reporting an adverse event, or any other incident, experience, or outcome as an unanticipated problem to the IRB:

- appropriate identifying information for the research protocol, such as the title, investigator's name, and the IRB project number;
- a detailed description of the adverse event, incident, experience, or outcome;
- an explanation of the basis for determining that the adverse event, incident, experience, or outcome represents an unanticipated problem;
- a description of any changes to the protocol or other corrective actions that have been taken or are proposed in response to the unanticipated problem.

To satisfy the requirement for prompt reporting, unanticipated problems will be reported using the following timeline:

- Unanticipated problems that are serious adverse events will be reported to the IRB and PCORI within 7 calendar days of the investigator becoming aware of the event.
- Any other unanticipated problem will be reported to the IRB, the funding agency (PCORI), and the DSMC in a less urgent manner during annual continuing review and DSMC meetings.

9.5 Halting Rules

While the comparison of treatment arms is novel, the actual therapy in each treatment arms is not. All procedures are standardized, and potential adverse events are well understood. The surgery is an outpatient procedure with very low risk. Nonetheless, a continuous monitoring rule for monitoring SAE has been developed for the DSMC to ensure the SAE rate does not exceed 10%[14].

10 STUDY OVERSIGHT

The study oversight committees will be active for the primary study/trial and dissolve at the end of the primary study. They will not be active for the sub-study.

10.1 Data and Safety Monitoring Committee (DSMC)

In addition to the PI's responsibility for oversight, study oversight will be under the direction of a Data and Safety Monitoring Committee (DSMC) composed of members with expertise in orthopedic surgery, physical therapy, musculoskeletal research, and biostatistics as well a lay community member. The DSMC will operate under the rules of a charter that will be approved at the organizational meeting of the DSMC. The DSMC for this study is convened for the primary study (and not the sub-study).

The DSMC will meet annually, or more frequently at their discretion, to assess safety and efficacy data, study progress, and data integrity for the study. If safety concerns arise, more frequent meetings may be held. At the end of each meeting the DSMC will make a formal recommendation regarding the continuation of the study.

10.2 Scientific Advisory Board

A Scientific Advisory Board of international experts from various disciplines relevant to this trial will meet once per year along with investigators from all sites to review progress, potential trial challenges, discuss methodology issues, and ensure that the study's work reflects evolving clinical science.

10.3 Study Steering Committee

The Steering Committee will be comprised of the study principal investigator (who will serve as Chair), individual site principal investigators, and other members selected for their content expertise as advisors to the Steering Committee. The Steering Committee will meet monthly or every other month to closely monitor trial operations, ensure adequate study accrual, protocol compliance, timely data entry, ensure implementation of the recommendations of the DSMC, and general study integrity.

10.4 Stakeholder Advisory Board

A Stakeholder Advisory Board comprised of a diverse group of individuals with an interest in rotator cuff tears, including patients, their caregivers, and insurance and medical industry representatives, will advise in the development of study materials and the conduct of the study as it relates to study participants. The Stakeholder Advisory Board will meet approximately every other month to review the progress of the trial.

11 CLINICAL SITE MONITORING

Internal clinical site monitoring will be conducted to ensure that the rights of human subjects are protected, that the study is implemented in accordance with the protocol and/or other operating procedures, and that the quality and integrity of study data and data collection methods are maintained. Monitoring for this study will be performed by the study PI and by the executive study coordinator, who will review data and documentation regularly and visit sites as needed. A monitoring report generated from data within the study REDCap databases will be created and used to track and monitor data completion and study activities. These monitoring activities will be performed for the primary study.

11.1 Screening/Enrollment Monitoring Reports

The screening and enrollment data for each site will be reviewed monthly by the executive study coordinator and study PI to ensure that potentially eligible study participants are being screened, approached and consented as appropriate and study-related documents and images are submitted in accordance with the study protocol and Manual of Operating Procedures (MOOP). During the main study, study data analysts will prepare a monthly report of requested information drawn from study records in REDCap.

11.2 Site Monitoring Calls

All sites will participate in regular site calls with the executive study coordinator to review data from Screening, Enrollment & Data reports. Call participants, notes regarding what was discussed, and any actions to be taken will be recorded and reviewed periodically by the study PI. Issues of immediate concern will be brought to the attention of the study PI as they are identified.

11.3 Site Visits

If issues or questions of concern pertaining to site operations arise during the course of the study, the study PI &/or executive study coordinator may visit the site in person to observe how site procedures are being implemented and determine appropriate remedies, if needed.

Sites may also be visited within the first year of recruitment to ensure everything is operating correctly and to address any operational questions or challenges, as deemed appropriate by the study PI.

12 STATISTICAL CONSIDERATIONS

12.1 Study Hypotheses

Aim 1: To compare pain and function in patients undergoing operative versus non-operative treatment of atraumatic rotator cuff tears at 12 months of follow-up.

Hypothesis 1: Patients undergoing operative treatment will have significantly greater improvement in pain reduction and function (measured by SPADI)[12] as compared with those treated non-operatively at 12 months of follow-up.

Aim 2: To assess effects of rotator cuff tear size and age on comparative outcomes (measured by SPADI) in operative versus non-operative treatments for atraumatic rotator cuff tears.

Hypothesis 2a: Surgery will be more effective than non-operative treatment in cuff tears of increasing size.
Hypothesis 2b: Surgery will be more effective than non-operative treatment of rotator cuff tears with decreasing age.

12.2 Final Analysis Plan

Overview: We are planning a trial where patients will be randomly assigned in equal proportion to operative or non-operative care.

Primary Endpoint: The primary outcome measure is SPADI, and the primary endpoint is the change in SPADI at 12 months relative to baseline. SPADI is measured on a scale of zero to 100, with lower scores for better outcomes. SPADI is a reliable and valid measure of shoulder function[15-20].

Secondary Endpoint(s): ASES-SF[13], cross-sectionally and longitudinally over time.

Analysis Population: We define the intent-to-treat (ITT) population as the group of patients randomized to a treatment arm regardless of any other consideration.

12.2.1 Primary Analysis

The primary analysis will be conducted on the intent-to-treat (ITT) population as defined above. The distribution of 12-month change in SPADI, stratified by treatment

arm, will be summarized graphically with histograms, boxplots, and plots of CDFs as appropriate. Our primary analysis for testing the 3 specific hypothesis in section 12.1 will be conducted in the context of a mixed model. That model is,

$$y_{ij} = \beta_0 + \beta_1 I_{\{TRT_i=Op\}} + \beta_2 t_{ij} + \beta_3 I_{\{TRT_i=Op\}} t_{ij} + \beta_4(tearsize_i) + \beta_5(age_i) + \beta_6(I_{\{TRT_i=Op\}} * tearsize_i) + \beta_7(I_{\{TRT_i=Op\}} * age_i) + u_i + e_{ij}$$

where y_{ij} represents the change in SPADI from baseline for participant $i = 1, \dots, n$ at time j= 3, 6, 12 months who was assigned to management arm TRT_i ($I_{\{TRT_i=Op\}} = 1$ if participant i is on the Operative arm and 0 otherwise) . β_0 is the intercept, β_3 is differential change in SPADI score over time due to treatment (i.e., the treatment effect over time; assumed linear here but it will be modeled flexibly as needed), β_4 and β_5 are the effects of tear size and age at the beginning of treatment, and β_6 and β_7 are the tear size and age interactions with treatment that potentially will help identify those patients most likely benefit from therapy. The first 8 terms represent fixed effects. The last 2 terms represent a patient specific random intercept to model the effects of study site and residual error, respectively. The estimate of interest from this longitudinal model is the predicted differential in 12-month SPADI change ($\Delta\Delta SPADI_{12}$; 1st Δ is for treatment change, 2nd Δ is for 12-month change), which is typically estimated as a contrast or difference in predicted 12-month SPADI change.

A directly parallel analysis will also be conducted, where y_{ij} represents SPADI for participant $i = 1, \dots, n$ at time j= 3, 6, 12 months with baseline SPADI as a covariate. In the first model, analyses might lead to a statement such as “Pain and function improved over 12 months in the operative (non-operative) care group 11 points more on average compared to patients treated with non-operative (operative) therapy. In the second model, analyses may lead to a statement such as, “At 12 months, mean SPADI scores were 11 points lower in the non-operative (or operative) group.”

A restricted cubic spline may be fit for continuous covariates to allow for nonlinearity. Further, we will examine different parameterizations of “time” to appropriately capture longitudinal trends (e.g., we might use a time-squared term or let time be categorical). To account for site-to-site variation, the model will include either a fixed effect or random effect for sites (not shown here). The choice between a fixed and random effect often generates debate[21]. While our general preference is to use a random effect, and that is our stated plan here, we realize that with multiple sites the resulting random effect estimates could be unstable due to imbalance in the number

of recruitments, especially if there are pronounced aggregate covariate dissimilarities across sites. In such a case, we will use a fixed effect for site. For completeness, we will report the unadjusted estimates for $\Delta\Delta SPADI_{12}$ that results when Z_i is removed from the model. Note that in a randomized study the purpose of covariate adjustment in a regression model is to obtain unbiased estimates of effects accounting for the enormous variation typical of longitudinal studies.

Heterogeneity of Treatment Effects Analysis:

This analysis, part of the mixed model longitudinal data detailed above, will assess the interaction of age (years at randomization) and tear size (cm) with treatment on $\Delta SPADI_{12}$. The covariate set for the adjusted mixed models is defined in section 12.2.1. Table 1 depicts an example of a binary covariate interaction with treatment with a contrast effect size at the MCID = 10 – 15 – 30 + 45=10. The standard general contrast variance assuming common variance among groups and equal cell size $= \sigma^2[\sum_{i=1}^{k}(\frac{C_i^2}{n_i})$. These elements form the basis for the interaction sample sizes in figure 4.

Table 1. Example Table of Average 12-Month Change in SPADI from Baseline where the Interaction Effect is the MCID

Treatment	Group 1	Group 2
Non-operative	10	15
Operative	30	45

12.2.2 Secondary Analyses

Secondary analyses will be based a more general model denoted,

$$y_{ij} = \beta_0 + \beta_1 I_{\{TRT_i=Op\}} + \beta_2 t_{ij} + \beta_3 I_{\{TRT_i=Op\}} t_{ij} + \gamma Z_i + u_i + e_{ij}$$

where y_{ij} represents the change in ASES-SF (or any other outcome metric) from baseline for participant $i = 1, \ldots, n$ at time j= 3, 6, 12 months who was assigned to management arm TRT_i ($I_{\{TRT_i=Op\}} = 1$ if participant i is on the Operative arm and 0 otherwise) and who has a k-dimensional vector of covariates Z_i (e.g., gender, mental health inventory, fatty infiltration, etc.) with effect parameters γ_k. β_0 is the intercept, and β_3 is differential change in ASES score over time due to treatment (i.e., the treatment effect over time; assumed linear here but it will be modeled flexibly as needed). As in section 12.2.1, the last 2 terms represent a patient specific random intercept and residual error, respectively. The estimate of interest from this longitudinal model is the predicted differential in 12-month ASES-SF

change ($\Delta\Delta$ASES-SF$_{12}$; 1st Δ is for treatment change, 2nd Δ is for 12-month change), which is typically estimated as a contrast or difference in predicted 12 month ASES-SF change.

Similar to the setting described in section 12.2.2 directly parallel analyses will also be conducted, where y_{ij} represents ASES-SF (or other secondary outcomes) for participant $i = 1, \dots, n$ at time j= 3, 6, 12 months with baseline ASES-SF as a covariate. Differential inference between these two sets of models was described above.

12.3 Sample Size Considerations

12.3.1 Primary Aim

A longitudinal mixed model will be used to compare the 12-month change in SPADI between treatment arms. The operating characteristics of this plan depend on the marginal distribution of □SPADI$_{12}$, the assumed true distributional shift, and observed covariate patterns. Figure 3 displays the marginal distribution of □SPADI$_{12}$ for patients by treatment group. These distributions were derived from preliminary data taken from an ongoing prospective, non- randomized cohort of patient with degenerative rotator cuff tears receiving either non-operative or operative intervention. The mean □SPADI$_{12}$ was -16.7 (SD=24.2; n=72) overall. Current estimates for non-operative therapy are -12.3 (SD=21.55; n=45) and -40.6 (SD=19.14; n=27).

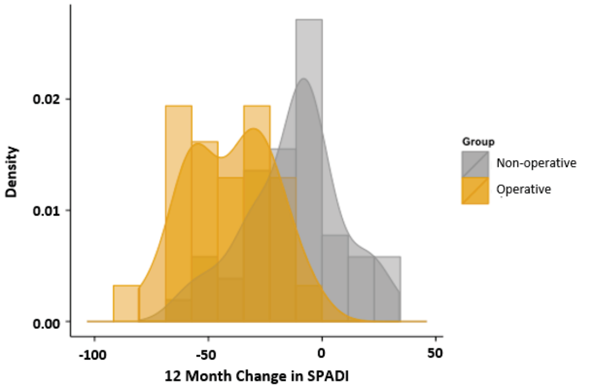

Figure 3. Distributions of 12-month change in SPADI by treatment group.

12.3.2 Effect Size and Alternative Hypotheses

A 10 unit change in SPADI is the smallest clinically meaningful change[14]. In Aim 1 we test the null hypothesis that □SPADI$_{12}$ for patients treated operatively versus non-operatively is equal. As shown below, a sample size of 700 provides outstanding power to detect a 10-unit difference between treatment arms in 12-month SPADI change from baseline.

12.3.3 Sample Size Projections

A sample size of 700 participants (350 per treatment group) provides excellent power even with significant levels of dropout/lost-to-follow-up rate (a two-sided Type I Error of 0.05 was used). Figure 4 illustrates the power for three different tests of $\Delta\Delta SPADI_{12}$. The black line shows the power for a simple t-test of 12-month SPADI change scores between treatment groups. The red line shows the power for a least squared means Wald test of the 12-month SPADI change scores between groups, but adjusted for baseline covariates (mixed model with complete case analysis). The blue line shows the power for the same test from the mixed model, but when multiple imputation is used to properly account for the large number of missing observations in the preliminary data (RVI is 0.53). Here, RVI is the average relative increase (averaged over all coefficients) in variance estimates due to missing values. The largest fraction of missing information (FMI) is 0.412. Interestingly, rather than gaining efficiency, the MI mixed model appears less efficient. Because the MI model properly accounts for variability in the presence of missingness, we can use this model to anticipate a loss of power due to missing data. These curves are based on the estimated standard deviation from the preliminary data: (black) t-test on available data, (red) mixed model on complete case data, (blue) multiply imputed mixed model. The dotted line shows the power to detect the same minimally clinically meaningful change in binary subgroups across treatment groups (e.g., gender by treatment interactions)[22]. The standard deviations for these curves were similarly derived from preliminary data. This approach allows us to carefully leverage preliminary data to anticipate cluster correlation, covariate effects, missing data patterns, and longitudinal correlation patterns. These projections are conservative.

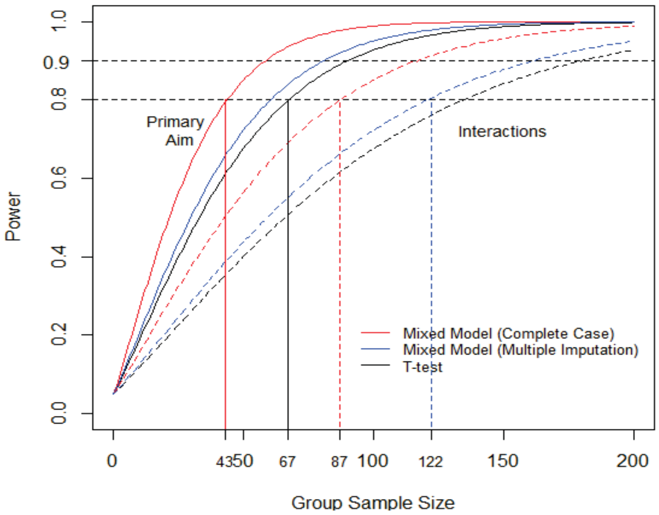

Figure 4. Power curves for primary treatment comparison (solid lines) and interactions (dashed lines) from covariate adjusted

To be clear, power for the adjusted mixed model reaches 90% at 60 patients per group. With the increased variance from imputation, power reaches 90% with 82

patients in each treatment group. For the unadjusted t-test, 90% power is achieved with 91 patients per treatment group. For the subgroup/patient heterogeneity analyses, 80% power to detect a 2-way interaction as small as the MCID is achieved with 122 patients per group (total n=488) for the imputed data set. Consequently, we expect to have excellent power to detect heterogeneous treatment effects, even in the presence of significant missing data (FMI was 0.41). Lastly, we note that in our preliminary data, the intraclass correlation coefficient (ICC) for institution was 0.04 (essentially zero) and largely inconsequential given the longitudinal correlation in SPADI measurements. If necessary, our approach permits the sequestering of site-to-site variability from residual error, increasing power for detecting fixed effects in the data.

12.3.4 Safety Review

A continuous monitoring rule for monitoring SAE has been developed for the DSMC to ensure the SAE rate does not exceed 10%.

12.3.5 Efficacy Review

There is no planned interim analysis of study efficacy outcomes, as all interventions are standard of care.

12.4 Model Assessment and Sensitivity Analyses

Treatment of Crossovers and Missing Data: Although we expect that a significant but manageable number (8% in our observation study) of patients receiving PT only (non-operative treatment) will request surgery (operative treatment), our analytical strategies presented below will also account for the potential of high crossover rates. We order our analyses as follows: First, our primary model will be a strict intent-to-treat analysis, wherein patients who choose to crossover to surgical treatment will have post-surgical outcomes attributed to the treatment arm as randomized. Second, we will treat crossovers as dropouts at the time of crossover and treat the resulting data as missing to be evaluated according to Rubin's classification of missing data and their appropriate analyses[23 24]. Our sample size estimates illustrate that these analyses have good power, even in the presence of significantly high rates of missing/crossover data, and we believe they will provide the best estimates of treatment and covariate effects. Third, we will assign a treatment effect of zero to each crossover patient. This approach assigns an appropriate penalty to the non-

surgical treatment for having failed that patient. This approach will produce appropriately conservative statistical tests and preserve study power.

Assessing Model Fit: The assessment of model fit is indispensable for model development and implementation. Non-linear predictor rescaling, in the form of restricted cubic splines, will be evaluated and models compared using Akaike criteria. Multivariate association and missing data patterns will be evaluated using trellis graphics and clustering algorithms. Such summary analyses may inform sensitivity analyses with respect to modeling assumptions, rescaling of predictors (e.g., restricted cubic splines), and co-linearity[25]. We will repeat these analyses for the secondary outcome (ASES-SF). Along with standard goodness-of- fit and residual analysis, we will perform model validation and calibration using bootstrap methods[26] as discussed in Harrell[25 27].

Sensitivity Analyses: We will not rely only on Gaussian parametric modeling, as our primary mixed effects model will be cross-checked using a proportional odds regression comparing treatment effects after adjusting for baseline SPADI, study site, age, tear size, fatty infiltration, and their interactions[28-30]. We will conduct full information analysis and no levels will be combined. Thus for k unique SPADI change scores, there will be k-1 levels. In addition to the primary contrast of operative versus non-operative treatment and the primary covariates, *a priori* selected independent variables include those noted in Section D.6. In model development, descriptive statistics and graphical displays of outcomes and predictor variables will be examined. Under this category we also note our plans the sequential modeling under MCAR, MAR, and MNAR missing data analyses described below. We will also perform a sensitivity analysis of the time from baseline to a 10 point (MCID) change in SPADI using Cox regression, and the binary outcome of achieving a 10-point change in SPADI, and 30% and 50% improvement in outcome scores (success) versus failure using logistic regression. We will repeat sensitivity analyses for secondary outcomes.

12.5 Sub Study

The analysis for the sub-study will be performed similar to that for Aims 1 and 2 for the main study with additional time points.

13 SOURCE DOCUMENTS & ACCESS TO SOURCE DATA/DOCUMENTS

Study staff will maintain appropriate medical and research records for this study, in compliance with ICH E6, Section 4.9 and regulatory and institutional requirements for the protection of confidentiality of subjects. Study staff will permit authorized representatives of the funder (PCORI) and regulatory agencies to examine (and when required by applicable law, to copy) research records for the purposes of quality assurance reviews, audits, and evaluation of the study safety, progress, and data validity.

13.1 Source Documents

This study will utilize the following source documents:

- Patient medical records, including clinic notes & surgery notes
- X-ray/MRI images
- Case Report Forms (paper &/or electronic)
  - Clinic Screening Form (CRF-00, see Appendix), for patients recruited for the study
  - Patient Study Info Overview Form (CRF-01, see Appendix)
  - Physical Exam Form, Part A & B (CRF-02, see Appendix)
  - Patient Questionnaires:
    - Baseline Questionnaire (CRF-03.1, see Appendix)
    - 3/6 Month Follow-Up Questionnaire (CRF-03.2, see Appendix)
    - 12 Month Follow-Up Questionnaire (CRF-03.3, see Appendix)
    - Annual Follow-Up Questionnaires for sub-study (CRF-03.4, see Appendix)
  - Physical Therapy Diary, Part I & II (CRF-04.1 & 04.2, see Appendix), or MyCap App Physical Therapy Diary (if data reported electronically)
  - Surgery Report Form (CRF-05, see Appendix)
  - Physical Therapist Report Form (CRF-06, see Appendix)
  - MRI Review Form (CRF-07, see Appendix)

CRFs will be considered source documents when they document direct reports by the subject and/or direct observations by clinicians or study personnel. The exception to this are the Clinic Screening Forms for ineligible patients. These forms will be used as worksheets only and, once the information about why the patient was not eligible has been entered into the Screening Log, will not be retained.

13.2 Maintenance of and Access to Source Documents

Individual sites will have access to the medical records and CRFs (paper or electronic versions) only for the subjects recruited from their site. Only coordinating center personnel or personnel authorized to perform follow-up will have electronic access to CRFs for all subjects. Data Access Groups (DAGs) will be created in REDCap to control access to subject data and electronic source documents collected there.

13.2.1 Clinical Screening Forms

- A Clinic Screening Form (CRF-00, see Appendix) will be initiated for potentially eligible patients whose available medical records do not indicate the presence of exclusionary factors. Forms will include basic demographic info but no personally identifiable information.
- The form will be given to the physician prior the clinical exam for use in evaluating the subject's eligibility for the study.
- For subjects determined to be ineligible or who decline to participate, information from the Clinic Screening Form will be entered into the screening database, including the reason for ineligibility &/or non-enrollment. These documents will not be retained.
- For subjects whose MRI results are pending, the partially-completed clinic screening form will be kept in a locked file until final eligibility can be determined.
- For subjects who are eligible for the trial and consent to participate, information from the clinic screening form confirming their eligibility will be entered into the subject database prior to randomization and a copy of the form scanned and uploaded to REDCap. Hard copies will be retained locally on site in the subject's file in a locked cabinet.

13.2.2 Consent Forms

- A copy of the signed consent form will be provided to the subject, either in clinic or by mail, as appropriate.

- If consent was obtained on paper, the original hard copy of the signed consent form will be kept on file at the recruiting site in a locked cabinet (accessible only to the authorized site study personnel)
- A scanned copy of the original signed paper consent form (certified as a true and complete reproduction of the original by site personnel) will be uploaded into the subject's record in REDCap (accessible only to the authorized recruiting site and coordinating center study personnel)
- If consent was obtained via e-Consent, the electronic version completed and signed in REDCap shall be considered the original source document. A printed copy of the completed e-consent form will be provided to the subject.

13.2.3 Paper Case Report Forms

- This includes case report forms originally completed on paper (with the exception of Clinic Screening Forms for ineligible patients).
- The original hard copy will be kept on file in a locked cabinet at the site where it was originally completed/collected (accessible only to authorized local site study personnel)
- A scanned copy (certified as a true and complete reproduction of the original by site personnel) will be uploaded into the subject's record in REDCap (accessible only to authorized recruiting site and coordinating center study personnel)

13.2.4 Electronic Case Report Forms

- This includes any case report forms completed via electronic survey or entered directly into MyCap or REDCap by subjects, clinicians, or study personnel.
- Electronic data originally captured via direct data entry by the reporting source will be stored in the subject's REDCap record.
- If necessary for data review or verification purposes, a copy of the corresponding electronic form's data may be downloaded and printed or saved to a secure server (accessible only to authorized study personnel). Any printed copies will be stored in the subject's file in a locked cabinet. Downloaded or printed forms may be deleted or properly destroyed if no longer needed for review or verification purposes.

13.2.5 MRI / X-Ray Images

- This includes images available directly within the electronic medical record or electronic copies of these images.

- Copies of the enrolled subject's MRI/X-Ray images used for the purpose of determining/verifying eligibility at recruiting sites will be burned to CD disc, marked with their study ID and mailed via FedEx or other tracked mail service to the coordinating center. Discs received will be secured in a locked cabinet.

- MRI / X-Ray images will be de-identified using DicomCleaner software and saved to a secure share drive on Vanderbilt's server. Access to the folder containing these images will be restricted to the study PI, coordinating center personnel, and the study radiologist.

- The de-identified images will be reviewed by the study radiologist in completing and documenting the MRI review.

14 QUALITY CONTROL AND QUALITY ASSURANCE

14.1 Electronic Data Capture (REDCap)

Study data will be collected and managed using REDCap[31] electronic data capture tools[32]. REDCap (Research Electronic Data Capture) is a secure, web-based application designed to support data capture for research studies, providing 1) an intuitive interface for validated data entry; 2) audit trails for tracking data manipulation and export procedures; 3) automated export procedures for seamless data downloads to common statistical packages; and 4) procedures for importing data from external sources. The system uses a meta-data approach to build the user interface in an automated fashion. Thus, the study team defines the data elements as would occur for any typical trial. The data dictionary is then submitted to the system in a standardized format, and a web-based case report form is created in a fraction of the time and cost of traditional electronic systems.

14.2 Data Security

Components of the REDCap software have 21CFR11 compliance. REDCap servers are housed in a local data center at Vanderbilt, and all web-based information transmission is encrypted. REDCap was developed specifically around HIPAA-Security guidelines and is the preferred medium by Vanderbilt's Privacy Office and Institutional Review Board.

14.3 Data Entry

The Data Coordinating Center will use REDCap for data entry, data validation, audit trails, export procedures, and downloading to statistical packages. Data will download into R or SAS (or other appropriate statistical software) for data cleaning and data quality checks. An initial check of data completeness is done via REDCap's logic and range checking that occurs at data entry and is customizable. Before the data is entered into the database, the user is prompted to revise data if the initial check finds problems with the data, key fields are skipped, values are missing or out of range, etc.

14.4 Staff Training and Tracking

The best approach to ensuring good quality data is to provide training and support to those individuals who collect and enter data. The Data Coordinating Center will work closely with the RAs at each site to ensure they understand and can perform their duties to the best of their abilities. A "kick-off" meeting is planned prior to start of enrollment at

the coordinating center. We will go over basic study procedures as they relate to the MDs, physical therapists, and the RAs, respectively.

Additionally, detailed, written step-by-step instructions will be provided for tasks to be performed by site personnel, and live training sessions via teleconference will be scheduled for recruiting MDs, site Lead Physical Therapists, and site Coordinators/RAs prior to the start of enrollment at each site.

Regular site calls will be led by the study coordinator to discuss recruitment and any data/study protocol related issues. Coordinators / RAs and study staff will complete required training in the protection of human subjects and Good Clinical Practice (GCP) requirements.

A separate training database in REDCap will be utilized to track the training of personnel from all sites on the study protocol, operating procedures, study amendments, as well as to document completion of required human subjects protection and Good Clinical Practice (GCP) training.

14.5 Standard Operating Procedures (SOPs)

Standard Operating Procedures (SOPs) will be developed and included in the Manual of Operating Procedures (MOOP) detailing how data should be entered and will be evaluated for accuracy in relation to source documents.

14.6 Data Review

Coordinating center personnel will review data as it is entered and note any incomplete, ambiguous, or conflicting items. The study participant, clinician or study staff member who submitted the data will be contacted to provide or clarify any data that is missing or in question. Surveys completed electronically &/or direct data entry performed by the subject, clinician, or study personnel will be reviewed for completeness and consistency by study staff, and any items requiring attention will be similarly resolved. Once the data review is complete, the record will be locked by coordinating center personnel to prevent further changes.

14.7 Data Validation and Query Management

Data validation and query management procedures will identify suspicious data through the application of validation rules, generate requests for data review by study sites, and monitor the resolution of these requests. This entails performing more complex checks

on the data than done at web entry (e.g., cross-form data validation and querying). Data that fail validations will be flagged with a status of “suspicious,” reviewed by a data manager or designee and clarified by consulting the appropriate source documents.

14.8 Quality Review and Data Auditing

Over the course of the primary study, the Data Coordinating Center will randomly select at least 10% of each site’s electronic data capture forms for independent validation and review. If the percentage of errors found on any audit is greater than 10%, a more thorough audit will be performed. Audits revealing substantial data quality issues are cause for suspending site enrollment until the current data is thoroughly cleaned and validated. Decisions on suspension of site enrollment will be made by the study PI. The study PI and Study Principal Statistician will be responsible for addressing quality assurance issues (correcting procedures that are not in compliance with protocol) and quality control issues (correcting errors in the data entry).

15 ETHICS/PROTECTION OF HUMAN SUBJECTS

15.1 Ethical Standard

The investigator will ensure that this study is conducted in full conformity with the principles set forth in The Belmont Report: Ethical Principles and Guidelines for the Protection of Human Subjects of Research, as drafted by the US National Commission for the Protection of Human Subjects of Biomedical and Behavioral Research (April 18, 1979) and codified in 45 CFR Part 46 and/or the ICH E6.

15.2 Institutional Review Board

The protocol, informed consent form(s), recruitment materials, and all subject materials will be submitted to the IRB for review and approval. Approval of both the protocol and the consent form must be obtained before any subject is enrolled. Any amendment to the protocol will require review and approval by the IRB before the changes are implemented in the study.

Review and approval of the protocol and supporting materials for this multi-site study will be conducted by a single, central IRB centered at Vanderbilt. The relying IRBs of participating sites will ensure compliance with local laws, institutional policies, and federal regulations at their respective sites.

15.3 Informed Consent Process

Informed consent is a process that is initiated prior to the individual agreeing to participate in the study and continues throughout study participation. Extensive discussion of risks and possible benefits of study participation will be provided to subjects and their families, if applicable. A consent form describing in detail the study procedures and risks will be given to the subject. Consent forms will be IRB-approved, and the subject is required to read and review the document or have the document read to him or her. The investigator or designee will explain the research study to the subject and answer any questions that may arise. The subject will sign the informed consent document prior to any study-related assessments or procedures. Subjects will be given the opportunity to discuss the study with their surrogates or think about it prior to agreeing to participate. They may withdraw consent at any time throughout the course of the study. A copy of the signed informed consent document will be given to subjects for their records. The rights and welfare of the subjects will be protected by emphasizing to them that the quality of their clinical care will not be adversely affected if they decline to participate in this study. The consent process will be documented in the research record.

Each participating institution will be provided with a model consent form. Each institution may revise or add information to comply with institutional consent policies &/or local laws, but may not remove procedural or risk content from the model consent form.

15.4 Exclusion of Women, Minorities, and Children (Special Populations)

Individuals of any gender or racial/ethnic group within the specified age range may participate in this study. Pregnant women are excluded from this study, due to pregnancy being a contraindication to MRI. Non-English speakers are also excluded from this study, as the standardized questionnaires by which primary and secondary outcomes will be measured are only validated in English.

15.5 Subject Confidentiality

Subject confidentiality is strictly held in trust by the investigators, study staff, and the funder(s) and their agents. This confidentiality is extended to cover medical imaging and tests in addition to any study information relating to subjects.

The study protocol, documentation, data, and all other information generated will be held in strict confidence. No information concerning the research study or the data will be released to any unauthorized third party.

The study monitor, auditor(s), authorized representatives of the IRB, the funder/sponsor or regulatory agencies may inspect all study documents and records required to be maintained by the investigators, including, but not limited to, medical records (office, clinic, or hospital) for the study subjects. The clinical study sites will permit access to such records.

16 DATA HANDLING AND RECORD KEEPING

The investigators are responsible for ensuring the accuracy, completeness, legibility, and timeliness of the data reported. All source documents should be completed in a neat, legible manner to ensure accurate interpretation of data. Any corrections to paper or electronic CRFs should be documented in accordance with specified guidelines and explanatory notes added as appropriate. The investigators will maintain adequate case histories of study subjects, including accurate case report forms (CRFs), and source documentation.

16.1 Data Management Responsibilities

Data collection and accurate documentation are the responsibility of the study staff under the supervision of the investigator. All source documents and laboratory reports must be reviewed by the study team and data entry staff who will ensure that they are accurate and complete. Scanned copies of all original documents and paper CRFs will be uploaded directly into REDCap and original paper documents and forms stored securely on site at the location they were completed or received. Unanticipated problems and adverse events must be reviewed by the investigator or designee.

16.1.1 Recruiting Sites

Recruiting site personnel are responsible for:

- documenting screening of potentially eligible subjects
- documenting subject eligibility and consent
- administering baseline questionnaires and physical examinations for consented subjects, and assisting with follow-ups if requested by the coordinating center
- entering basic required data into REDCap (Patient Info Overview, Eligibility, etc.) for consented subjects
- randomizing subjects to treatment via REDCap
- sending subjects' MRI/x-ray imaging to the coordinating center
- ensuring completion and submission of surgery reports for subjects randomized to operative treatment, and

- reporting AEs, SAEs, and unanticipated problems to the coordinating center upon becoming aware of them

16.1.2 Coordinating Center (VUMC)

Coordinating center personnel are responsible for:

- ensuring that copies of original paper documents and CRFs are uploaded to REDCap by recruiting sites
- performing primary and double data entry of most CRFs
- collecting and de-identifying subjects' MRI / x-ray imaging and coordinating MRI review by study radiologist
- administering all subject follow-up questionnaires
- collecting subject physical therapy diaries
- sending & collecting physical therapist reports
- ensuring appropriate recording, reporting, and action for AEs, SAEs, and unanticipated problems

16.2 Data Capture Methods

The study will utilize both paper and electronic data capture methods, as subjects will have the option to complete questionnaires and diaries either on paper or online. All research data will be centrally collected and stored in a REDCap electronic data capture system. Some of the data will be entered directly into the system by the subject or reporting clinician or staff member via electronic means (tablet, computer, or smartphone), while other data will be manually entered by study staff from paper CRFs. Access to the REDCap system is password protected and data access groups (DAGs) will be utilized to limit access as appropriate by site and/or role.

Collection of screening, enrollment, and baseline data will be distributed across recruiting sites with limited data entry required locally. Most data entry will be performed centrally at the coordinating center to ensure consistency of data entry. Collection of subject follow up data via questionnaires and physical therapy diaries, as well as of data from physical therapist reports and imaging reviews, will be performed centrally by the coordinating center.

Collection and processing of enrollment, baseline, and follow up data will be ongoing throughout the course of the study, relative to subject randomization dates and corresponding follow up time points. Collection of treatment information (surgery and physical therapy reports) will occur at designated time points, relative to subject randomization and date of surgery. Collection and processing of MRI / x-ray review data will be batched, occurring on an ongoing basis during subject enrollment and follow-up.

16.3 Types of Data

The types of data to be collected include: screening information (age, demographic data, eligibility, etc.); MRI/x-ray imaging; questionnaire responses from subjects; physical exam measurements; laboratory reports (re: surgery & physical therapy) from treating clinicians and reviewing radiologist; as well as safety data.

16.4 Schedule and Content of Reports

16.4.1 Monthly Screening, Enrollment and Data Report (every month)

Study data analysts will prepare a report each month detailing the screening, enrollment, crossover and dropout rates for all sites as well as information on quality control items such as missing and suspicious data. This report will be reviewed with sites during regular site calls and by the Steering Committee during monthly calls so that issues of concern can be identified and addressed in a timely manner.

16.4.2 Interim Progress Reports to PCORI (every 6 months)

Every 6 months, an Interim Progress Report will be submitted to PCORI providing updates on milestones, key personnel, financial status, accomplishments & challenges, and publications.

16.4.3 Data Monitoring Reports to DSMC (every 6-12 months)

Prior to each meeting of the DSMC, study data analysts will prepare a Data Monitoring Report focused on the conduct of the study, including pooled enrollment and dropout rates, timeliness (compliance) of data submission, eligibility rates, reason(s) for ineligibility, baseline data, landmark frequency data, and AEs/SAEs.

16.4.4 Final Progress Report to PCORI (at conclusion of study)

At the conclusion of the study, a Final Progress Report will be submitted to PCORI which contains the following:

- overview of study findings and impact,
- milestones update,
- recruitment, enrollment, and retention update,
- accomplishments and challenges,
- engagement report,
- financial status update,
- key personnel effort update,
- publications update,
- data sharing,
- future directions,
- progress statement for public use

16.5 Study Records Retention

Study records (paper and electronic) will be maintained for at least three years from the completion of the study and the PCORI contract term date.

16.6 Protocol Deviations and Violations

Broadly speaking, a protocol deviation is any noncompliance with the clinical study protocol and may be on the part of the subject, the investigator, or study staff. As a result of deviations, corrective actions are to be developed by the study staff and implemented promptly.

All deviations from the protocol must be addressed in study subject source documents, documented in the study record, and reported to the IRB and funding agency, according to their requirements.

16.6.1 Protocol Deviations

A protocol deviation an incident involving noncompliance with the protocol, but one that typically does not have a significant effect on the subject's rights, safety, welfare, and/or the integrity of the resultant data. Deviations may result from the action of the participant, investigator, or staff. (VUMC IRB Handbook, 2016)

Examples of protocol deviations include (not an exhaustive list):

- Signed copy of consent form not given to subject during informed consent process
- Subject does not begin treatment within 2 months following randomization
- Subject does not have assigned treatment within 6 months after randomization
- Subject does not pursue either treatment (surgery or physical therapy) within 12 months after randomization
- Follow-up questionnaire not sent to subject for designated time point

16.6.2 Protocol Violations

A protocol violation is an accidental or unintentional change to the IRB-approved protocol procedures without prior sponsor and IRB approval. Violations, as opposed to simply deviations, generally affect the subject's rights, safety, welfare, and/or the integrity of the resultant data. (VUMC IRB Handbook, 2016)

Examples of protocol violations include (not an exhaustive list):

- Informed consent obtained after the initiation of study procedures
- Failure to obtain informed consent
- Failure to report a Serious Adverse Event

17 PUBLICATION / DATA SHARING POLICY

This study will comply with the *NIH Public Access Policy*, which ensures that the public has access to the published results. It requires scientists to submit final peer-reviewed journal manuscripts that arise from NIH funds to the digital archive PubMed Central upon acceptance for publication. PCORI has similar requirements and the preparation of this trial was funded by NIH.

The International Committee of Medical Journal Editors (ICMJE) member journals have adopted a clinical trials registration policy as a condition for publication. The ARC trial will be registered with *ClinicalTrials.gov* prior to enrollment of the first participant.

Criteria for authorship on publications resulting from this trial are described in the MOOP. An executive committee chaired by the PI will be responsible for developing publication procedures and resolving authorship issues.

The PI supports sharing of research resources to promote advances towards optimizing outcomes in patients with rotator cuff tears. De-identified datasets will be shared with scientists interested in the topic after approval from the IRB and the PI is obtained. Requests for data must be submitted in writing and must clearly state the intended use and the length of time for data analysis. Each request will be reviewed and will need to be approved by the PI. The PI reserves the right to deny data requests.

17.1 Publication Guidelines

The ARC trial will have its primary publication on the effectiveness of surgery versus physical therapy for atraumatic rotator cuff tears. For this/these publications, the participating MDs should have randomized at least 15 patients for the trial to be considered for co-authorship. They would also need to satisfy other criteria set forth by each of the journals for authorship. Given the large number of MDs participating in the trial, these names will be consolidated as “The ARC Trial Team.” The final decision for granting authorship would be based on relative contributions of team members and will be determined by the study PI.

18 LITERATURE REFERENCES

1. CDC/NCHS. National Ambulatory Medical Care Survey: 2010 Summary Tables. 2012. *http://www.cdc.gov/nchs/ahcd/web_tables.htm#2010* (accessed Jan 25, 2013).
2. Colvin AC, Egorova N, Harrison AK, et al. National trends in rotator cuff repair. The Journal of bone and joint surgery American volume 2012;**94**(3):227-33.
3. Jain NB, Higgins LD, Losina E, et al. Epidemiology of musculoskeletal upper extremity ambulatory surgery in the United States. BMC musculoskeletal disorders 2014;**15**(1):4.
4. Varkey DT, Patterson BM, Creighton RA, et al. Initial medical management of rotator cuff tears: a demographic analysis of surgical and nonsurgical treatment in the United States Medicare population. Journal of shoulder and elbow surgery / American Shoulder and Elbow Surgeons [et al] 2016 (Epub).
5. Pedowitz RA, Yamaguchi K, Ahmad CS, et al. American Academy of Orthopaedic Surgeons Clinical Practice Guideline on: optimizing the management of rotator cuff problems. The Journal of bone and joint surgery American volume 2012;**94**(2):163-7.
6. Agency for Healthcare Research and Quality (AHRQ). Comparative effectiveness of non-operative and operative treatments for rotator cuff tears. Effective Health Care 2008. (accessed 1/25/2009).
7. Coghlan JA, Buchbinder R, Green S, et al. Surgery for rotator cuff disease. Cochrane Database Syst Rev 2008(1):CD005619.
8. Green S, Buchbinder R, Hetrick S. Physiotherapy interventions for shoulder pain. Cochrane Database Syst Rev 2003(2):CD004258.
9. Matsen FA, 3rd. Clinical practice. Rotator-cuff failure. The New England journal of medicine 2008;**358**(20):2138-47.
10. Williams GR, Jr., Rockwood CA, Jr., Bigliani LU, et al. Rotator cuff tears: why do we repair them? The Journal of bone and joint surgery American volume 2004;**86-A**(12):2764-76.
11. Mackey DC, Lui LY, Cawthon PM, et al. High-trauma fractures and low bone mineral density in older women and men. JAMA 2007;**298**(20):2381-8.
12. Roach KE, Budiman-Mak E, Songsiridej N, et al. Development of a shoulder pain and disability index. Arthritis care and research : the official journal of the Arthritis Health Professions Association 1991;**4**(4):143-9.
13. Richards RR, An KN, Bigliani LU, et al. A standardized method for the assessment of shoulder function. J Shoulder Elbow Surg 1994;**3**(6):347-52.
14. Ivanova A, Qaqish BF, Schell MJ. Continuous toxicity monitoring in phase II trials in oncology. Biometrics 2005;**61**(2):540-5.
15. Beaton DE, Richards RR. Measuring function of the shoulder. A cross-sectional comparison of five questionnaires. J Bone Joint Surg Am 1996;**78**(6):882-90.

16. Christie A, Hagen KB, Mowinckel P, et al. Methodological properties of six shoulder disability measures in patients with rheumatic diseases referred for shoulder surgery. J Shoulder Elbow Surg 2009;**18**(1):89-95.
17. Ekeberg OM, Bautz-Holter E, Tveita EK, et al. Agreement, reliability and validity in 3 shoulder questionnaires in patients with rotator cuff disease. BMC Musculoskelet Disord 2008;**9**:68.
18. MacDermid JC, Ramos J, Drosdowech D, et al. The impact of rotator cuff pathology on isometric and isokinetic strength, function, and quality of life. Journal of shoulder and elbow surgery / American Shoulder and Elbow Surgeons [et al] 2004;**13**(6):593-8.
19. Paul A, Lewis M, Shadforth MF, et al. A comparison of four shoulder-specific questionnaires in primary care. Ann Rheum Dis 2004;**63**(10):1293-9.
20. Williams JW, Jr., Holleman DR, Jr., Simel DL. Measuring shoulder function with the Shoulder Pain and Disability Index. J Rheumatol 1995;**22**(4):727-32.
21. Hijioka A, Suzuki K, Nakamura T, et al. Degenerative change and rotator cuff tears. An anatomical study in 160 shoulders of 80 cadavers. Arch Orthop Trauma Surg 1993;**112**(2):61-4.
22. Lachenbruch PA. A note on sample size computation for testing interactions. Statistics in medicine 1988;**7**(4):467-9.
23. Hogan JW, Roy J, Korkontzelou C. Handling drop-out in longitudinal studies. Statistics in medicine 2004;**23**(9):1455-97.
24. Marshall A, Altman DG, Holder RL, et al. Combining estimates of interest in prognostic modelling studies after multiple imputation: current practice and guidelines. BMC Med Res Methodol 2009;**9**:57.
25. Harrell FE, Jr., Lee KL, Mark DB. Multivariable prognostic models: issues in developing models, evaluating assumptions and adequacy, and measuring and reducing errors. Statistics in medicine 1996;**15**(4):361-87.
26. Efron, B., Tibshirani, R.J. 1993. An introduction to the bootstrap. Monographs on statistics and applied probability, Chapman & Hall, London.
27. Harrell FE, Jr., Margolis PA, Gove S, et al. Development of a clinical prediction model for an ordinal outcome: the World Health Organization Multicentre Study of Clinical Signs and Etiological agents of Pneumonia, Sepsis and Meningitis in Young Infants. WHO/ARI Young Infant Multicentre Study Group. Statistics in medicine 1998;**17**(8):909-44.
28. Anderson, J.A., Philips, P.R. 1981. Regression, discrimination and measurement models for ordered categorical variables. Applied Statistics, 30, 22-31.
29. Anderson, J.A. 1984. Regression and ordered categorical variables. Journal of the Royal Statistical Society, Series B, 46:1-30.
30. Agresti A. A survey of models for repeated ordered categorical response data. Statistics in medicine 1989;**8**(10):1209-24.
31. REDCap (Research Electronic Data Capture). *http://project-redcap.org/* (Accessed 10/13/2013).

32. Harris PA, Taylor R, Thielke R, et al. Research electronic data capture (REDCap)--a metadata-driven methodology and workflow process for providing translational research informatics support. Journal of biomedical informatics 2009;**42**(2):377-81.

SUPPLEMENTAL MATERIALS

These documents are relevant to the protocol, but they are not considered part of the protocol. They are stored and modified separately. As such, modifications to these documents do not require IRB amendments.

- Study/Site Roster
- Manual of Operating Procedures (MOOP)
- Standard Operating Procedures (SOP) for Recruiters
- Standard Operating Procedures (SOP) for Site Lead PTs
- Instructions / PT Report Form Completion Guide for Treating Physical Therapists
- Surgical Protocol
- Post-operative Rehabilitation Protocol
- Non-operative Rehabilitation Protocol
- Physical Therapist Cheatsheet
- MRI Protocol
- Dynamometry Protocol
- ARC Study Physician FAQs
- ARC Study Physical Therapist FAQs
- ARC Study Personnel FAQs
- Data and Safety Monitoring Committee (DSMC) Charter

APPENDICES

These documents are officially affiliated with the protocol and will be submitted to the IRB as a part of the protocol. As such, changes to these items require an IRB amendment.

Appendix A:	Schedule of Events
Appendix B:	Inclusion/Exclusion Criteria Reference Card (for MDs, Coordinators/RAs)
Appendix C:	Promotional Materials:<br>• Patient Brochure (tri-fold)<br>• Clinic Patient Poster<br>• Clinic Staff Poster
Appendix D:	Recruitment Scripts & Materials:<br>• Pre-Visit Call Sample Script<br>• Pre-Visit Cover Letter<br>• MD Sample Script (for discussing trial with patients)<br>• Recruitment & Consent Sample Script (for Coordinators/RAs)<br>• Recruitment Video Slides & Script<br>• Consent Video Slides & Script<br>• Follow-up Sample Script for Patient Who Need More Time to Decide
Appendix E:	Patient Information & Materials:<br>• ARC Study Patient Fact Sheet Template (for subjects)<br>• FAQs for Study Participants<br>• Patient Physical Therapy Brochure<br>• MyCap App Instructions (for subjects)<br>• Study folder (for subjects)<br>• Study Pen<br>• Study Magnet
Appendix F:	Consent Documents:<br>• Consent Form Template<br>• Info Release Form (to go to treating Physical Therapist)

Appendix G:	Case Report Forms:<br>• Clinic Screening Form (CRF-00)<br>• Patient Study Info Overview (CRF-01)<br>• Physical Exam Form – Part A & B (CRF-02)<br>• Baseline Questionnaire (CRF-03.1)<br>• 3-/6-Month Follow Up Questionnaire (CRF-03.2) – Left & Right versions<br>• 12-Month Follow Up Questionnaire (CRF-03.3) – Left & Right versions<br>• Annual Follow Up Questionnaire, years 2-10 for sub-study (CRF-03.4) – Left & Right versions<br>• Physical Therapy Diary, Part I & II (CRF-04.1 & 04.2)<br>• Surgery Report Form (CRF-05)<br>• Physical Therapist Report Form (CRF-06)<br>• MRI Review Form (CRF-07)
Appendix H:	Correspondence:<br>• PT Study Intro Letters (for inside PT packet)<br>• Questionnaire Cover Letters<br>• Termination Letter<br>• Study Completion Letter

APPENDIX A: SCHEDULE OF EVENTS

	Main Study					Sub-Study									
**Procedures**	**Screening**	**Baseline**	**3 Month Follow Up**	**6 Month Follow Up**	**12 Month Follow Up**	**2 Year Follow Up**	**3 Year Follow Up**	**4 Year Follow Up**	**5 Year Follow Up**	**6 Year Follow Up**	**7 Year Follow Up**	**8 Year Follow Up**	**9 Year Follow Up**	**10 Year Follow Up**	**Follow Up Visit (2-7 years)**
Physical Exam (ROM & strength)		X													X
PT Diary			X	X	X*										
Questionnaires		X	X	X	X	X	X	X	X	X	X	X	X	X	X
Research MRI	X*														X

* If applicable
