# Supplementary material for: Operative vs Nonoperative Treatment for Atraumatic Rotator Cuff Tears: A Trial Protocol for the Arthroscopic Rotator Cuff Pragmatic Randomized Clinical Trial
Source: JAMA Netw Open. 2019 Aug 9;2(8):e199050. doi: 10.1001/jamanetworkopen.2019.9050 (PMC6692688; doi:10.1001/jamanetworkopen.2019.9050)
Supplement: Supplement 2. — eAppendix 1. Nonoperative Rehabilitation Protocol eAppendix 2. ARC Surgical Protocol eAppendix 3. Postoperative Rehabilitation Protocol [file jamanetwopen-2-e199050-s002.pdf]

Supplementary Online Content

Jain NB, Ayers GD, Koudelková H, et al; ARC Trial Group. Operative vs nonoperative treatment for atraumatic rotator cuff tears: a trial protocol for the Arthroscopic Rotator Cuff pragmatic randomized clinical trial. *JAMA Netw Open*. 2019;2(8):e199050. doi:10.1001/jamanetworkopen.2019.9050

eAppendix 1. Nonoperative Rehabilitation Protocol

eAppendix 2. ARC Surgical Protocol

eAppendix 3. Postoperative Rehabilitation Protocol

This supplementary material has been provided by the authors to give readers additional information about their work.

© 2019 Jain NB et al. *JAMA Network Open.*

eAppendix 1. Nonoperative Rehabilitation Protocol

Reproduced with permission of Vanderbilt University Medical Center

© 2019 Jain NB et al. *JAMA Network Open.*

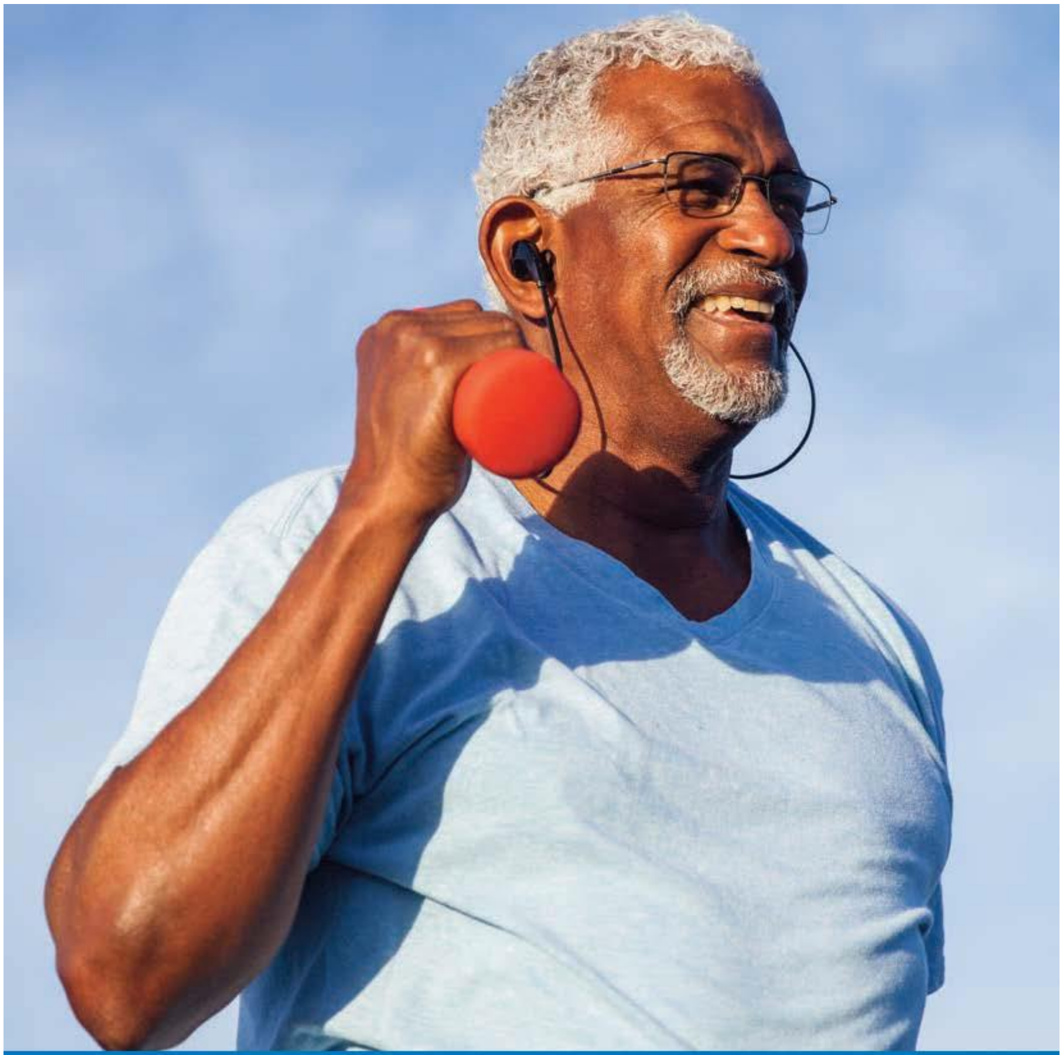

ARC
ARTHROSCOPIC ROTATOR CUFF TRIAL

NONOPERATIVE REHABILITATION PROTOCOL

ShoulderStudy.com

© 2019 Jain NB et al. *JAMA Network Open.*

Arthroscopic Rotator Cuff (ARC) Clinical Trial

NONOPERATIVE REHABILITATION PROTOCOL

TABLE OF CONTENTS

INTRODUCTION 1

GENERAL INSTRUCTIONS 2

PHASE ONE 3
Passive Motion & Scapular Exercises

PHASE TWO 5
Active Assisted Motion, Shoulder Stretches, Rotator Cuff Strengthening & Scapular Stabilization

PHASE THREE 11
Advanced Scapular Stabilization, Rotator Cuff Strengthening & Rhythmic Stabilization

Study Investigators & Partner Sites 14

© 2019 Jain NB et al. *JAMA Network Open.*

Arthroscopic Rotator Cuff (ARC) Clinical Trial

NONOPERATIVE REHABILITATION PROTOCOL

NONOPERATIVE GUIDELINES

Do not add or skip any part of this program.
If you have questions or concerns, please contact
the Lead Physical Therapist for your site below.

You may also contact the trial's Lead Physical Therapists at Vanderbilt:

Brian Richardson: brian.richardson@vanderbilt.edu
Rebecca Dickinson: rebecca.dickinson@vanderbilt.edu

© 2019 Jain NB et al. *JAMA Network Open.*

GENERAL INSTRUCTIONS

- The patient should work with the physical therapist approximately 1-2x/week for 6-12 weeks. There is no required minimum number of visits per week.
- The patient should perform a home exercise program (HEP) as prescribed by the physical therapist approximately 30 minutes/day, 2-4x/week.
- The combined total of physical therapy visits & HEP (in any combination) should equal 4+x/week.

Modalities

Patients are encouraged to use cryotherapy after exercise or any time the shoulder is painful.

Each phase should include:

- Physical therapy (PT) intervention as indicated by treating physical therapist's plan of care within guidelines of this study protocol
- Perform Home Exercise Program (HEP)

Progression:

- For each exercise, begin with 1 set (up to 10 repetitions per set) and progress to 3 sets (up to 10 repetitions per set). The number of sets/reps given should be based on good quality movement.
- The non-operative guidelines should be advanced per progression criteria.
- If the patient has met all of the criteria, then the patient should be progressed to the next phase.
- Patients will be evaluated for various impairments. Once these impairments have been identified, certain exercises will be given to address those impairments. If there is no impairment identified, it is not necessary to perform the exercise which addresses that particular impairment.

© 2019 Jain NB et al. *JAMA Network Open.*

PHASE ONE: Passive Motion & Scapular Exercises

GOALS OF PHASE ONE:

- Restore PROM to the shoulder
- Minimize pain and edema

Normalize ROM of the shoulder by performing manual techniques. Manual techniques may include: joint mobilizations, soft tissue mobilizations, and active release techniques. ROM limitations to both the cervical spine and thoracic spine should be addressed as well. Passive range of motion (PROM) requires the therapist to put the arm through a comfortable range of motion while the patient is supine. Motions include flexion, abduction, external rotation, and internal rotation. Manual therapy, pendulums, and scapular retraction should be done at each therapy visit. Ice may be used as needed.

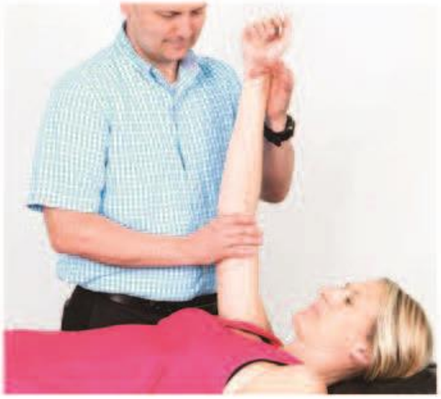

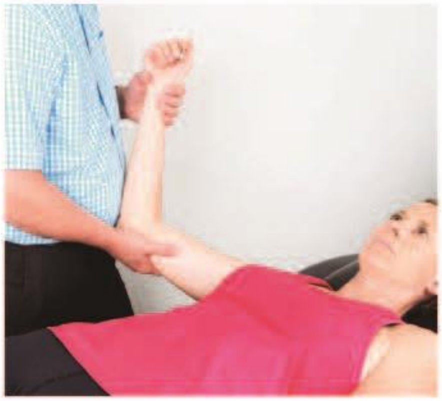

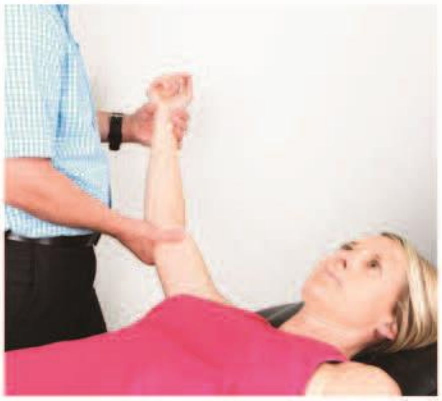

PROM: The therapist should move the arm while the patent remains relaxed.

© 2019 Jain NB et al. *JAMA Network Open.*

PHASE ONE

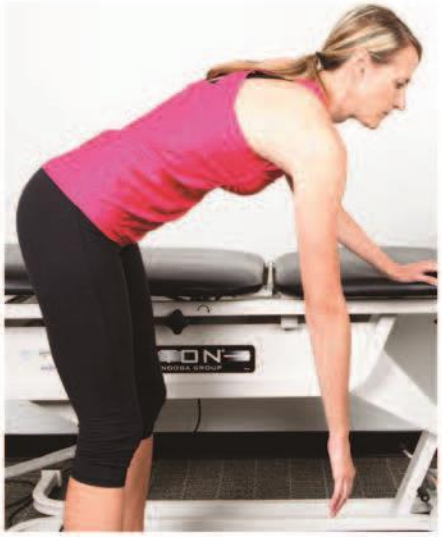

PENDULUM EXERCISES
Clockwise/Counterclockwise, forward back and side/side. Keep the arm relaxed and move at the hips and trunk.

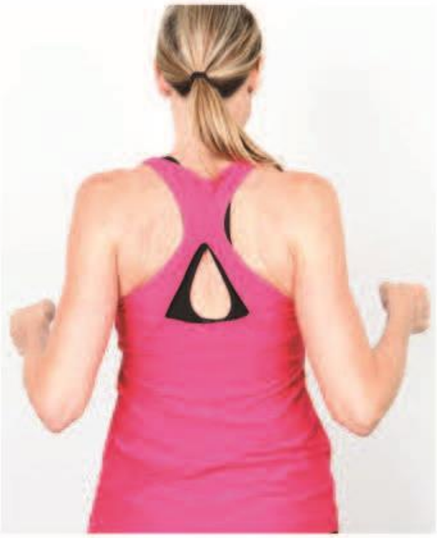

SCAPULAR RETRACTION
Instruct patient to squeeze their shoulder blades together.

CRITERIA FOR PROGRESSION TO PHASE TWO

Patient must meet 2 of the 3 criteria.

1. Attain full PROM
2. Maximum rest pain (3/10 on VAS)
3. Maximum pain with ADLs (4/10 pain on VAS)

© 2019 Jain NB et al. *JAMA Network Open.*

PHASE TWO: Active Assisted Motion, Shoulder Stretches, Rotator Cuff Strengthening & Scapular Stabilization

GOALS OF PHASE TWO

- Attain full AROM shoulder
- Patient will exhibit a minimum of 4/5 strength in the following muscle groups:
  - Internal rotators
  - External rotators
  - Serratus anterior
  - Middle trapezius
  - Lower trapezius
- Increase shoulder flexibility
- Improve scapulohumeral movement
- Eliminate rest pain and decrease pain with ADLs

The therapist should continue with manual therapy techniques during this phase.

Active assisted range of motion (AAROM) should be introduced during this phase. AAROM includes the use of a cane/stick and pulleys. Motions include flexion, abduction, external rotation, and internal rotation. Instruct the patient to use a cane/stick and/or pulleys to elevate or rotate the involved arm. The uninvolved arm should guide the involved arm. The patient should increase the elevation or rotation of the involved arm as tolerated. AAROM should be done at each therapy visit.

The therapist should introduce rotator cuff and scapular stabilization and rhythmic stabilization exercises. These exercises should be done at each therapy visit. Exercises may start with bands or no weights and progress to hand weights. All exercises should be performed while squeezing the shoulder blades together. It is important to avoid "hiking" of the shoulder. These exercises should be performed at home at least three days per week. The patient should feel muscle fatigue toward the end of the exercise but still be able to perform the exercise with good form and no pain. The therapist should introduce shoulder stretches during this phase. These exercises should be performed at each therapy visit as well as at home. Ice may be used as needed.

© 2019 Jain NB et al. *JAMA Network Open.*

PHASE TWO

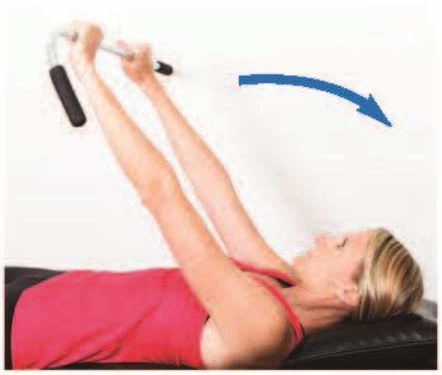

FLEXION

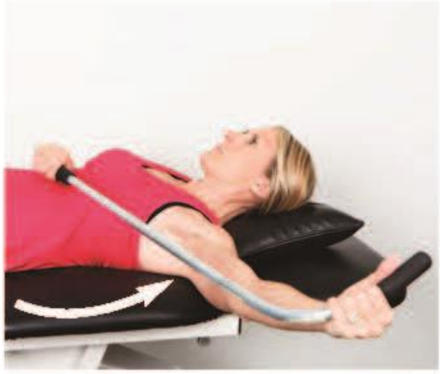

ABDUCTION

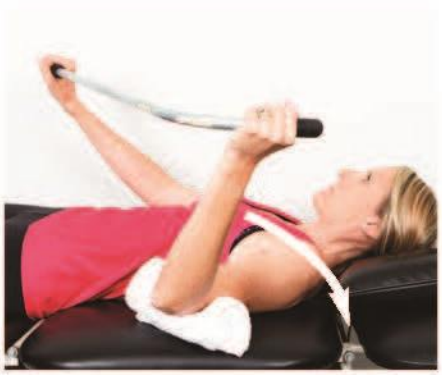

EXTERNAL ROTATION

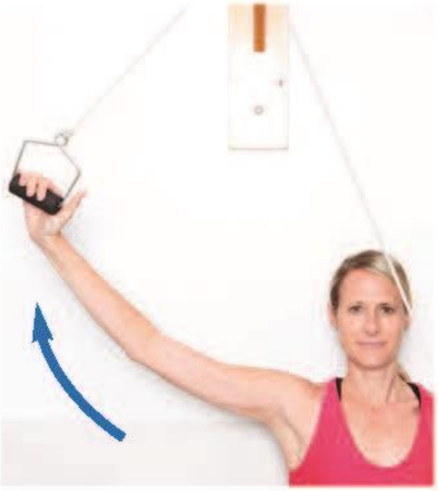

ABDUCTION

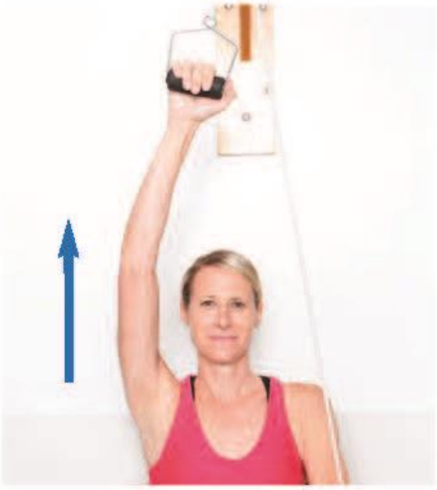

FLEXION

© 2019 Jain NB et al. *JAMA Network Open.*

PHASE TWO

The patient may begin with gentle stretching and progress as tolerated. Stretching should be done at each therapy visit. Perform 5 repetitions and hold each stretch for 20 seconds.

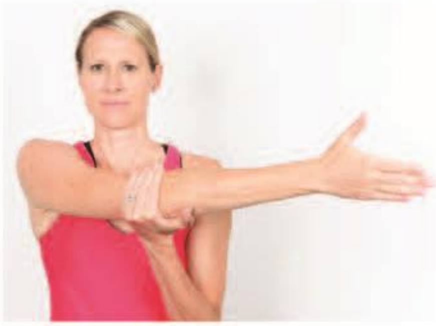

CROSS BODY STRETCH

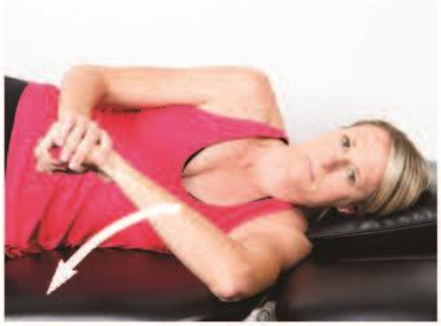

SLEEPER STRETCH

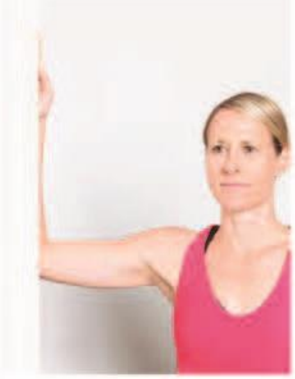

CORNER STRETCH

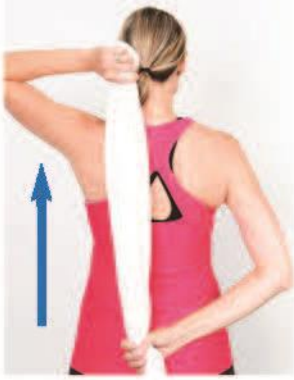

TOWEL STRETCH

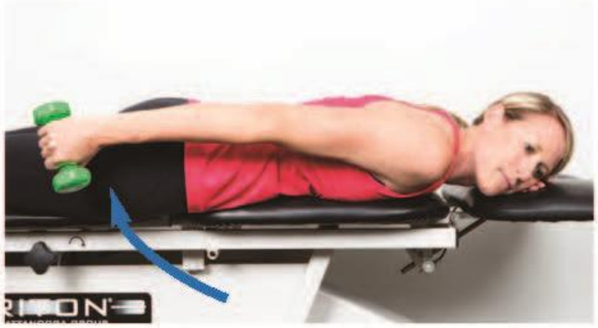

PRONE SHOULDER EXTENSION

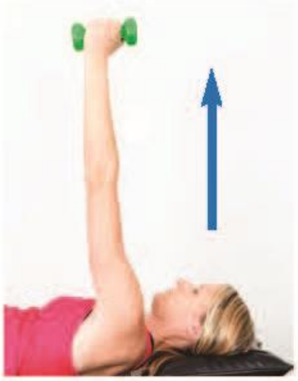

SERRATUS PUNCH

© 2019 Jain NB et al. *JAMA Network Open.*

PHASE TWO

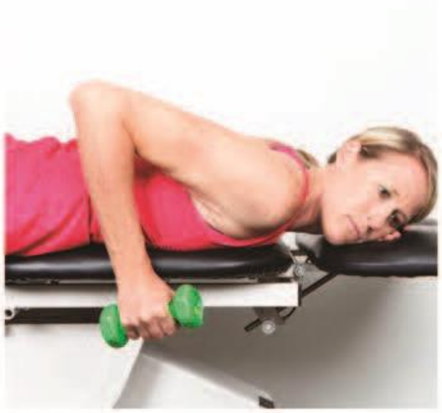

PRONE ROW

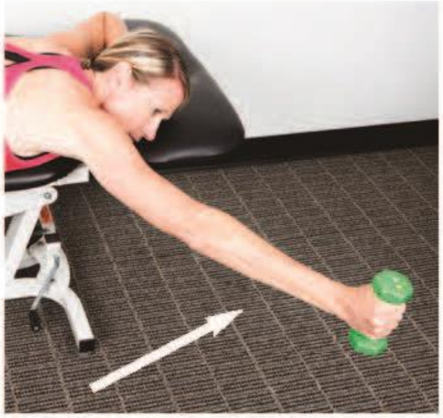

PRONE SCAPTION

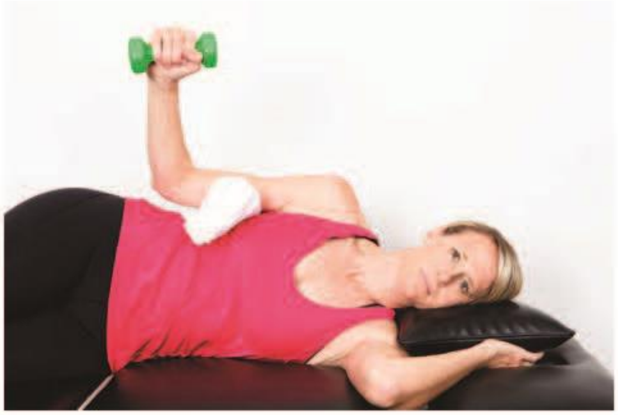

SIDELYING EXTERNAL ROTATION

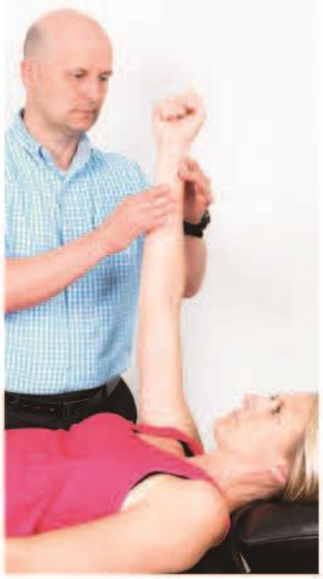

RHYTHMIC STABILIZATION WITH MANUAL RESISTANCE

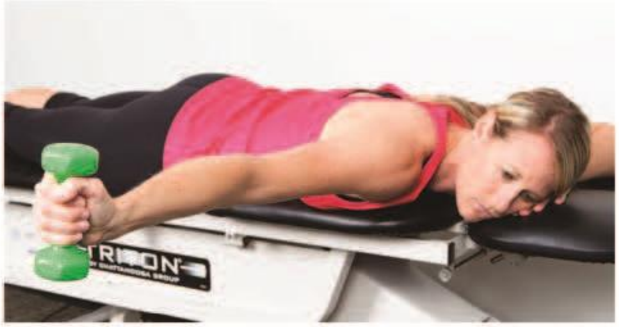

PRONE HORIZONTAL ABDUCTION

© 2019 Jain NB et al. *JAMA Network Open.*

PHASE TWO

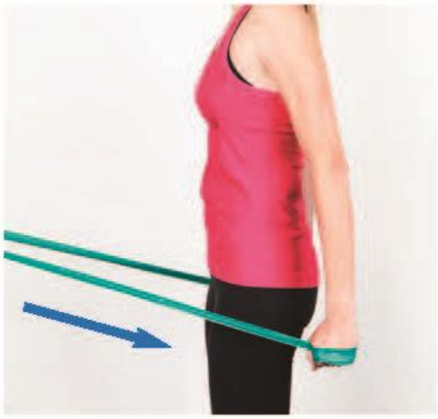

THERABAND SHOULDER EXTENSION

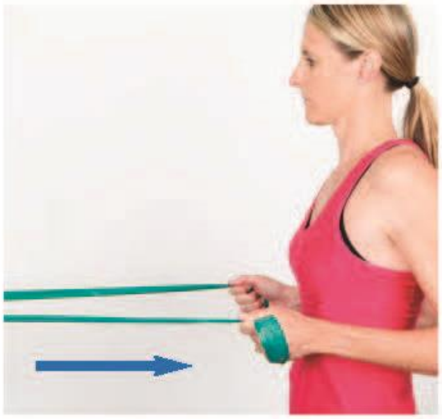

THERABAND ROW

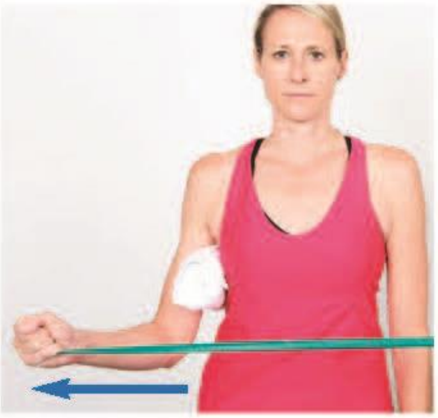

THERABAND EXTERNAL ROTATION

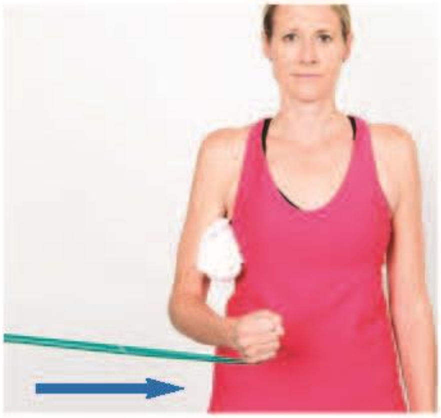

THERABAND INTERNAL ROTATION

© 2019 Jain NB et al. *JAMA Network Open.*

PHASE TWO

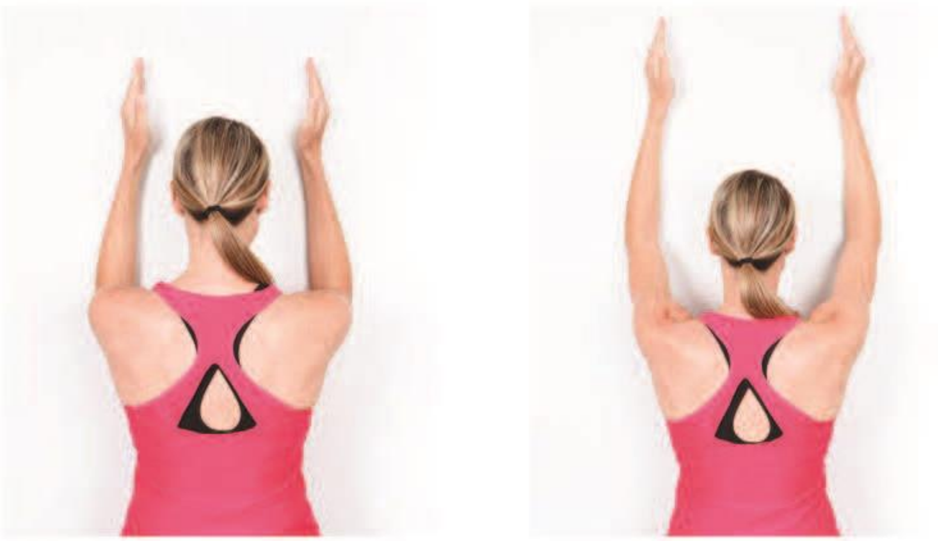

WALL SLIDES

CRITERIA FOR PROGRESSION TO PHASE THREE

Patient must meet 3 of the 4 criteria.

1. Attain full AROM shoulder
2. Patient exhibits a minimum of 4/5 strength in the following muscle groups:
   - Internal rotators
   - External rotators
   - Serratus anterior
   - Middle trapezius
   - Lower trapezius
3. Eliminate shoulder pain at rest
4. Decrease shoulder pain (2/10 on VAS) with ADLs

© 2019 Jain NB et al. *JAMA Network Open.*

PHASE THREE: Advanced Scapular Stabilization, Rotator Cuff Strengthening & Rhythmic Stabilization

GOALS OF PHASE THREE

- Patient will exhibit a minimum of 4+/5 strength in the following muscle groups:
  - Internal rotators
  - External rotators
  - Serratus anterior
  - Middle trapezius
  - Lower trapezius
- Maintain flexibility
- Improve proprioception
- Normalize scapulohumeral movement with no substitution patterns
- Return to normal ADLs, sports, work without limitations

The therapist should continue to focus on scapular stabilization and rotator cuff strengthening as well as shoulder stretching exercises. The therapist may advance the program to include advanced scapular stabilization, rotator cuff strengthening and rhythmic stabilization exercises as tolerated by the patient. Ice may be used as needed. Patient may advance to perform both overhead and plyometric exercises when appropriate in order to return to work, sport or functional tasks.

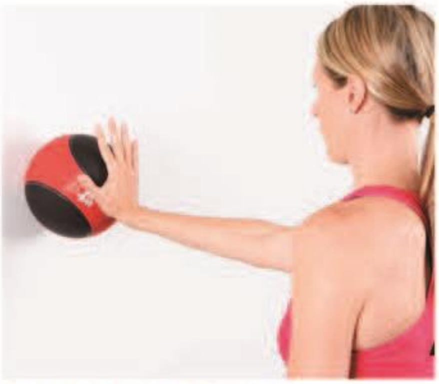

BALL ON WALL FLEXION

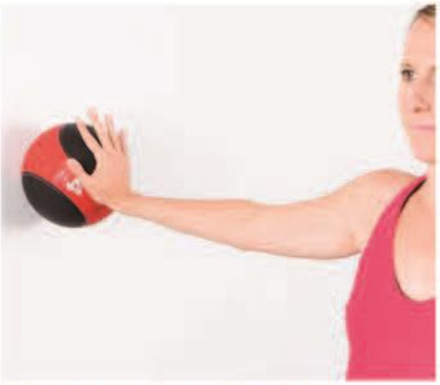

BALL ON WALL ABDUCTION

INSTRUCTIONS: The patient should place a ball against the wall at shoulder height, holding it with the palm of their hand, and perform small circles both clockwise and counterclockwise.

© 2019 Jain NB et al. *JAMA Network Open.*

PHASE THREE

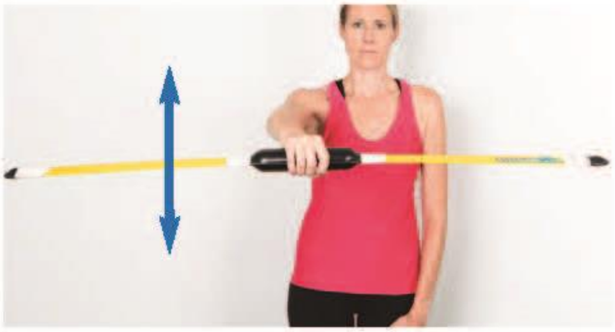

BODY BLADE FLEXION

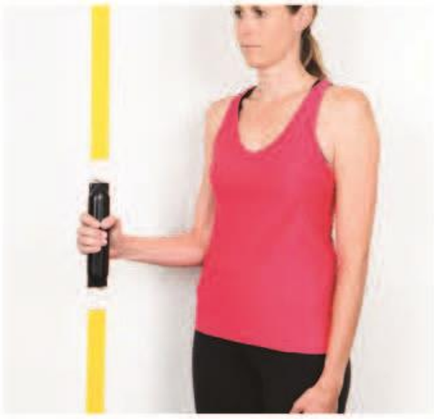

BODY BLADE EXTERNAL/INTERNAL ROTATION

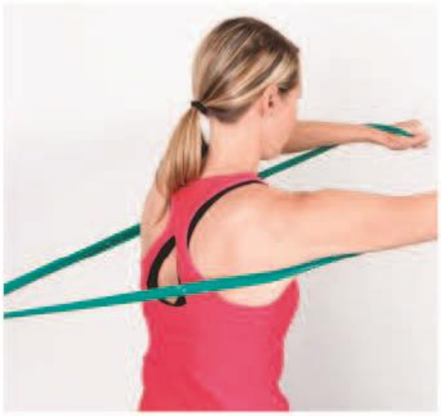

DYNAMIC HUGS

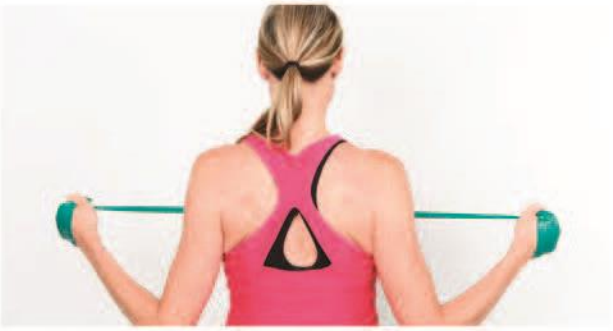

THERABAND SCAPULAR RETRACTION

© 2019 Jain NB et al. *JAMA Network Open.*

PHASE THREE

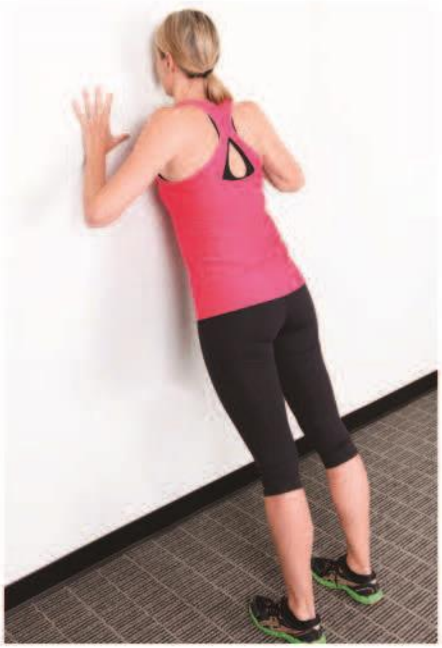

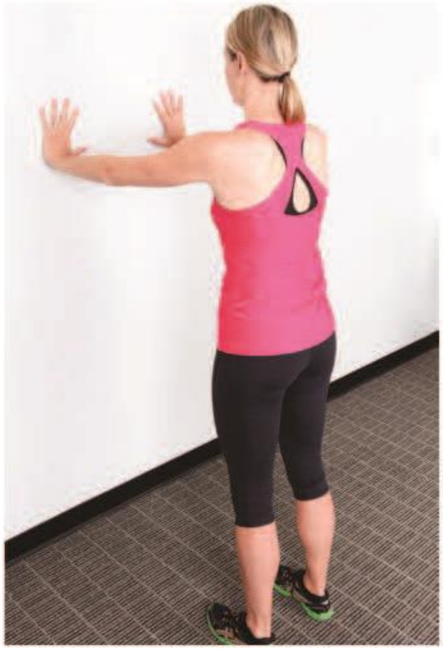

PUSHUPS WITH A PLUS AGAINST WALL

CRITERIA FOR DISCHARGE

Patient must meet 4 of the 5 criteria.

1. Full AROM of the involved shoulder
2. Patient exhibits a minimum of 4+/5 strength in the following muscle groups:
   - Internal rotators
   - External rotators
   - Serratus anterior
   - Middle trapezius
   - Lower trapezius
3. Normalize scapulohumeral movement with no substitution patterns
4. No shoulder pain at rest or with ADLs
5. Return to prior level of ADLs and/or sport

© 2019 Jain NB et al. *JAMA Network Open.*

PRIMARY STUDY INVESTIGATORS

Vanderbilt University Medical Center (Nashville, TN)

Nitin Jain, MD, MSPH – Trial Principal Investigator

John E. Kuhn, MD – Trial Co-Investigator, Surgical Core Leader

Kristin R. Archer, PhD, DPT – Trial Co-Investigator, Rehabilitation Core Leader

Brian Richardson, PT, MS, SCS, CSCS – Trial Lead Physical Therapist

Rebecca Dickinson, DPT, COMT – Site Lead Physical Therapist

Helen Koudelková, MA – Trial Project Manager

PARTNER SITES

The Ohio State Wexner Medical Center (Columbus, OH)

Andrew Neviaser, MD – Site Principal Investigator

Mitch Salsbery, PT, DPT, SCS – Site Lead Physical Therapist

Orthopedic Institute (Sioux Falls, SD)

Keith M. Baumgarten, MD – Site Principal Investigator

Matthew Zens, DPT, MS, SCS, ATC – Site Lead Physical Therapist

Knoxville Orthopaedic Clinic (Knoxville, TN)

Edwin E. Spencer, Jr., MD – Site Principal Investigator

Garrett Rich, DPT – Site Lead Physical Therapist

University of California San Francisco Orthopaedic Institute (San Francisco, CA)

C. Benjamin Ma, MD – Site Principal Investigator

Sarah Pawlowsky, PT, DPT, OCS – Site Lead Physical Therapist

University of Colorado-CU Sports Medicine (Denver & Boulder, CO)

Eric McCarty, MD – Site Principal Investigator

Anthony Kinney, PT, DPT, OCS, FAAOMPT – Site Lead Physical Therapist (Denver)

Pamela Andringa, MSPT – Site Lead Physical Therapist (Boulder)

University of Iowa (Iowa City, IA)

Brian R. Wolf, MD, MS - Site Principal Investigator

Mike Shaffer, PT, MSPT, OCS, ATC – Site Lead Physical Therapist

© 2019 Jain NB et al. *JAMA Network Open.*

University of Kentucky Healthcare, Orthopaedics Surgery & Sports Medicine (Lexington, KY)
Carolyn M. Hettrich, MD, MPH – Site Principal Investigator
Tim L. Uhl, PhD, PT, ATC - Site Lead Physical Therapist

University of Michigan Medicine (Ann Arbor, MI)
Bruce Miller, MD – Site Principal Investigator
Corey Snyder, PT, OCS, SCS, MTC, CSCS – Site Lead Physical Therapist

University of Pennsylvania Medical Center (Philadelphia, PA)
John D. Kelly, IV, MD –Site Principal Investigator
Marisa Pontillo, PT, PhD, DPT, SCS – Site Lead Physical Therapist

University of Texas Southwestern Medical Center (Dallas, TX)
Michael Khazzam, MD – Site Principal Investigator
Edward P. Mulligan, PT, DPT, OCS, SCS, ATC – Site Lead Physical Therapist

Washington University (St. Louis, MO)
Matthew V. Smith, MD, MSc – Site Principal Investigator
Suzanne Schroeder, PT, ATC – Site Lead Physical Therapist

CONSULTANTS

Paula Ludewig, PT, PhD, FAPTA – Rehabilitation Consultant (University of Minnesota Medical School, Department of Rehabilitation Medicine, Minneapolis, MN)
Leslie Burton, PT – Community Consultant (Star Physical Therapy, Nashville, TN)

© 2019 Jain NB et al. *JAMA Network Open.*

Research reported in this brochure was partially funded through a Patient-Centered Outcomes Research Institute (PCORI) Award (1605-35413). The statements presented in this brochure are solely the responsibility of the author(s) and do not necessarily represent the views of the Patient-Centered Outcomes Research Institute (PCORI), its Board of Governors or Methodology Committee.

Research reported in this publication was supported by the National Institute of Arthritis and Musculoskeletal and Skin Diseases of the National Institutes of Health under award number U34AR069201. The content is solely the responsibility of the authors and does not necessarily represent the official views of the National Institutes of Health.

The project brochure described was supported by CTSA award No. UL1TR000445 from the National Center for Advancing Translational Sciences. Its contents are solely the responsibility of the authors and do not necessarily represent official views of the National Center for Advancing Translational Sciences or the National Institutes of Health.

© 2019 Jain NB et al. *JAMA Network Open.*

© 2019 Jain NB et al. *JAMA Network Open.*

Arthroscopic Rotator Cuff (ARC) Clinical Trial

NONOPERATIVE REHABILITATION PROTOCOL

ARC Clinical Trial is led by:

Vanderbilt Physical Medicine & Rehabilitation

Vanderbilt Sports Medicine

Vanderbilt Orthopaedic Institute

ShoulderStudy.com

© Vanderbilt University Medical Center, 2017

© 2019 Jain NB et al. *JAMA Network Open.*

eAppendix 2. ARC Surgical Protocol

An evidence-based, standard surgical protocol will maximize the potential for rotator cuff tear repair healing, and will reduce variables that could affect the outcome in this randomized trial. As such, every surgeon performing rotator cuff repair as part of this trial will adhere to the surgical protocol as described below:

Tear Patterns:

While each patient's tear is unique, there are specific patterns that have been recognized that direct appropriate repair technique (Burkhart 2000). Crescent shaped tears are repaired directly to the greater tuberosity. L-Shaped tears are reduced, and sutures are placed in the longitudinal portion of the tear, while the horizontal portion is repaired to the greater tuberosity. V-Shaped tears are repaired via margin convergence sutures in the apex, then the base of the tear is repaired to the tuberosity.

Preparation of the Tuberosity:

In order to assure the best environment for healing, the greater tuberosity will be debrided of all soft tissue and lightly decorticated.

Repair to the Greater Tuberosity:

Double row repairs or transosseous equivalent repairs will be used for large to massive rotator cuff tears as the pooled data supports this technique when compared to single row repairs (Spiegl 2016), Hein 2015, Mascarenhas 2014, Millett 2014) for improved healing rates. In tears too small for double row or transossous equivalent techniques, single row repairs will be performed. In massive tears, where a double row repair is not possible, margin convergence and single row repair will be employed.

Materials for Repair

All sutures for rotator cuff repair will be #2 kevlar impregnated suture or equivalent high strength #2 suture. Anchors will be PEEK or equivalent, or all suture-based, Biocomposite, or Bioabsorbable anchors which have been demonstrated to have adequate pull out strength, and do not interfere with MRI images.

Acromioplasty:

At the surgeon's discretion, if acromion spurring must be removed for visualization, an acromioplasty will be performed.

Biceps Pathology:

At the surgeon's discretion, if significant biceps pathology is encountered, a biceps release or tenodesis will be performed.

© 2019 Jain NB et al. *JAMA Network Open.*

Acromioclavicular Joint Pathology:

Symptomatic acromioclavicular joint osteoarthritis may be treated by an arthroscopic distal clavicle excision at the surgeon's discretion.

Partial Tears Undergoing Surgery:

Partial tears may be treated with debridement, trans-tendonous repair, repair using anchors to the tuberosity, or completion of the tear to a small full thickness tear. In general, if the tendon cannot be repaired, it will be debrided. When a repair is undertaken, the transtendon approach will be preferred if possible as there is some literature to support that this technique has a lower re-tear rate than conversion to a full thickness repair (Sun 2015, Kim 2015).

Literature Cited:

Burkhart SS. Current concepts. A stepwise approach to arthroscopic rotator cuff repair based on biomechanical principles *Arthroscopy*, 16 (2000), pp. 82–90.

Spiegl UJ, Euler SA, Millett PJ, Hepp P. Summary of Meta-Analyses Dealing with Single-Row versus Double-Row Repair Techniques for Rotator Cuff Tears. Open Orthop J. 2016 Jul 21;10:330-338. PMID: 27708735

Hein J, Reilly JM, Chae J, Maerz T, Anderson K. Retear Rates After Arthroscopic Single-Row, Double-Row, and Suture Bridge Rotator Cuff Repair at a Minimum of 1 Year of Imaging Follow-up: A Systematic Review. Arthroscopy. 2015 Nov;31(11):2274-81. doi: 10.1016/j.arthro.2015.06.004. Epub 2015 Jul 15. Review. PMID: 26188783

Mascarenhas R, Chalmers PN, Sayegh ET, Bhandari M, Verma NN, Cole BJ, Romeo AA. Is double-row rotator cuff repair clinically superior to single-row rotator cuff repair: a systematic review of overlapping meta-analyses. Arthroscopy. 2014 Sep;30(9):1156-65. doi: 10.1016/j.arthro.2014.03.015. Epub 2014 May 10. Review. PMID: 24821226

Millett PJ, Warth RJ, Dornan GJ, Lee JT, Spiegl UJ. Clinical and structural outcomes after arthroscopic single-row versus double-row rotator cuff repair: a systematic review and meta-analysis of level I randomized clinical trials. J Shoulder Elbow Surg. 2014 Apr;23(4):586-97. doi: 10.1016/j.jse.2013.10.006. Epub 2014 Jan 8. Review. PMID: 24411671

Sun L, Zhang Q, Ge h, Sun Y, Cheng B. Which is the best repair of articular-sided rotator cuff tears: a meta-analysis, J Orthop Surg Res. 2015 May 28;10:84. doi: 10.1186/s13018-015-0224-6.

Kim YS, Lee HJ, Bae SH, Jin H, Song HS. Outcome Comparison Between in Situ Repair Versus Tear Completion Repair for Partial Thickness Rotator Cuff Tears. Arthroscopy. 2015 Nov;31(11):2191-8. doi: 10.1016/j.arthro.2015.05.016. Epub 2015 Jul 15.

© 2019 Jain NB et al. *JAMA Network Open.*

eAppendix 3. Postoperative Rehabilitation Protocol

Reproduced with permission of Vanderbilt University Medical Center

© 2019 Jain NB et al. *JAMA Network Open.*

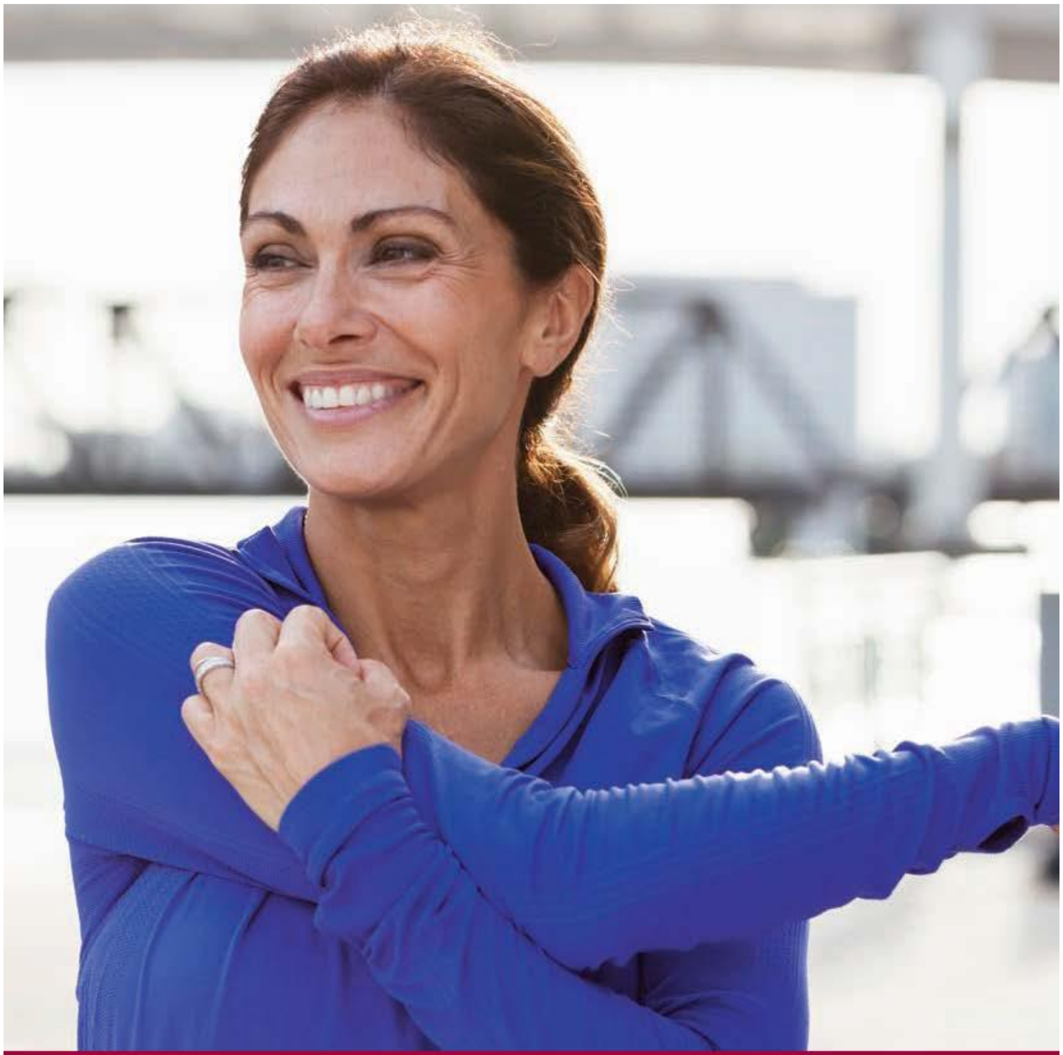

ARC
ARTHROSCOPIC ROTATOR CUFF TRIAL

POSTOPERATIVE REHABILITATION PROTOCOL

ShoulderStudy.com

© 2019 Jain NB et al. *JAMA Network Open.*

Arthroscopic Rotator Cuff (ARC) Clinical Trial

POSTOPERATIVE REHABILITATION PROTOCOL

TABLE OF CONTENTS

INTRODUCTION 1

GENERAL INSTRUCTIONS 2

PHASE ONE 4
Passive Motion & Scapular Exercises
(Weeks 0-4)

PHASE TWO 6
Supine Active Assisted Motion
(Week 4)

PHASE THREE 7
Active Assisted Motion & Scapular Exercises
(Weeks 5-8)

PHASE FOUR 9
Active Motion, Isometrics & Scapular Exercises
(Weeks 9-12)

PHASE FIVE 11
Resisted Exercises & Shoulder Stretches
(Weeks 13-16)

Study Investigators & Partner Sites 16

© 2019 Jain NB et al. *JAMA Network Open.*

Arthroscopic Rotator Cuff (ARC) Clinical Trial

POSTOPERATIVE REHABILITATION PROTOCOL

POSTOPERATIVE GUIDELINES

Do not add or skip any part of this program.
If you have questions or concerns, please contact
the Lead Physical Therapist for your site below.

You may also contact the trial's Lead Physical Therapists at Vanderbilt:

Brian Richardson: brian.richardson@vanderbilt.edu
Rebecca Dickinson: rebecca.dickinson@vanderbilt.edu

© 2019 Jain NB et al. *JAMA Network Open.*

GENERAL INSTRUCTIONS:

- The patient should work with the physical therapist approximately 1-2x/week for 16 weeks. There is no required minimum number of visits per week.
- The patient should perform a home exercise program (HEP) as prescribed by the physical therapist approximately 30 minutes/day, 2-4x/week.
- The combined total of physical therapy visits & HEP (in any combination) should equal 4+x/week.

Timeline

The patient is to begin physical therapy in the first week after surgery.

Modalities

Patients are encouraged to use cryotherapy after surgery and exercise.

Sling Use

A sling with a small pillow may be worn for 6 weeks after surgery. The sling may be removed for showering and activities as directed. The sling should be worn when the patient is in an uncontrolled environment: sleeping, around children, pets, and crowds during these six weeks.

Activity Restrictions

- Showering: As directed by physician
- Deskwork: When comfortable with sling
- Driving: As directed by physician
- Lifting restrictions:
  After 9 weeks: Up to 1-2 pounds below shoulder level
  After 12 weeks: Up to 5 pounds keeping weight close to body
- Reaching behind back: 9 weeks
- Pushing/Pulling: 12 weeks
- Reaching overhead: 12 weeks
- Return to Sport/Heavy Activity: Upon the completion of the therapy program as advised by the physician and physical therapist

© 2019 Jain NB et al. *JAMA Network Open.*

Each phase should include:

- Physical therapy (PT) intervention as indicated by treating physical therapist's plan of care within guidelines of this study protocol
- Perform Home Exercise Program (HEP)

Progression

- For each exercise, begin with 1 set (up to 10 repetitions per set) and progress to 3 sets (up to 10 repetitions per set). The number of sets/reps given should be based on good quality movement.
- The post-operative guidelines should be advanced per the time period indicated for each phase.
- The patient should not be advanced to the next phase sooner than specified.

STAGED ROM GOALS AND APPROXIMATE TARGETS

	Passive Forward Flexion	Passive External Rotation at 20° Abduction	Passive External Rotation at 90° Abduction	Active Forward Flexion
Week 2	60°-90°	0°-20°	NA	NA
Week 6	90°-120°	20°-30°	NA	NA
Week 9	130°-155°	30°-45°	45°-60°	80°-120°
Week 12	140°-WNL	30°-WNL	75°-WNL	120°-WNL

© 2019 Jain NB et al. *JAMA Network Open.*

PHASE ONE: Passive Motion & Scapular Exercises (Weeks 0-4)

GOALS OF PHASE ONE:

- Protect the repair
- Allow for wound healing
- Control pain and inflammation
- Prevent the development of adhesions

Passive range of motion (PROM), pendulum exercises and scapular retraction are started during the first week after surgery. PROM requires the therapist to put the arm through a comfortable range of motion while the patient is supine. Motions include flexion, abduction and external rotation. Hand, wrist, and elbow motion should be done as needed. PROM, pendulum, and scapular retraction should be done at each therapy visit.

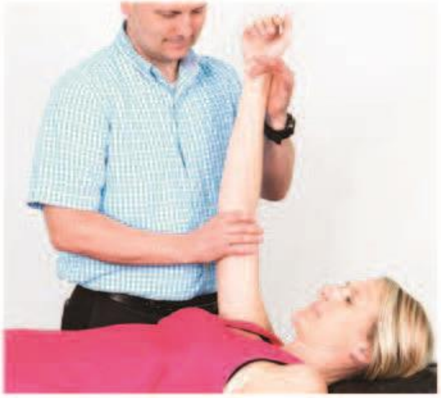

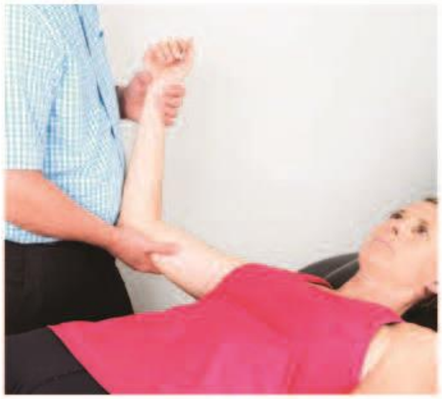

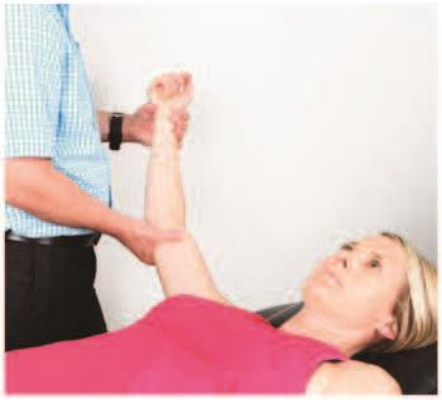

PROM: The therapist should move the arm while the patent remains relaxed.

© 2019 Jain NB et al. *JAMA Network Open.*

PHASE ONE

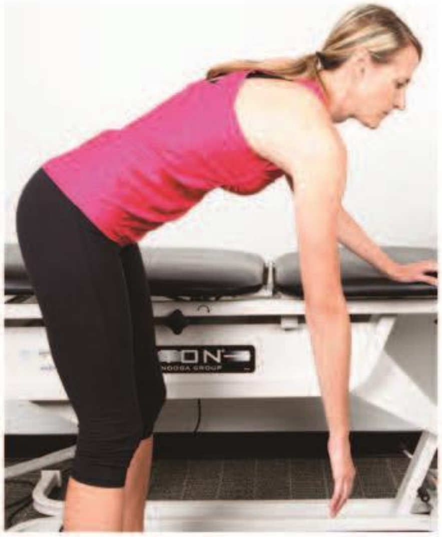

PENDULUM EXERCISES
Clockwise/Counterclockwise, forward back and side/side. Keep the arm relaxed and move at the hips and trunk.

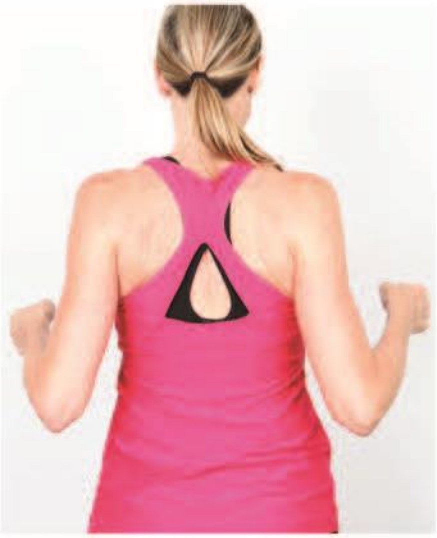

SCAPULAR RETRACTION
Instruct patient to squeeze their shoulder blades together.

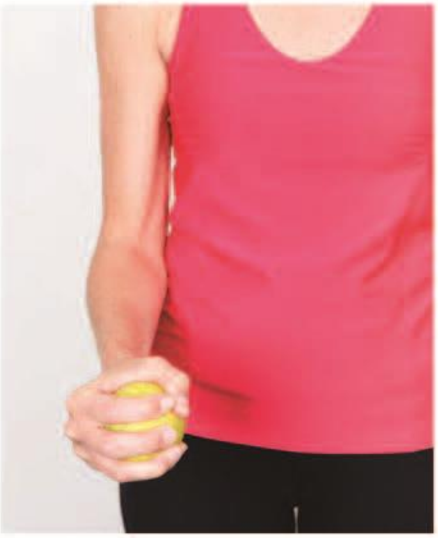

GRIPPING
Instruct patient to squeeze a towel or a therapy ball. Hold 5 seconds.

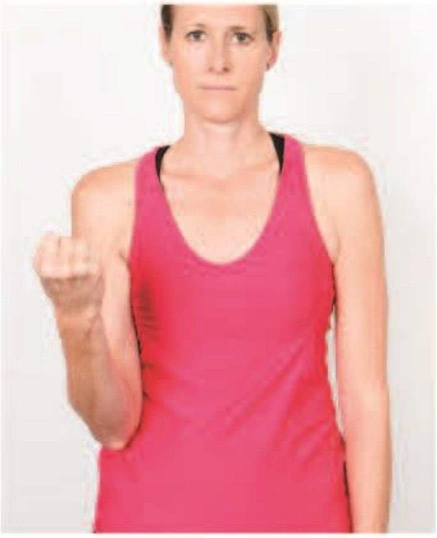

ELBOW FLEXION
Instruct patient to flex and extend the elbow.

© 2019 Jain NB et al. *JAMA Network Open.*

PHASE TWO: Supine Active Assisted Motion (Week 4)

GOALS OF PHASE TWO & THREE:

- Protect the repair
- Attain PROM per staged ROM goals
- Control pain and inflammation

At Week 4, the therapist should introduce active assisted range of motion (AAROM) in the supine position. Motions include flexion, abduction and external rotation. AAROM should be done at each therapy visit.

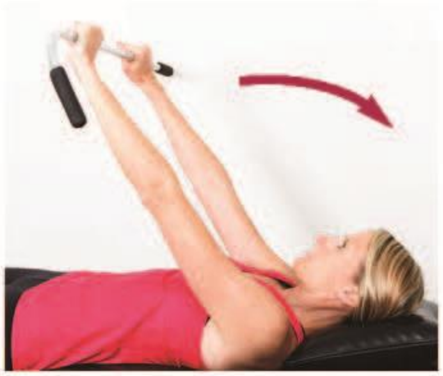

FLEXION

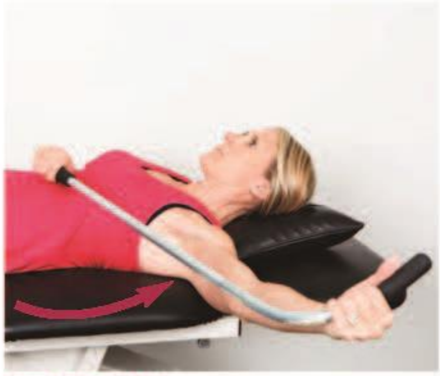

ABDUCTION

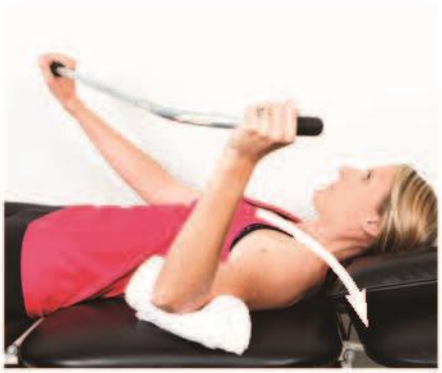

EXTERNAL ROTATION

INSTRUCTIONS
The patient should use a cane/stick to elevate or rotate the involved arm. The uninvolved arm should guide the involved arm. The patient should increase the elevation or rotation of the involved arm as tolerated.

© 2019 Jain NB et al. *JAMA Network Open.*

PHASE THREE: Active Assisted Motion & Scapula Exercises (Weeks 5-8)

At week 5, the therapist should introduce AAROM with the patient at an incline of 45 degrees. At week 6, the therapist should have the patient perform AAROM in the upright position using a cane/stick and pulleys. Motions include flexion, abduction and external rotation. Scapular retraction exercises should continue during this time period. AAROM and scapular retraction should be done at each therapy visit.

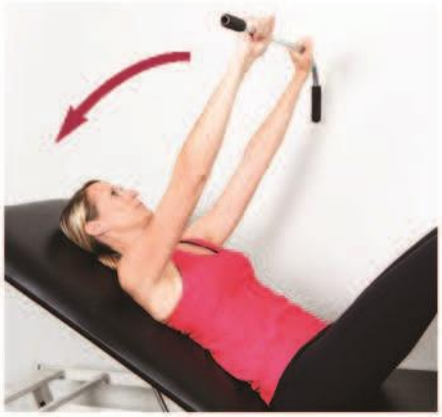

FLEXION

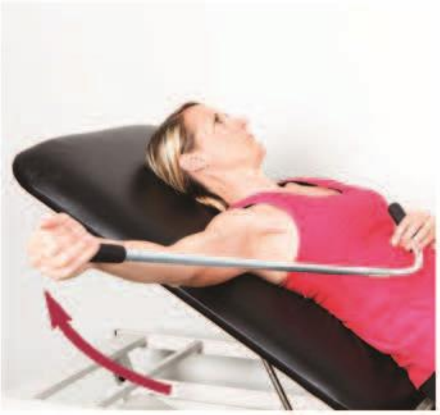

ABDUCTION

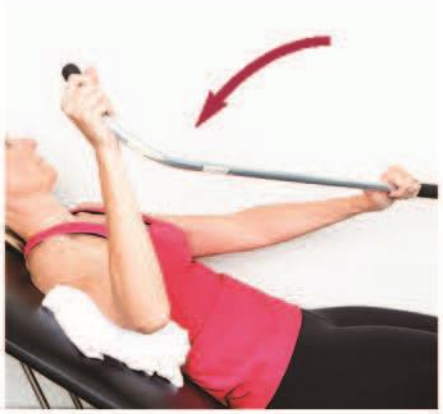

EXTERNAL ROTATION

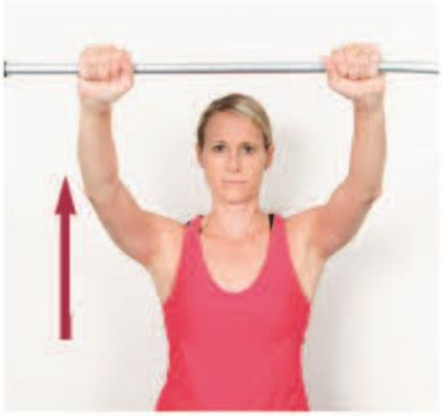

FLEXION

© 2019 Jain NB et al. *JAMA Network Open.*

PHASE THREE

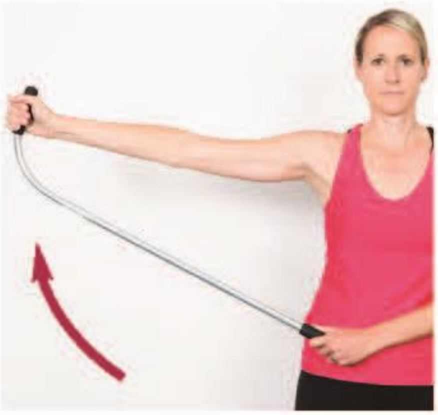

ABDUCTION

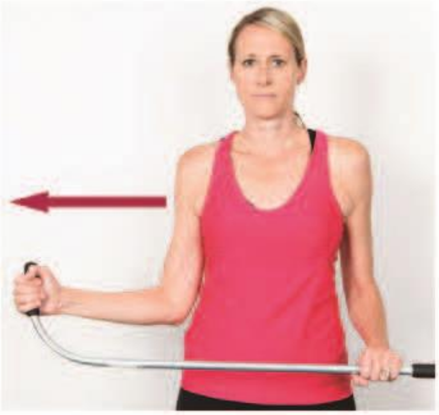

EXTERNAL ROTATION

INSTRUCTIONS

The patient should use a cane/stick or pulleys to elevate or rotate the involved arm. The uninvolved arm should guide the involved arm. The patient should increase the elevation or rotation of the involved arm as tolerated.

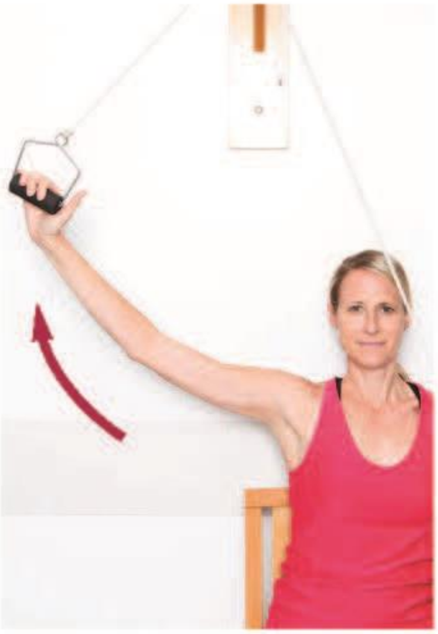

ABDUCTION

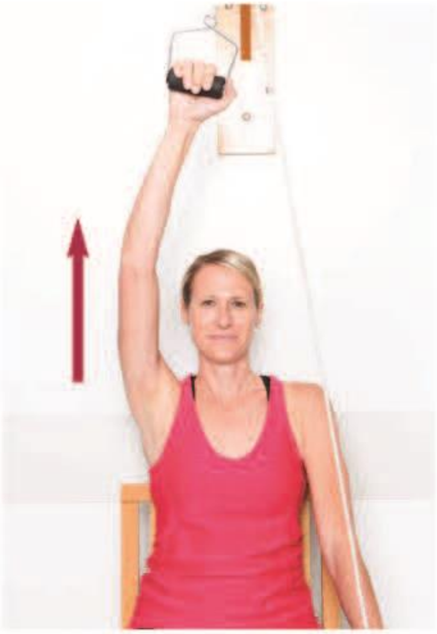

FLEXION

© 2019 Jain NB et al. *JAMA Network Open.*

PHASE FOUR: Active Motion, Isometrics & Scapula Exercises (Weeks 9-12)

GOALS OF PHASE FOUR:

- Maintain full PROM
- Initiate strengthening program
- Attain full, pain-free AROM

The patient begins active range of motion (AROM) in flexion, abduction and external rotation. In addition, isometric strengthening exercises begin at this time. The patient should continue with scapular retraction exercises. PROM should continue as needed. AROM, isometrics and scapular exercises should be done at each therapy visit.

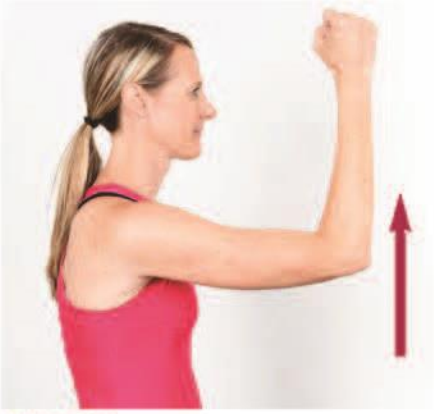

FLEXION

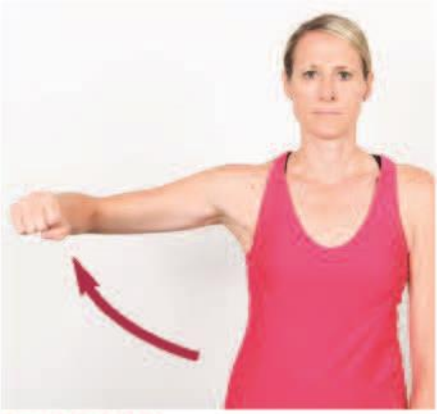

ABDUCTION

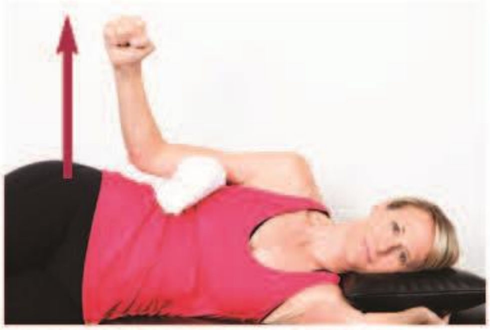

SIDELYING EXTERNAL ROTATION

INSTRUCTIONS

In standing, the patient should move the involved arm forward into elevation and abduction. If needed, the patient may begin these exercises with the elbow bent. It is important to avoid "hiking" of the shoulder. In sidelying, the patient should move the arm into external rotation.

© 2019 Jain NB et al. *JAMA Network Open.*

PHASE FOUR

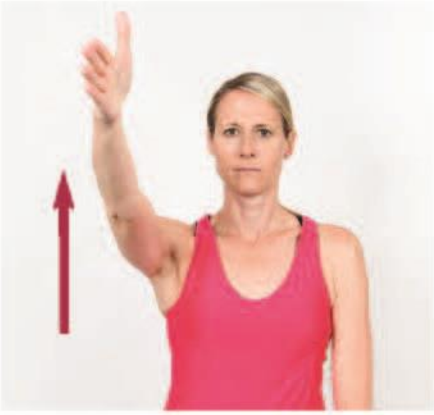

FLEXION

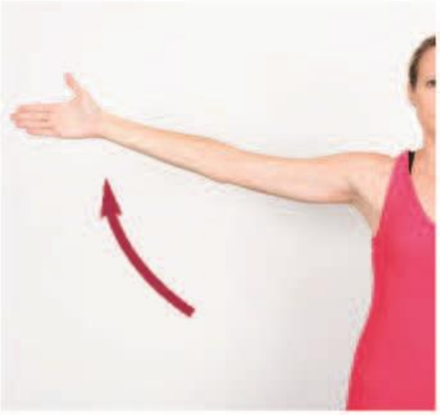

ABDUCTION

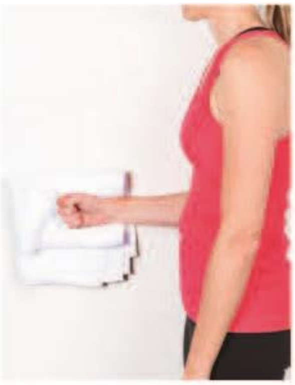

EXTERNAL ROTATION

INSTRUCTIONS
In standing with the elbow bent at 90 degrees, the patient should use a towel for sub-maximal isometrics. The patient should push the involved hand out against the wall for external rotation, into the wall for internal rotation, forward for flexion, and the involved elbow back against the wall for extension. Hold for 10 seconds.

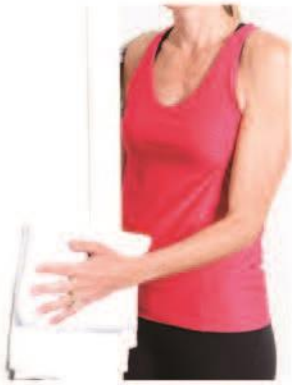

INTERNAL ROTATION

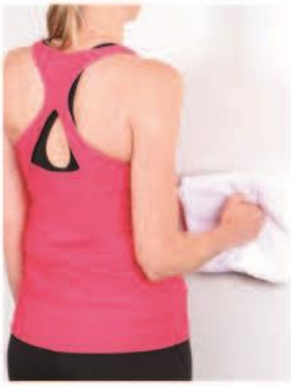

FLEXION

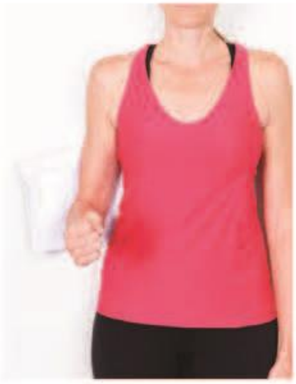

EXTENSION

© 2019 Jain NB et al. *JAMA Network Open.*

PHASE FIVE: Resisted Exercises & Shoulder Stretches (Weeks 13-16)

GOALS OF PHASE FIVE:

- Maintain full, pain-free AROM
- Increase functional activities
- Restore strength in involved UE

At week 13, the therapist should introduce resisted scapular stabilization and rotator cuff strengthening. Exercises may start with bands or no weights and progress to hand weights. All exercises should be performed while squeezing the shoulder blades together. These exercises should be performed at least three days per week. The patient should feel muscle fatigue toward the end of the exercise but still be able to perform the exercise with good form and no pain. Patient may advance to perform both overhead and plyometric exercises when appropriate in order to return to work, sport or functional tasks.

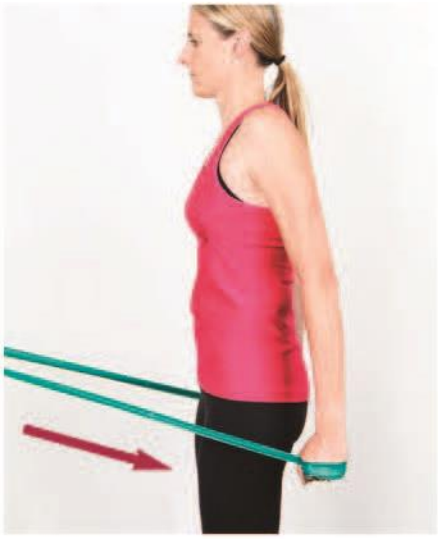

THERABAND SHOULDER EXTENSION

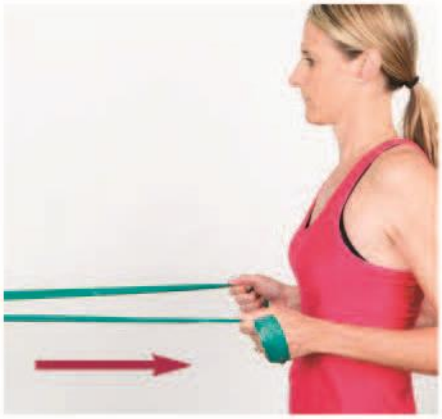

THERABAND ROW

© 2019 Jain NB et al. *JAMA Network Open.*

PHASE FIVE

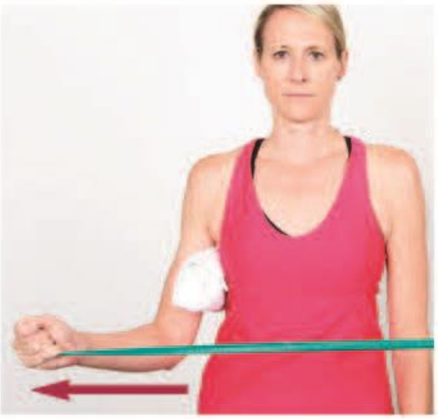

THERABAND EXTERNAL ROTATION

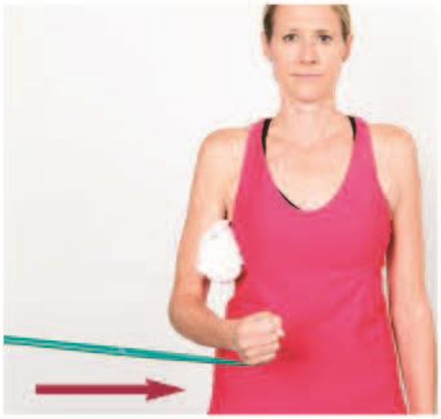

THERABAND INTERNAL ROTATION

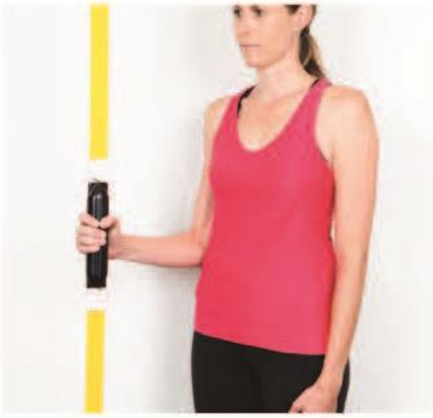

BODY BLADE EXTERNAL/INTERNAL ROTATION

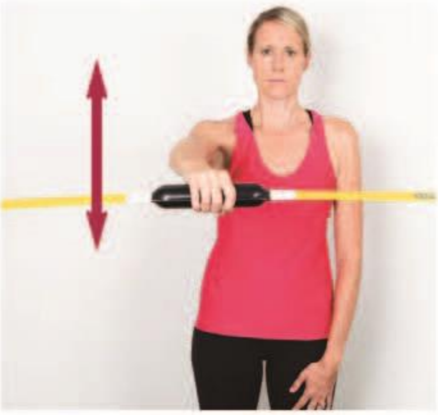

BODY BLADE FLEXION

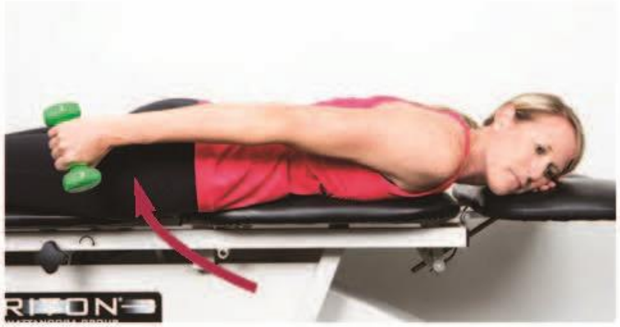

PRONE SHOULDER EXTENSION

© 2019 Jain NB et al. *JAMA Network Open.*

PHASE FIVE

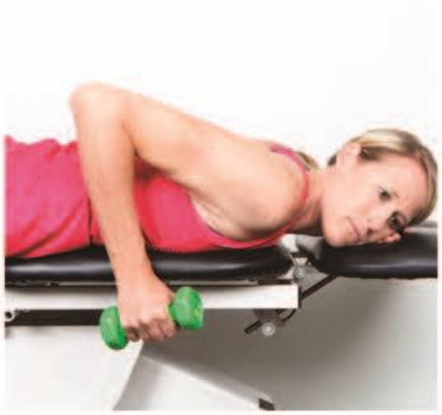

PRONE ROW

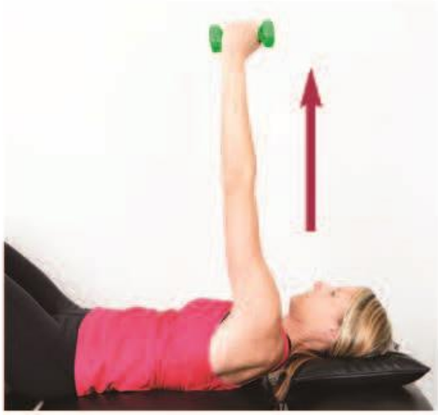

SERRATUS PUNCH

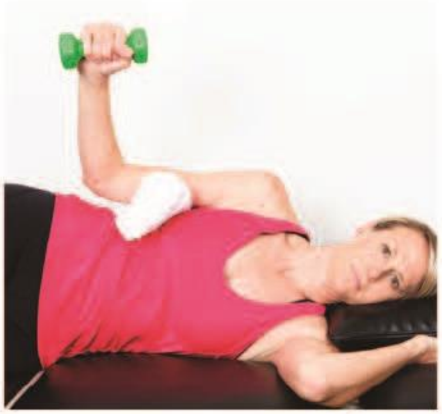

SIDELYING EXTERNAL ROTATION

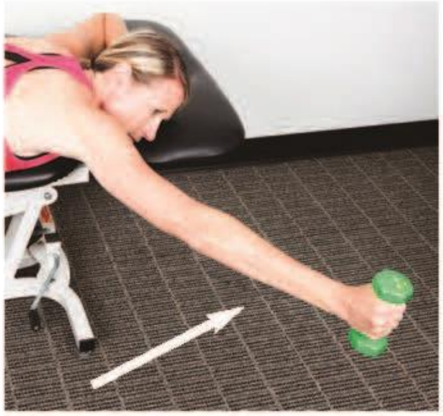

PRONE SCAPTION

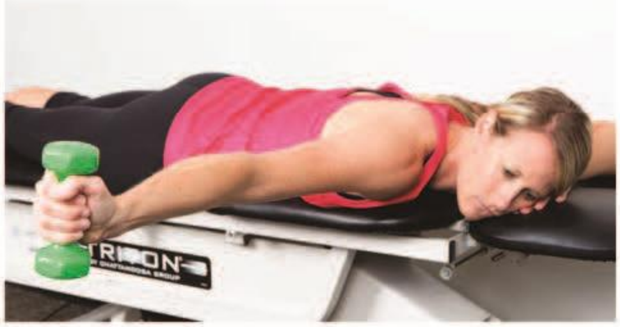

PRONE HORIZONTAL ABDUCTION

© 2019 Jain NB et al. *JAMA Network Open.*

PHASE FIVE

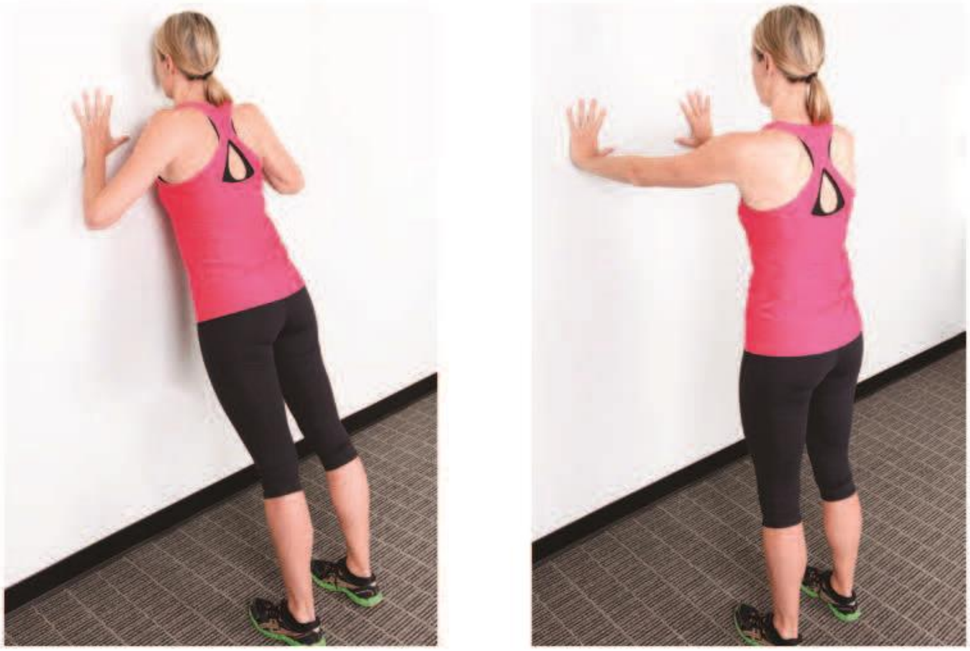

PUSHUPS WITH A PLUS AGAINST WALL

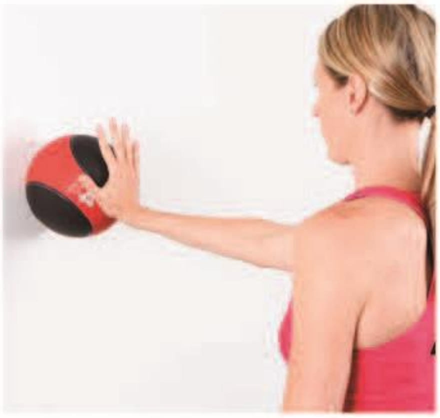

BALL ON WALL FLEXION

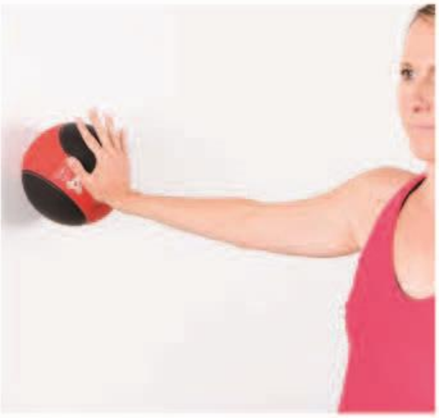

BALL ON WALL ABDUCTION

INSTRUCTIONS: The patient should place a ball against the wall at shoulder height, holding it with the palm of their hand, and perform small circles both clockwise and counterclockwise.

© 2019 Jain NB et al. *JAMA Network Open.*

PHASE FIVE

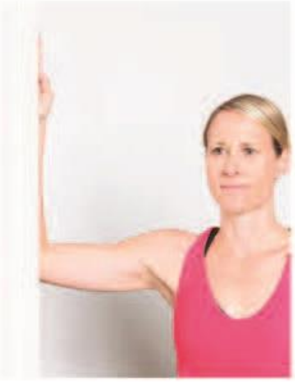

CORNER STRETCH

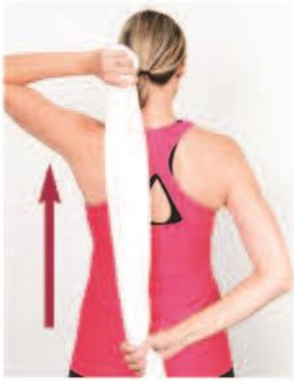

TOWEL STRETCH

INSTRUCTIONS
The patient may begin with gentle stretching and progress as tolerated. Stretching should be done at each therapy visit. Perform 5 repetitions and hold each stretch for 20 seconds.

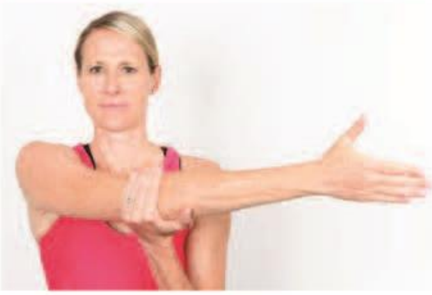

CROSS BODY STRETCH

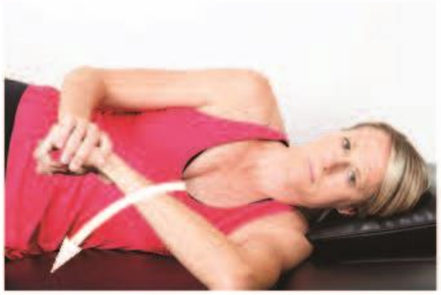

SLEEPER STRETCH

CRITERIA FOR DISCHARGE

Patient must meet 4 of the 5 criteria.

1. Full AROM of the involved shoulder
2. Patient exhibits a minimum of 4+/5 strength in the following muscle groups:
   - Internal rotators
   - External rotators
   - Serratus anterior
   - Middle trapezius
   - Lower trapezius
3. Normalize scapulohumeral movement with no substitution patterns
4. No shoulder pain at rest or with ADLs
5. Return to prior level of ADLs and/or sport

© 2019 Jain NB et al. *JAMA Network Open.*

PRIMARY STUDY INVESTIGATORS

Vanderbilt University Medical Center (Nashville, TN)

Nitin Jain, MD, MSPH – Trial Principal Investigator

John E. Kuhn, MD – Trial Co-Investigator, Surgical Core Leader

Kristin R. Archer, PhD, DPT – Trial Co-Investigator, Rehabilitation Core Leader

Brian Richardson, PT, MS, SCS, CSCS – Trial Lead Physical Therapist

Rebecca Dickinson, DPT, COMT – Site Lead Physical Therapist

Helen Koudelková, MA – Trial Project Manager

PARTNER SITES

The Ohio State Wexner Medical Center (Columbus, OH)

Andrew Neviaser, MD – Site Principal Investigator

Mitch Salsbery, PT, DPT, SCS – Site Lead Physical Therapist

Orthopedic Institute (Sioux Falls, SD)

Keith M. Baumgarten, MD – Site Principal Investigator

Matthew Zens, DPT, MS, SCS, ATC – Site Lead Physical Therapist

Knoxville Orthopaedic Clinic (Knoxville, TN)

Edwin E. Spencer, Jr., MD – Site Principal Investigator

Garrett Rich, DPT – Site Lead Physical Therapist

University of California San Francisco Orthopaedic Institute (San Francisco, CA)

C. Benjamin Ma, MD – Site Principal Investigator

Sarah Pawlowsky, PT, DPT, OCS – Site Lead Physical Therapist

University of Colorado-CU Sports Medicine (Denver & Boulder, CO)

Eric McCarty, MD – Site Principal Investigator

Anthony Kinney, PT, DPT, OCS, FAAOMPT – Site Lead Physical Therapist (Denver)

Pamela Andringa, MSPT – Site Lead Physical Therapist (Boulder)

University of Iowa (Iowa City, IA)

Brian R. Wolf, MD, MS - Site Principal Investigator

Mike Shaffer, PT, MSPT, OCS, ATC – Site Lead Physical Therapist

© 2019 Jain NB et al. *JAMA Network Open.*

University of Kentucky Healthcare, Orthopaedics Surgery & Sports Medicine (Lexington, KY)

Carolyn M. Hettrich, MD, MPH – Site Principal Investigator

Tim L. Uhl, PhD, PT, ATC - Site Lead Physical Therapist

University of Michigan Medicine (Ann Arbor, MI)

Bruce Miller, MD – Site Principal Investigator

Corey Snyder, PT, OCS, SCS, MTC, CSCS – Site Lead Physical Therapist

University of Pennsylvania Medical Center (Philadelphia, PA)

John D. Kelly, IV, MD –Site Principal Investigator

Marisa Pontillo, PT, PhD, DPT, SCS – Site Lead Physical Therapist

University of Texas Southwestern Medical Center (Dallas, TX)

Michael Khazzam, MD – Site Principal Investigator

Edward P. Mulligan, PT, DPT, OCS, SCS, ATC – Site Lead Physical Therapist

Washington University (St. Louis, MO)

Matthew V. Smith, MD, MSc – Site Principal Investigator

Suzanne Schroeder, PT, ATC – Site Lead Physical Therapist

CONSULTANTS

Paula Ludewig, PT, PhD, FAPTA – Rehabilitation Consultant (University of Minnesota Medical School, Department of Rehabilitation Medicine, Minneapolis, MN)

Leslie Burton, PT – Community Consultant (Star Physical Therapy, Nashville, TN)

Research reported in this brochure was partially funded through a Patient-Centered Outcomes Research Institute (PCORI) Award (1605-35413). The statements presented in this brochure are solely the responsibility of the author(s) and do not necessarily represent the views of the Patient-Centered Outcomes Research Institute (PCORI), its Board of Governors or Methodology Committee.

Research reported in this publication was supported by the National Institute of Arthritis and Musculoskeletal and Skin Diseases of the National Institutes of Health under award number U34AR069201. The content is solely the responsibility of the authors and does not necessarily represent the official views of the National Institutes of Health.

The project brochure described was supported by CTSA award No. UL1TR000445 from the National Center for Advancing Translational Sciences. Its contents are solely the responsibility of the authors and do not necessarily represent official views of the National Center for Advancing Translational Sciences or the National Institutes of Health.

© 2019 Jain NB et al. *JAMA Network Open.*

Arthroscopic Rotator Cuff (ARC) Clinical Trial

POSTOPERATIVE REHABILITATION PROTOCOL

ARC Clinical Trial is led by:

Vanderbilt Physical Medicine & Rehabilitation

Vanderbilt Sports Medicine

Vanderbilt Orthopaedic Institute

ShoulderStudy.com

© Vanderbilt University Medical Center, 2017

© 2019 Jain NB et al. *JAMA Network Open.*
